# Development of a Quantitative Serial LC-MS/MS Method for Gut Microbiota Metabolomics

**DOI:** 10.1021/acsomega.5c12997

**Published:** 2026-03-16

**Authors:** Takanobu Yoshida, Tomoya Shintani, Daisuke Sasaki, Christopher J. Vavricka, Yasushi Matsuki, Akihiko Kondo, Tomohisa Hasunuma

**Affiliations:** † Engineering Biology Research Center, 12885 Kobe University, 1-1 Rokkodai-cho, Nada-ku, Kobe 657-8501, Japan; ‡ Graduate School of Science, Technology and Innovation, Kobe University, 1-1 Rokkodai-cho, Nada-ku, Kobe 657-8501, Japan; § Department of Biotechnology and Life Science, Graduate School of Engineering, Tokyo University of Agriculture and Technology, Koganei, Tokyo 184-8588, Japan; ∥ Strategic Planning Office, Kobe University, 1-1 Rokkodai-cho, Nada-ku, Kobe 657-8501, Japan; ⊥ Research Center for Sustainable Resource Science, RIKEN, 1-7-22 Suehiro, Tsurumi, Yokohama, Kanagawa 230-0045, Japan

## Abstract

The significance of gut microbiota in human health has gained increasing attention. Accordingly, metabolomics has been used to elucidate host–microbiota interactions. Liquid chromatography-tandem mass spectrometry (LC-MS/MS) is an ideal choice for metabolome analysis of gut microbiota due to its quantitative capabilities. However, conventional LC-MS/MS requires multiple columns, multiple mobile phases, and complex procedures to optimize conditions for each target metabolite. To address these limitations, we developed a quantitative serial LC-MS/MS method, termed the Kobe University Serial LC-MS/MS Analysis using Multiple columns with a Single mobile phase (KUSLAMS). This platform integrates two columns (PFPP and C18) and a derivatization method for seamless, high-throughput quantification of 215 metabolites, including amino acids, nucleotides, carboxylic acids, amines, and fatty acids. Reproducibility for repeated analysis was assessed using 82 intracellular gut microbiota metabolites, for which new analytical methods were developed. Among these, 64 metabolites were detected with coefficients of variation (CV) below 15%. The application of KUSLAMS to an in vitro gut microbiota culture system with and without inulin revealed differences in the concentrations of 21 intracellular and 14 extracellular metabolites. Notably, several metabolites exhibited increased intracellular and decreased extracellular concentrations, suggesting a possible link between intracellular accumulation and extracellular depletion, although this interpretation is exploratory. These results indicate that KUSLAMS allows for the simultaneous monitoring of intra- and extracellular metabolite dynamics. Together, these findings demonstrate that KUSLAMS is a robust and versatile platform for the exploration of microbiota-derived metabolites relevant to human health.

## Graphical abstract

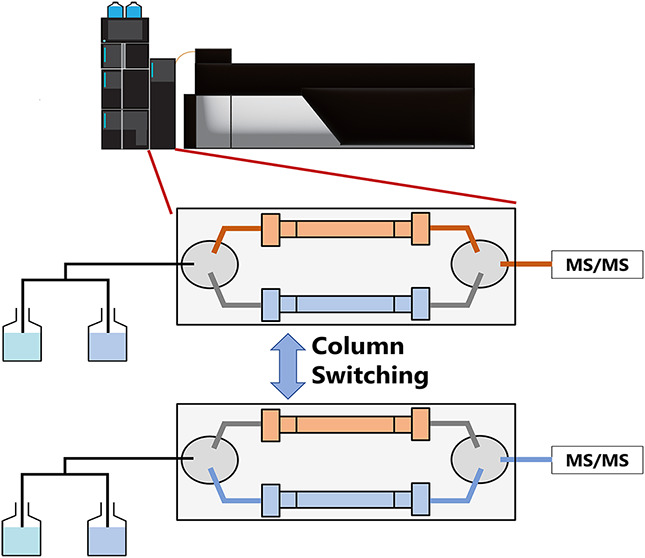

The gut microbiota comprises the entire community of symbiotic microorganisms, their collective genes and metabolites, and their surrounding environment. The human gut harbors billions of microorganisms that produce complex networks of metabolites. In recent years, it has become clear that the gut microbiota is associated with both the maintenance of human health and the development of disease.^1,2^ Accordingly, metabolomics approaches have gained traction for characterizing host–microbiota interactions. Inulin-type fructans have been shown to improve active ulcerative colitis, potentially through enhanced production of short-chain fatty acids (SCFAs) by the gut microbiota.^3^ In contrast, trimethylamine-*N*-oxide (TMAO), produced by the gut microbiota, has been identified as a causal factor in cardiovascular disease.^4^ Therefore, modulation of the gut microbiota can be leveraged to maintain health and treat disease. Strategies such as probiotic and prebiotic supplementation, as well as fecal microbiota transplantation (FMT), are currently employed for this purpose.^2,5^

However, the exact mechanisms underlying the therapeutic effects resulting from the modulation of the human gut microbiome are complex and difficult to elucidate. Therefore, the development of improved analytical methods to clarify the effects of gut microbiota modulation is essential for advancing probiotic-based approaches. Currently, metagenomics, epigenomics, transcriptomics, and metabolomics are being used to investigate the relationship between the gut microbiota and human health and disease.^6^ Among these omics approaches, metabolomics is essential because metabolites produced by the gut microbiota have been shown to exert direct effects on host physiology. For instance, microbiota-derived indole-3-acetic acid (3-IAA) has been reported to modulate chemotherapy efficacy in pancreatic cancer.^7^ Gas chromatography-mass spectrometry (GC-MS), capillary electrophoresis-mass spectrometry (CE-MS), and LC-MS/MS have been used to quantify metabolites (including SCFAs, organic acids, polyamines, and amino acids) derived from the gut microbiota.^8^ However, GC-MS and CE-MS may lack the sensitivity and coverage required for comprehensive profiling of gut microbial metabolites.^9^ Therefore, LC-MS/MS is currently preferred as a more sensitive and versatile analytical method capable of analyzing a wide variety of metabolites by combining appropriate column and mobile phase conditions.

Various analytical methods have been developed in previous reports to quantify a range of metabolites that serve as important markers for understanding gut microbiota. For instance, polyamines have been analyzed using C18 columns, and SCFAs have often been quantified after derivatization because underivatized SCFAs can show limited retention and lower ESI response in reversed-phase LC-MS.^9–12^ We have also previously developed methods to measure a wide range of primary metabolites.^13^ However, due to differences in mobile phase conditions, these analyses could not be performed consecutively on a single instrument, resulting in reduced throughput and limited practicality. Another analytical method, two-dimensional liquid chromatography-mass spectrometry (2D-LC-MS), has also been explored as an approach to cover chemically diverse metabolites by combining two different LC separations.^14^ However, in 2D-LC-MS, transferring fractions from the first separation to the second separation can broaden peaks depending on the transfer volume and solvent compatibility, which may reduce detection sensitivity for low-abundance metabolites. In addition, the choice of mobile phase conditions is constrained because the first-separation mobile phase from the first separation can be carried out in the second separation. Moreover, offline 2D-LC-MS typically requires fraction collection and reinjection, which increases the operational complexity and total analysis time. Therefore, 2D-LC-MS workflows are less practical for routine targeted quantification.

In the present study, we aimed to develop a more streamlined quantitative serial analytical method for profiling diverse metabolites relevant to gut microbiota function. A total of 215 metabolites were selected from previous studies as candidate markers of pre- and probiotic functions. These targets were curated to cover core gut microbial metabolic pathways, including short-chain fatty acid production, amino acid/indole metabolism, and bile acid transformation. In addition, the panel includes metabolites previously reported as microbiome- and disease-associated biomarkers (e.g., colorectal cancer-associated fecal metabolites), guided by prior large-cohort and microbiome-focused metabolomics studies.^15,16^ We developed a unified analytical method that employs a single mobile phase in combination with two columns (PFPP and C18) and a derivatization strategy. The PFPP column uses a pentafluorophenylpropyl stationary phase and enables separation of a wide range of metabolites based on hydrogen bonding, dipole–dipole interactions, π–π interactions, and hydrophobic interactions.^17^ The C18 column primarily facilitates separation through the hydrophobic interactions. By integrating these two columns with distinct separation mechanisms, we enabled comprehensive analysis of chemically diverse gut microbiota-derived metabolites.^18^ In KUSLAMS, the same prepared sample is injected separately onto the PFPP and C18 columns, while both columns operate by using the same mobile phase conditions through automated column switching. This design simplifies routine analysis and improves the throughput for targeted LC-MS/MS quantification. Compared with our previous method, which enabled quantification of 113 metabolites using a single PFPP separation,^13^ KUSLAMS expands metabolite coverage by serially combining PFPP and C18 separations (and an additional derivatization-based C18 run) under the same mobile phase composition through automated column switching, thereby simplifying operation and improving practical throughput.

Using intracellular metabolite extracts from gut microbiota, we evaluated the analytical system for both robustness and repeatability, resulting in favorable coefficients of variation (CVs). To this end, we used the Kobe University Human Intestinal Microbiota Model (KUHIMM), which enables high-fidelity cultivation of fecal microbiota, to conduct metabolic analyses with and without inulin as a prebiotic. Consequently, we detected metabolites that were significantly altered in the KUHIMM cultures following inulin supplementation. These findings support the conclusion that KUSLAMS is a robust, quantitative, and user-friendly platform that enables the detection of dynamic metabolic changes in the gut microbiota.

## Results and Discussion

### Construction of a Serial Analytical Method for 215 Metabolites

In this study, we developed a novel LC-MS/MS method operating in multiple reaction monitoring (MRM) mode for the quantitative analysis of 215 target metabolites. These include amino acids, nucleosides, nucleotides, carboxylic acids, amines, fatty acids, and other small molecules. The method incorporates a chemical derivatization step and integrates two distinct types of chromatographic columns (PFPP and C18) to achieve broad and complementary coverage of metabolite classes (Figure 1).

**Figure 1:**
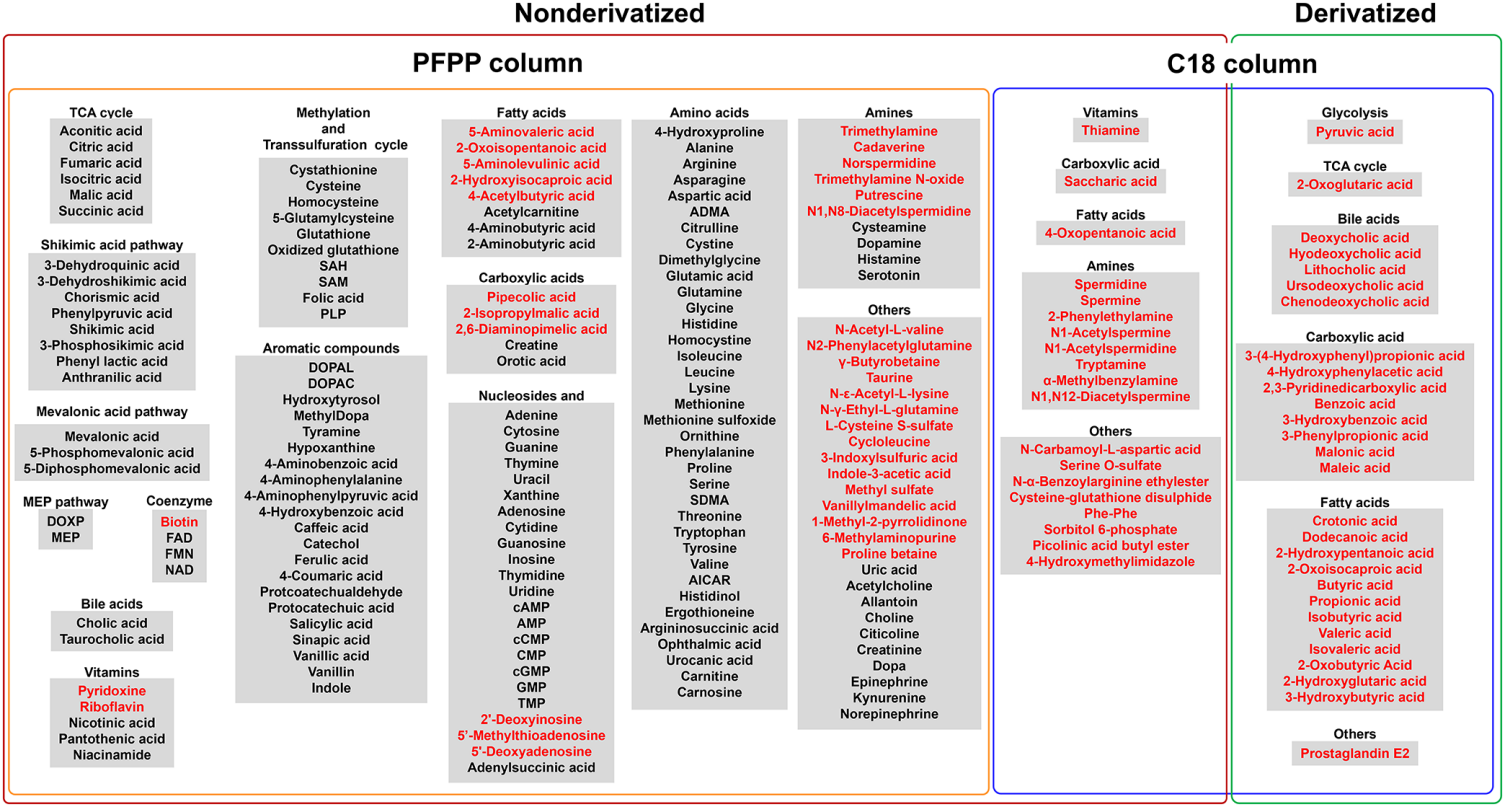
List of target metabolites. A total of 215 compounds were included, comprising 187 nonderivatized and 28 derivatized metabolites. Of the nonderivatized metabolites, 168 were analyzed using the PFPP column and 19 using the C18 column. All derivatized metabolites were analyzed using the C18 column. Metabolites newly added in this study are highlighted in red.

The two columns operate under identical mobile phase conditions (0.1% formic acid in water and 0.1% formic acid in acetonitrile), enabling a seamless serial analysis without the need to exchange columns or mobile phases. This configuration is achieved using a switching valve that changes the flow path between the PFPP and C18 columns at the batch level (Figure 2).

**Figure 2:**
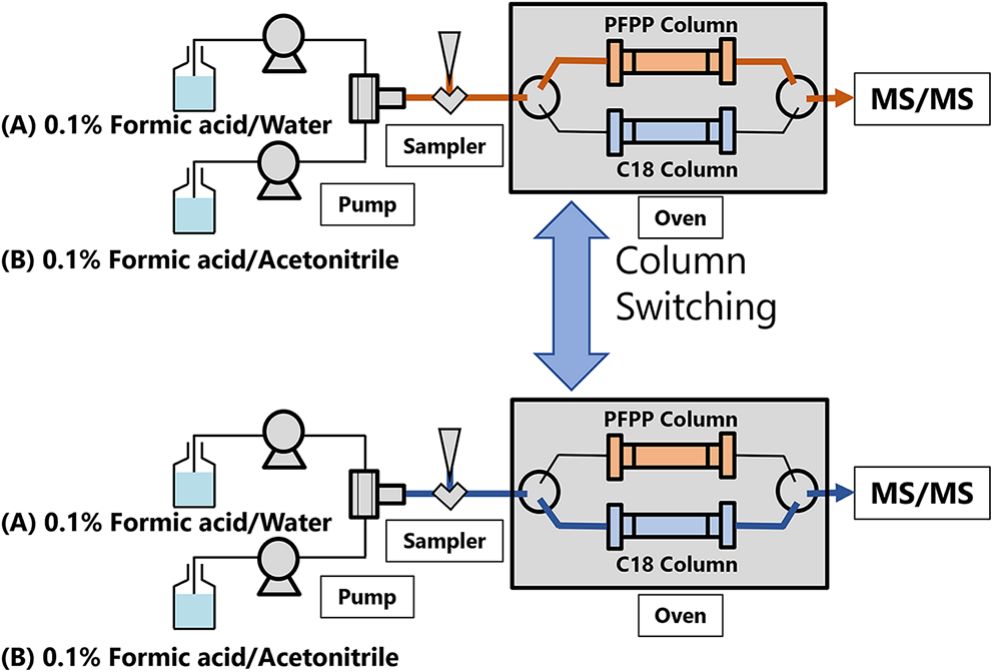
Schematic of the serial LC-MS/MS analysis system. The system is equipped with a column-switching valve and two columns (PFPP and C18) installed in a column oven. The valve is used to switch the flow path between the PFPP column (top; orange flow path) and the C18 column (bottom; blue flow path) at the batch level, enabling serial measurements under identical mobile phase conditions for both separations. The mobile phases were (A) 0.1% formic acid in water and (B) 0.1% formic acid in acetonitrile.

In this study, we analyzed 82 additional metabolites relevant to gut microbiota metabolomics, in addition to those previously covered by our established LC-MS/MS methods.^13^ These newly targeted metabolites include 35 metabolites analyzed using the PFPP column, 19 metabolites analyzed using the C18 column, and 28 metabolites analyzed through a combination of derivatization and C18 column separation (Figure 3 presents the corresponding chromatograms). Table S1 summarizes the validation parameters (e.g., MRM transitions, retention times, calibration characteristics, and sensitivity metrics). The KUSLAMS system enabled sensitive quantification across a broad calibration dynamic range; in particular, 31 of the 215 targeted metabolites (marked with asterisks in Table S1) exhibited wide dynamic ranges on the order of 10^4^–10^5^ (see the “Dynamic range” column in Table S1).

**Figure 3:**
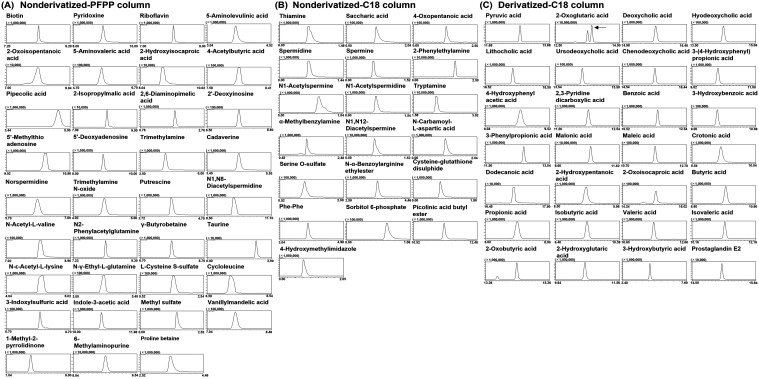
MRM chromatograms of metabolites newly added in this study. (A) Metabolites analyzed using the PFPP column. (B) Metabolites analyzed using the C18 column. (C) Metabolites analyzed using derivatization followed by C18 column separation. Standard solutions were injected at 10 μM for 2-oxoisopentanoic acid and 1 μM for the other metabolites. In panel (C), the arrow indicates the 2-oxoglutaric acid peak used for analysis. Two peaks were observed for 2-oxoglutaric acid due to isomer formation during derivatization. For each chromatogram, a cropped retention-time window around the target peak is shown; the start and end times of the displayed retention-time window are annotated in each chromatogram and are also listed in Table S2.

The sensitivity of KUSLAMS was comparable to that reported in previous studies.^9,11–13,19^ These results suggest that the serial analysis using column switching provides sensitivity and repeatability equivalent to those of independent analyses. Representative targeted workflows often rely on multiple LC methods/mobile phase conditions or two-dimensional separations to achieve broad chemical coverage, which can increase operational complexity.^13,14^ In contrast, KUSLAMS expands chemical-class coverage using a single mobile phase composition and serial PFPP/C18 separations enabled by automated column switching, improving the practicality for routine targeted quantification. These practical differences are summarized in Table S3, which compares key parameters with representative LC-MS/MS-based targeted workflows. Overall, KUSLAMS provides a practical platform for routine targeted quantification of diverse gut microbiota-derived metabolites.

### Evaluation of the Analytical System Using Biological Samples

Biological samples contain a wide variety of components, such as metabolites, proteins, and lipids, which can cause matrix effects that adversely affect detection and quantitative sensitivity.^20^ The performance of the analytical system was evaluated by examining the repeatability of peak areas for intracellular gut microbiota metabolites. These metabolites were extracted using an improved procedure based on a previously reported *Escherichia coli* extraction method.^21^ Of the 82 newly targeted metabolites, 64 were successfully detected with high repeatability (CV ≤ 15%) (Figure 4). Notably, most metabolites exhibited CVs below 10%, with 48 metabolites exhibiting CVs of less than 5%. Despite the complexity of biological matrices, KUSLAMS demonstrated excellent repeatability. These findings indicate that KUSLAMS is a robust and highly reliable analytical platform for metabolomic analyses of biological specimens. In addition, instrumental repeatability (replicate injections), interday precision, carryover, sample stability, spike recovery, and matrix effects were evaluated and are summarized in Tables S4–S9. For further context, a comparative summary of key method metrics (analyte coverage, total instrument time per sample, and validation items) versus our previous method^13^ and a representative 2D-LC × LC-MS workflow is provided in Table S3.

**Figure 4:**
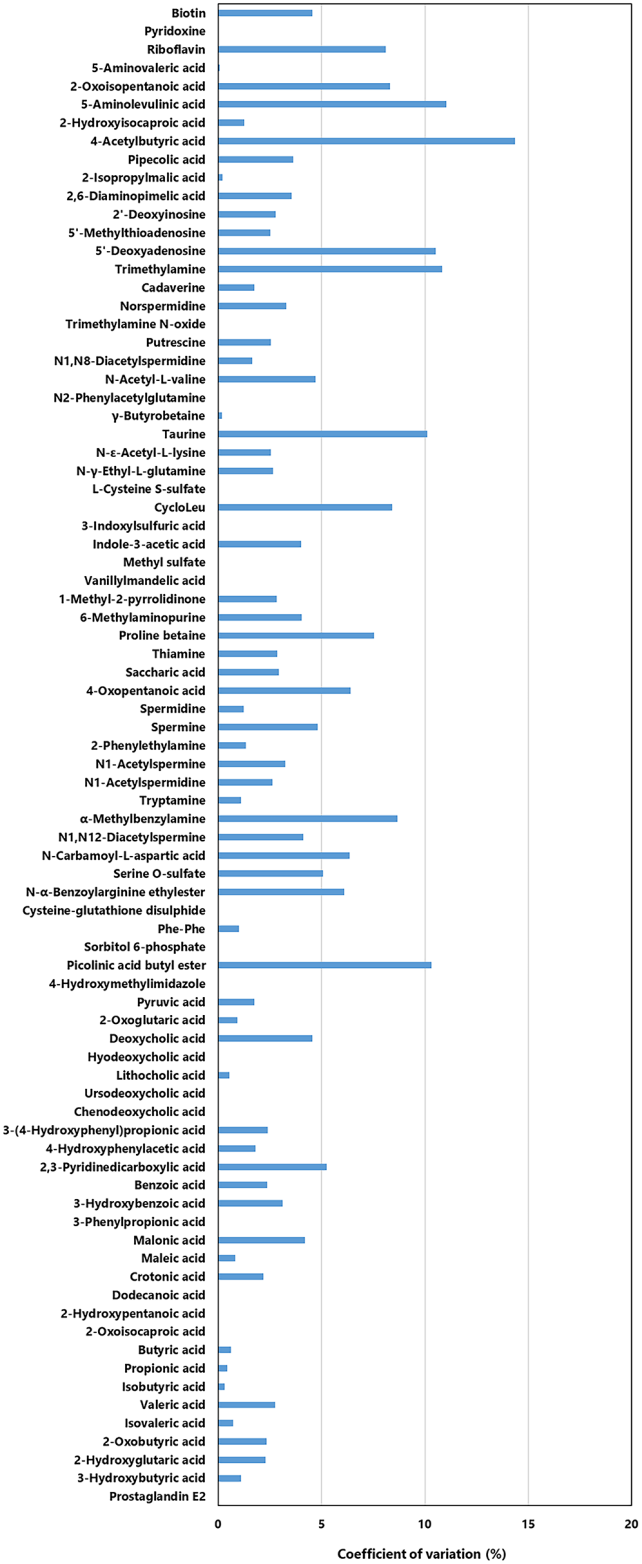
Validation of peak area repeatability using intracellular metabolite extracts from gut microbiota. Repeatability was assessed using three intraday replicates (*n* = 3). CVs were calculated from MRM peak areas for each metabolite, and the majority exhibited CV ≤ 15%.

### Human Gut Microbiota Metabolomics

Metabolomic analysis of KUHIMM cultures supplemented with inulin was performed by using the KUSLAMS platform. Prior to the metabolomic analysis, the reproducibility of inulin’s effect was confirmed using high-performance liquid chromatography (HPLC). Acetic acid, the most abundant metabolite, showed a significant increase following inulin supplementation (Figure 5).

**Figure 5:**
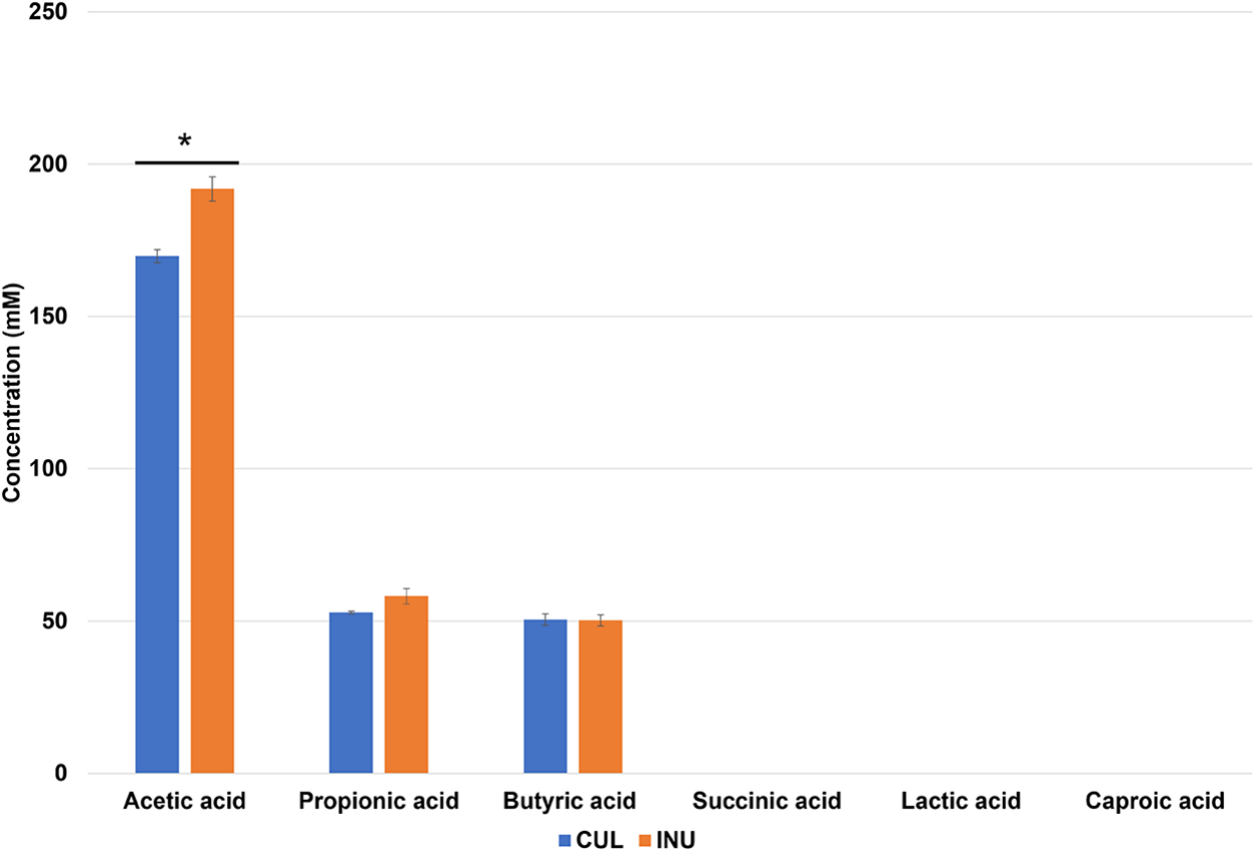
Concentration of the short-chain fatty acids (SCFAs) and organic acids in KUHIMM cultures at 72 h of incubation. Culture without inulin (CUL); culture with 0.3% inulin (INU). Acetic acid levels significantly increased with inulin supplementation. Values represent means ± SD (*n* = 3). *p < 0.05 by unpaired *t* test.

Additionally, 16S rRNA sequencing was performed. The relative abundance of *Bifidobacterium*, a known acetic acid-producing bacterium, increased by approximately 16% following inulin supplementation (Figure S1). This increase in *Bifidobacterium* abundance is consistent with previous clinical reports.^22^ A total of 159 intracellular metabolites were quantified, revealing temporal changes in their concentrations (Figure 6). These results indicate that the sensitivity of KUSLAMS is sufficient to capture the dynamic range of metabolite concentrations present in the human gut microbiota.

**Figure 6:**
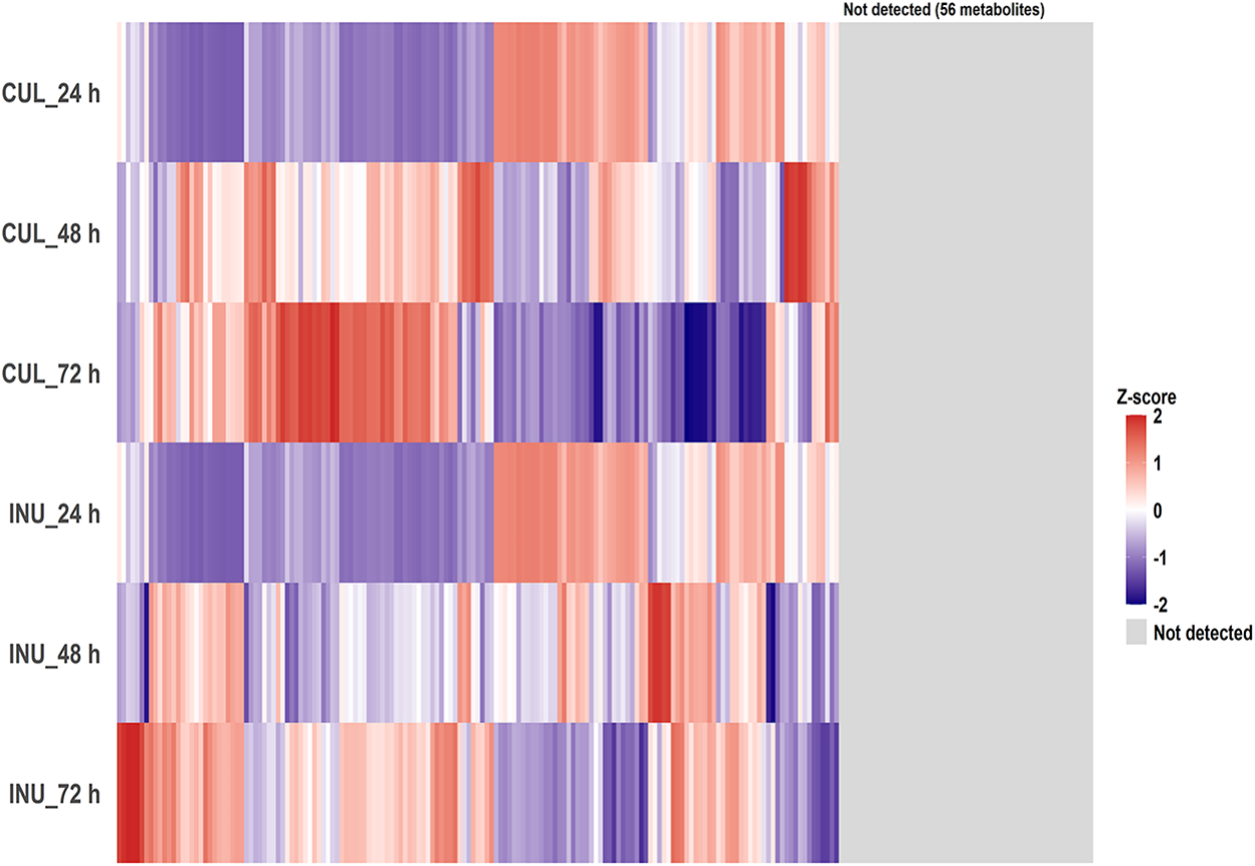
Heatmap of intracellular metabolite profiles in KUHIMM cultures with or without inulin. Culture without inulin (CUL); culture with 0.3% inulin (INU). Intracellular metabolites were extracted at 24, 48, and 72 h of incubation. Data are shown as the mean of three biological replicates (*n* = 3). Mean concentrations were log 10-transformed (log 10­(*x* + 1)) and then standardized to metabolite-specific *z*-scores across the six condition–time combinations (CUL/INU × 24, 48, and 72 h). *z*-Scores were capped at ±2 for visualization. Metabolites not detected in any sample are shown in gray. The metabolite order used in the heatmap is provided in Table S10. A total of 159 intracellular metabolites were quantified.

To obtain an overview of global metabolite profile differences, we performed principal component analysis (PCA) for both intracellular and extracellular data sets; the PCA score plots show a separation trend between CUL and INU groups (Figure S2). A total of 21 intracellular metabolites showed >2-fold changes (unadjusted *p*-values) after 72 h of incubation (Figure 7A). In parallel, we conducted extracellular metabolomic analysis and identified 14 metabolites in the culture supernatant that were altered (Figure 7B). These “significant” metabolites are based on unadjusted p-values (together with fold-change criteria). Benjamini–Hochberg false discovery rate (FDR)-adjusted *p*-values are provided in Table S11; most metabolites did not meet the FDR-adjusted significance threshold, and the findings are therefore presented as exploratory signals.

**Figure 7:**
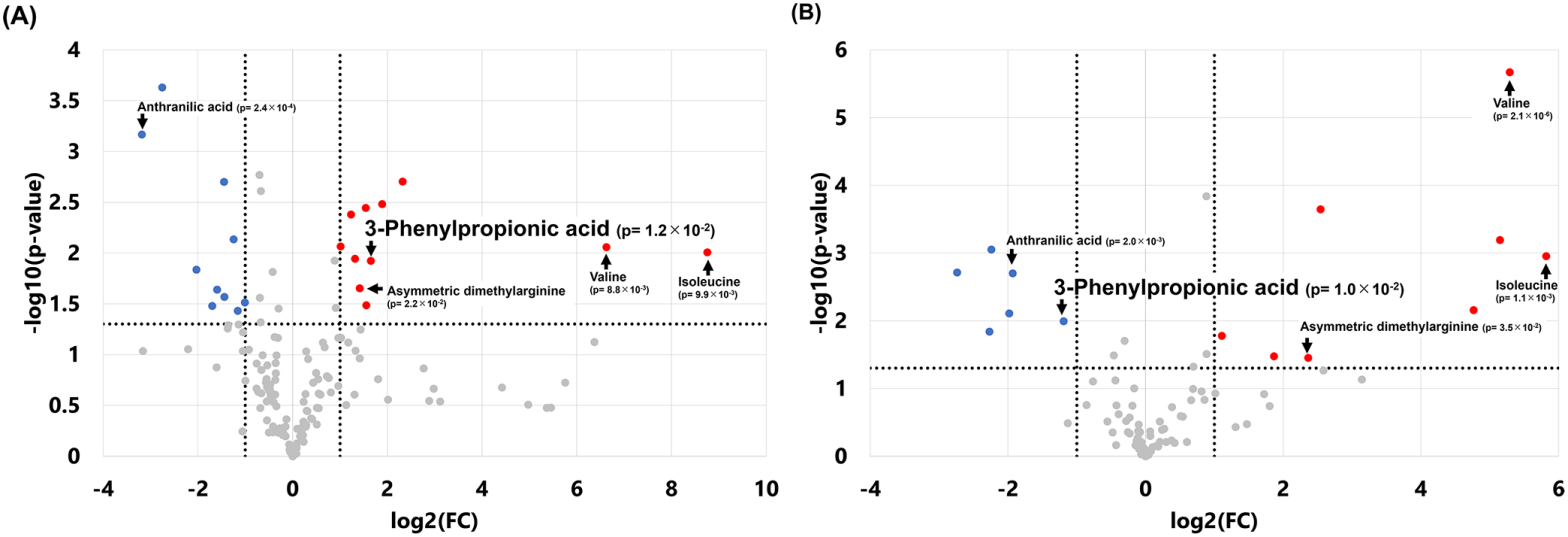
Volcano plots show metabolite changes in KUHIMM cultures after 72 h of incubation. Culture without inulin (CUL) and culture with 0.3% inulin (INU) were compared (*n* = 3 per group). (A) Intracellular metabolites. (B) Extracellular metabolites. For both panels, metabolites meeting the criteria of >2-fold change and unadjusted *p* < 0.05 are highlighted (nominal significance); 21 intracellular and 14 extracellular metabolites met these criteria. Points are colored by direction of change (red, increased; blue, decreased), and representative metabolites are annotated on the plots for readability. Benjamini–Hochberg FDR-adjusted *p*-values (together with raw *p*-values) are provided in Table S11.

A total of 5 metabolites were common to both the intra- and extracellular compartments. Notably, 3-phenylpropionic acid exhibited opposing trends with an increase in intracellular concentrations and a decrease in extracellular levels. This pattern suggests a possible link between intracellular accumulation and extracellular depletion of 3-phenylpropionic acid; however, this interpretation is exploratory and requires further validation. A previous study in *E. coli* suggested that *hcaT* encodes a 3-phenylpropionic acid (3-phenylpropionate) transporter and that genes in the adjacent *hca*/*mhp* clusters are involved in 3-phenylpropionate catabolism.^23^ While this finding provides a plausible example of microbial transport, it may not directly reflect transport or uptake mechanisms in complex community cultures such as KUHIMM. Future studies using targeted uptake assays and/or stable isotope tracing will be important to test this hypothesis. Nonetheless, by enabling the simultaneous tracking of intra- and extracellular metabolite dynamics, KUSLAMS allowed us to capture coordinated changes in the gut microbiota in response to prebiotic treatment. However, several metabolites exhibited high CVs, likely due to manual steps (e.g., operator-dependent pipetting accuracy and precision when handling organic solvents) in sample preparation and derivatization. These issues could potentially be minimized through automation.

## Conclusions

KUSLAMS enables serial quantification of diverse metabolites with an increase in throughput gained by continuously switching between PFPP and C18 under a single mobile phase. The broad metabolite coverage and high quantitative reproducibility can be explained from both LC and MS perspectives. From the LC perspective, two primary factors are implicated: (i) leveraging the distinct interaction mechanisms of the PFPP and C18 columns and (ii) using a single mobile phase containing 0.1% formic acid, which maintains acidic conditions (pH ≤ 3.0), suppresses nonspecific ion interactions, and improves separation and retention-time stability. From the MS perspective, continuous switching between PFPP and C18 while using a single mobile phase keeps the ionization conditions effectively constant, thereby improving reproducibility. Therefore, this study emphasizes the following design concept for LC-MS/MS systems: alternating column operation under a single mobile phase to achieve high reproducibility and throughput.

## Experimental Section

### Chemical and Biological Materials

Authentic standards and reagents were purchased from Nacalai Tesque, Inc. (Kyoto, Japan), Sigma-Aldrich (MO, USA), FUJIFILM Wako Pure Chemical Corporation (Osaka, Japan), Santa Cruz Biotechnology, Inc. (TX, USA), Tokyo Chemical Industry Co., Ltd. (Tokyo, Japan), Toronto Research Chemicals, Inc. (North York, Canada), Peptide Institute, Inc. (Osaka, Japan), Combi Blocks Inc. (CA, USA), BLD Pharmatech Ltd. (Shanghai, China), Cayman Chemical Company (MI, USA), AstaTech Inc. (PA, USA), and Matrix Scientific (SC, USA). For standard curves, standards were diluted to the following concentrations: 0.0001, 0.001, 0.005, 0.01, 0.05, 0.1, 0.5, 1, 5, and 10 μM. Additionally, as an internal standard, the PFPP column and C18 column standard solution contained 1 μM *d*-camphorsulfonic acid, and the derivatization standard solution contained 10 μM 2-ethylbutyric acid. For nonderivatized analyses (PFPP and C18 methods), *d*-camphorsulfonic acid was used as the internal standard. For derivatized analyses, 2-ethylbutyric acid was used as the internal standard. Isotopically labeled internal standards were not used in this study. This internal standard strategy may not be fully compensatory because metabolite-specific differences in ionization and derivatization efficiencies may not be adequately accounted for by the internal standard.

A fresh fecal sample was obtained from a healthy Japanese volunteer. The inclusion criteria were as follows: Japanese ancestry, no pre-existing illness (according to patient interviews), nonsmoker, and no antibiotic treatment for at least 6 months prior to sampling. Immediately after collection, the fecal sample was stored under anaerobic conditions and used within 48 h. This study was conducted based on the principles of the Declaration of Helsinki, and a participant provided written informed consent. The study design was approved by the Institutional Ethics Review Board (research code 151660_rn-34542, approval date Jan 30, 2023).

### Derivatization

The derivatization method was modified from a previous report.^19^ The standard solution was derivatized as follows: 2.5 μL of the solution to be derivatized was mixed with 7.5 μL of 267.2 μM 2-ethylbutyric acid (internal standard). Then, 5 μL of the mixture was transferred to a new tube and mixed with 5 μL of 175 mM 3-nitrophenylhydrazine hydrochloride in 75% methanol, 5 μL of 105 mM 1-(3-(dimethylamino)­propyl)-3-ethylcarbodiimide hydrochloride in 75% methanol, and 5 μL of 2.5% pyridine in 75% methanol. After shaking for 1 h at room temperature, the reaction was quenched with 80 μL of 0.5% formic acid in 75% methanol. Intracellular metabolites from the gut microbiome were derivatized as follows. The dried sample was dissolved in 6.25 μL of 75% ethanol and then diluted 40-fold. Subsequently, 2.5 μL was transferred to a new tube, and 2.5 μL of 400.8 μM 2-ethylbutyric acid (internal standard) was added. The procedure thereafter was the same as that for the standard solution. Extracellular metabolites were diluted 25-fold and 250-fold and derivatized in the same manner as the standard solution.

### LC-MS/MS Analysis

The LC-MS/MS system consisted of a Nexera X3 high-performance liquid chromatography system equipped with an LC-40B X3 solvent delivery module, an SIL-40C X3 autosampler, a CTO-40S column oven with FCV-0206 and FCV-0206H3 column-switching valves, an SCL-40 system controller, and an LCMS-8060NX triple quadrupole mass spectrometer (Shimadzu Corporation, Kyoto, Japan).

Each sample was analyzed by three injections in a fixed order: (i) a nonderivatized sample injected for the PFPP column method, (ii) the same nonderivatized sample injected for the C18 column method, and (iii) a separately prepared derivatized sample injected for the C18 column method (derivatization described in the Derivatization section). Thus, the nonderivatized sample was injected twice from the same vial (PFPP and C18), and the derivatized sample was analyzed as a separate injection. The switching valve was operated at the batch level: after completing the PFPP runs, the valve was switched once to the C18 configuration, and the C18 analyses were performed in the order of nonderivatized samples followed by derivatized samples without further valve switching.

LC-MS/MS analysis was performed under the following MS conditions and two LC methods. The MS conditions were as follows: nebulizer flow, 3.0 L/min; drying gas flow, 10.0 L/min; heating gas flow, 10.0 L/min; DL temperature, 250 °C; interface temperature, 270 °C; and block heater temperature, 400 °C. Other MS parameters were determined by autotuning. The first LC method, which we call the PFPP column method, was performed on a Discovery HS F5-3 column (2.1 mm ID, 150 mm length, 3 μm particle size; Merck KGaA, Darmstadt, Germany). Mobile phase A was water containing 0.1% (v/v) formic acid, and mobile phase B was acetonitrile containing 0.1% (v/v) formic acid. The flow rate was 0.25 mL/min, the injection volume was 3 μL, and the column temperature was maintained at 40 °C. The gradient program was 0% B (0–2 min), 25% B (2–5 min), 35% B (5–11 min), 95% B (11–15 min), and 95% B (15–20 min),followed by 0% B (20.1–30 min).^13^

The second LC method, which we call the C18 column method (used for both nonderivatized and derivatized samples), was performed on a Mastro2 C18 column (2.0 mm ID, 150 mm length, 3 μm particle size, Shimadzu GLC, Tokyo, Japan) with the same mobile phases (A and B). The flow rate was 0.35 mL/min, the injection volume was 3 μL, and the column temperature was maintained at 40 °C. The gradient program was 16% B (0 min), 25% B (0–6) min, 40% B (6–9 min), 95% B (9–17 min), and 95% B (17–20 min), followed by 16% B (20.1–23 min). MRM conditions were set automatically with the aid of LabSolutions (Shimadzu Corporation, Kyoto, Japan) using a standard solution (1 μM). The MRM parameters (including ionization mode, adduct ion, and fragmentation energy) are provided in Table S12. Acquired data were peak picked with Cascade (version 1.1.8.4989, Reifycs, Tokyo, Japan). A part of the validation parameters was obtained in our previous study.^13^ These metabolites were indicated with a dagger icon in Table S1.

Metabolite concentrations were calculated using calibration curves based on the peak area ratio of each analyte to the corresponding internal standard (*d*-camphorsulfonic acid for nonderivatized runs and 2-ethylbutyric acid for derivatized runs).

### Cultivation of Fecal Samples in a Jar Fermenter (KUHIMM)

A Bio Jr. 8 fermenter (ABLE, Tokyo, Japan) comprising eight parallel and independent anaerobic culturing vessels, named as KUHIMM, was used as described previously.^24,25^ The fermenter system was used for the fecal sample cultivation of one volunteer. Briefly, 0.5 g of fecal samples was suspended in 2 mL of PBS buffer (Nacalai Tesque, Kyoto, Japan). Each vessel containing 100 mL of Gifu anaerobic medium (GAM; Shimadzu Diagnostics Corporation, Kyoto, Japan) was inoculated with either 2000 μL of fecal suspension alone or with respective amounts of additives and then cultivated anaerobically at 37 °C. The culture broth was stirred at 300 rpm and continuously purged with an anaerobic gas mixture (N_2_/CO_2_ = 80:20) to maintain anaerobic conditions. After 24 h of cultivation, inulin from chicory (Merck KGaA, Darmstadt, Germany) was added into the culturing medium, respectively, at 0 or 0.3%. After 24 and 48 h of further cultivation (48 and 72 h from culture start), the culture broths were collected and used for subsequent analyses.

### Extracellular Metabolites Preparation (for KUSLAMS and HPLC)

Samples were centrifuged at 10,000*g* at 4 °C for 3 min, after which 200 μL of the supernatant was diluted 5 times with ultrapure water. The diluted sample solution was filtered through a 0.22 μm filter (Advantec, Inc., Tokyo, Japan). The same prepared extracellular metabolites were used for both KUSLAMS and HPLC analyses (described below).

### HPLC Analysis of SCFAs and Organic Acids as a Culture-Quality Check

As a culture-quality check to verify that the inulin intervention produced the expected response before KUSLAMS measurements, SCFAs in the prepared supernatants were measured by HPLC. Acetic acid, propionic acid, butyric acid, succinic acid, lactic acid, and caproic acid were quantified following a previously reported HPLC procedure.^28^ Briefly, SCFAs were analyzed using an HPLC system (Shimadzu Corporation, Kyoto, Japan) equipped with a refractive-index detector (RID-10A, Shimadzu Corporation, Kyoto, Japan) and an Aminex HPX-87H column (Bio-Rad Laboratories, CA, USA). The column was maintained at 65 °C and eluted with 5 mM H_2_SO_4_ at a flow rate of 0.6 mL/min.

### DNA Extraction and Sequencing of 16S rRNA Genes

Extraction of microbial genomic DNA and sequencing of 16S rRNA genes were performed according to previous reports.^24,26–28^

### Intracellular Metabolites Preparation Using Cold Ethanol Quenching and Heat Ethanol Extraction

A previously reported method was modified to optimize for the gut microbiota, including cold ethanol quenching and hot ethanol extraction.^21^ Two milliliters of culture medium containing 4–7 mg of gut microbiota was collected and immediately mixed with an equal volume of a quenching solution of 40% ethanol and 60% PBS, precooled at −30 °C. Cells were collected by centrifugation at −9 °C and 4000*g* for 10 min. Then, the cells were suspended in 720 μL of ethanol and 30 μL of 8.58 μM *d*-camphorsulfonic acid (internal standard) and heated at 70 °C for 15 min. After heating, the sample was cooled on ice for 2 min, and then 750 μL of pure water was added. The extraction solution was centrifuged at −9 °C and 3940*g* for 5 min. Then, 450 μL of the supernatant was filtered by using a 3-kDa cutoff filter (Amicon Ultra, Merck Millipore, MA, USA). 300 μL of the filtrate was dried under vacuum and stored at −80 °C until analysis. For analysis of nonderivatized metabolites, the dried sample was dissolved in 50 μL of pure water before analysis. Derivatized sample preparation is described in the Derivatization section.

### Statistical Analysis

Metabolite concentrations were calculated by using the internal standard method. For intracellular data, concentrations were normalized to the dry-cell weight. Dry-cell weight for each sample was estimated from optical density (OD), and each sample was normalized using its corresponding dry-cell weight.^13^ PCA was performed using RStudio (version 2025.09.2 + 418, Posit PBC, MA, USA). The volcano plot and false discovery rate (FDR) control (Benjamini–Hochberg) were performed using Excel.

## Supplementary Material



## Data Availability

The data underlying this study are not publicly available because the raw LC-MS/MS data include analytical parameters for additional, currently unpublished metabolites. The data are available from the corresponding author upon reasonable request. Processed quantification tables for intracellular and extracellular metabolites are provided in the Supporting Information (Table S13).

## Notes

Supplementary methods; additional figures (16S rRNA sequencing and multivariate/statistical plots); supplementary tables, including MRM/retention information, validation metrics, statistical summaries (FDR-adjusted *p*-values), and processed quantification tables for intra- and extracellular metabolites (PDF)
