# Supplementary material for: Development of a Quantitative Serial LC-MS/MS Method for Gut Microbiota Metabolomics
Source: ACS Omega. 2026 Mar 16;11(12):19431–9. doi: 10.1021/acsomega.5c12997 (PMC13044636; doi:10.1021/acsomega.5c12997)
Supplement: Supplementary file 1 [file ao5c12997_si_001.pdf]

Supporting Information

Development of a Quantitative Serial LC-MS/MS Method for Gut Microbiota Metabolomics

Takanobu Yoshida[a], Tomoya Shintani[b], Daisuke Sasaki[b], Christopher J. Vavricka[c], Yasushi Matsuki[d], Akihiko Kondo[a,b,e], Tomohisa Hasunuma[a,b,e]*

[a] Engineering Biology Research Center, Kobe University, 1-1 Rokkodai-cho, Nada-ku, Kobe 657-8501, Japan

[b] Graduate School of Science, Technology and Innovation, Kobe University, 1-1 Rokkodai-cho, Nada-ku, Kobe 657-8501, Japan

[c] Department of Biotechnology and Life Science, Graduate School of Engineering, Tokyo University of Agriculture and Technology, Koganei, Tokyo 184-8588, Japan

[d] Strategic Planning Office, Kobe University, 1-1 Rokkodai-cho, Nada-ku, Kobe 657-8501, Japan

[e] Research Center for Sustainable Resource Science, RIKEN, 1-7-22 Suehiro, Tsurumi, Yokohama, Kanagawa 230-0045, Japan

* hasunuma@port.kobe-u.ac.jp.

List of Contents

Supplementary methods

Figure S1. Bacterial composition in KUHIMM cultures after 72 hours of fermentation.

Figure S2. PCA scores plots of metabolite profiles in KUHIMM cultures with (INU) or without (CUL) inulin at 72 h.

Figure S3. Completely identical chromatograms to those shown in Figure 3(C).

Table S1. Analytical validation for the determination of 215 metabolites.

Table S2. Displayed retention-time windows for the extracted ion chromatograms in Figure 3.

Table S3. Comparison of KUSLAMS with our previous PFPP-based method and a representative 2D-LC×LC–MS workflow, including analyte coverage, total instrument time, and validation items.

Table S4. Instrumental validation of peak area repeatability using intracellular metabolite extracts from gut microbiota.

Table S5. Evaluation of carryover after injection of a high-concentration standard mix.

Table S6. Inter-day validation of repeatability using intracellular metabolite extracts from gut microbiota.

Table S7. Evaluation of sample stability under three conditions (autosampler, bench-top, and freeze–thaw).

Table S8. Spike–recovery validation using intracellular metabolite extracts from gut microbiota.

Table S9. Matrix effect evaluation using slope comparison.

Table S10. Metabolite order used in Figure 6 (heatmap) and detection status.

Table S11. Raw p-values and Benjamini–Hochberg FDR-adjusted p-values for differential metabolites (Figure 7).

Table S12. MRM parameters for targeted metabolites.

Table S13. Processed metabolite concentration values (intracellular and extracellular) for the KUHIMM experiments reported in this study.

Supplementary methods

Sample preparation for validation experiments reported in the Supporting Information

The intracellular metabolite extracts used for the Supporting Information validation experiments were prepared from spare aliquots of the extracts generated under the same sample preparation procedure as the main study. For each time point and condition, three replicate extracts were prepared; two were used for the main experiments, and the remaining aliquot was retained as a reserve. These reserve dried extracts were dissolved in 300 µL of 75% ethanol, pooled into a single composite sample, and then re-aliquoted (300 µL each) into multiple tubes and dried under vacuum. The resulting extracts were used for subsequent validation tests (instrumental repeatability, inter-day precision, stability, spike–recovery, and matrix-effect assessments).

Carryover evaluation

Carryover was evaluated by injecting a water blank immediately after the highest-concentration standard mix (10 µM). For each metabolite, carryover (%) was calculated as the ratio of the MRM peak area in the blank injection to that in the preceding standard mix injection (blank/standard × 100).

Spike–recovery

Spike–recovery was evaluated using intracellular metabolite extracts by spiking a standard mixture at three concentration levels: 0.01 µM (low), 0.1 µM (mid), and 1 µM (high). Extracts were analyzed either undiluted or after dilution as needed to obtain quantifiable signals within the measurement range. Recovery (%) was calculated as (spiked sample − unspiked sample)/standard solution × 100 using MRM peak areas. Metabolites not detected in the unspiked sample were reported as "Not detected," and recovery was not calculated for those metabolites.

Matrix effect

Matrix effect (%) was calculated as (slope in spiked extract / slope in standard solution) × 100 using the

same spiked extract samples prepared for the spike–recovery experiment (0.01, 0.1, and 1 μM).

Figure S1. Bacterial composition in KUHIMM cultures after 72 hours of fermentation. Culture without inulin (CUL); culture with 0.3% inulin (INU). Relative abundance of *Bifidobacterium* was 3.55% (CUL) and increased to 4.11% (INU) indicating an approximate 16% increase in response to inulin supplementation.

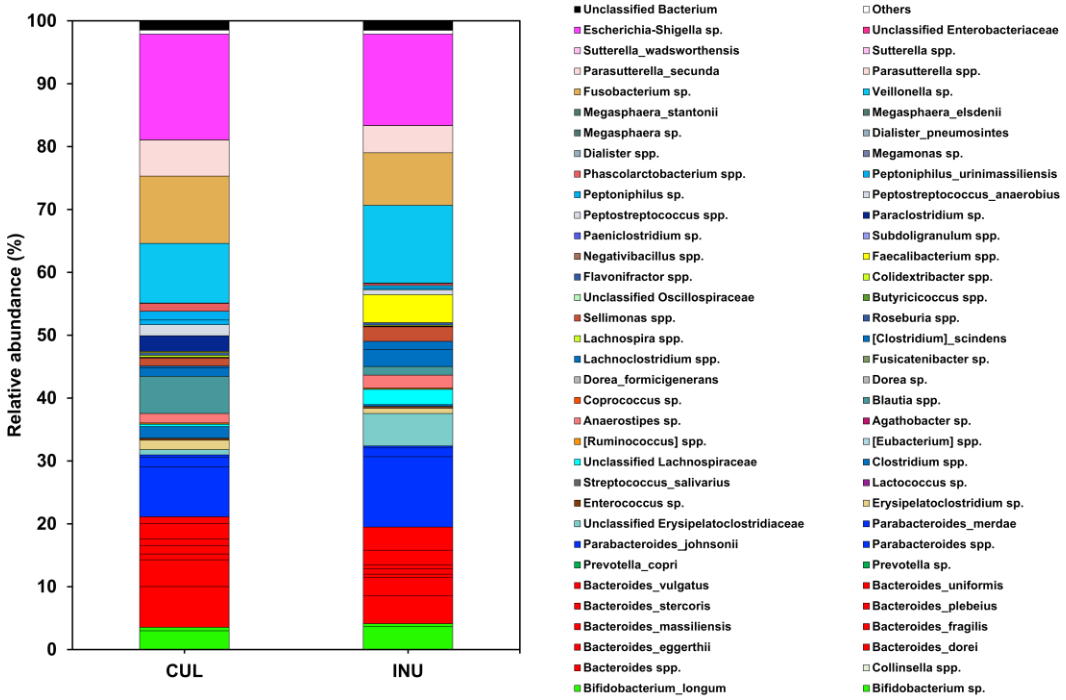

Figure S2. PCA scores plots of metabolite profiles in KUHIMM cultures with (INU) or without (CUL) inulin at 72 h. Culture without inulin (CUL); culture with 0.3% inulin (INU). (A) Intracellular metabolites. (B) Extracellular metabolites. PCA was performed separately for each dataset using quantified metabolite concentrations. Each point represents one biological replicate (n = 3 per group). Metabolites with any missing values were excluded prior to PCA. Data were log10-transformed ($log10(x+1)$), mean-centered, and autoscaled before PCA. The percentage of variance explained by PC1 and PC2 is indicated on the axes.

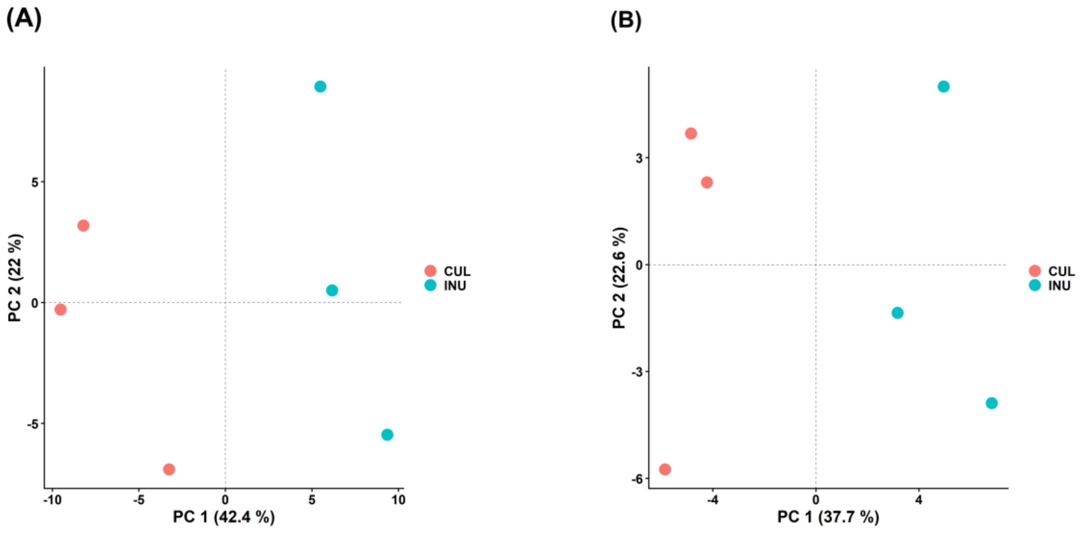

Figure S3. Completely identical chromatograms to those shown in Figure 3(C). Metabolites analyzed using derivatization followed by C18 column separation. Standard solutions were injected at 10 μM for 2-oxoisopentanoic acid and 1 μM for the other metabolites. The arrow indicates the 2-oxoglutaric acid peak used for analysis. Two peaks were observed for 2-oxoglutaric acid due to isomer formation during derivatization.

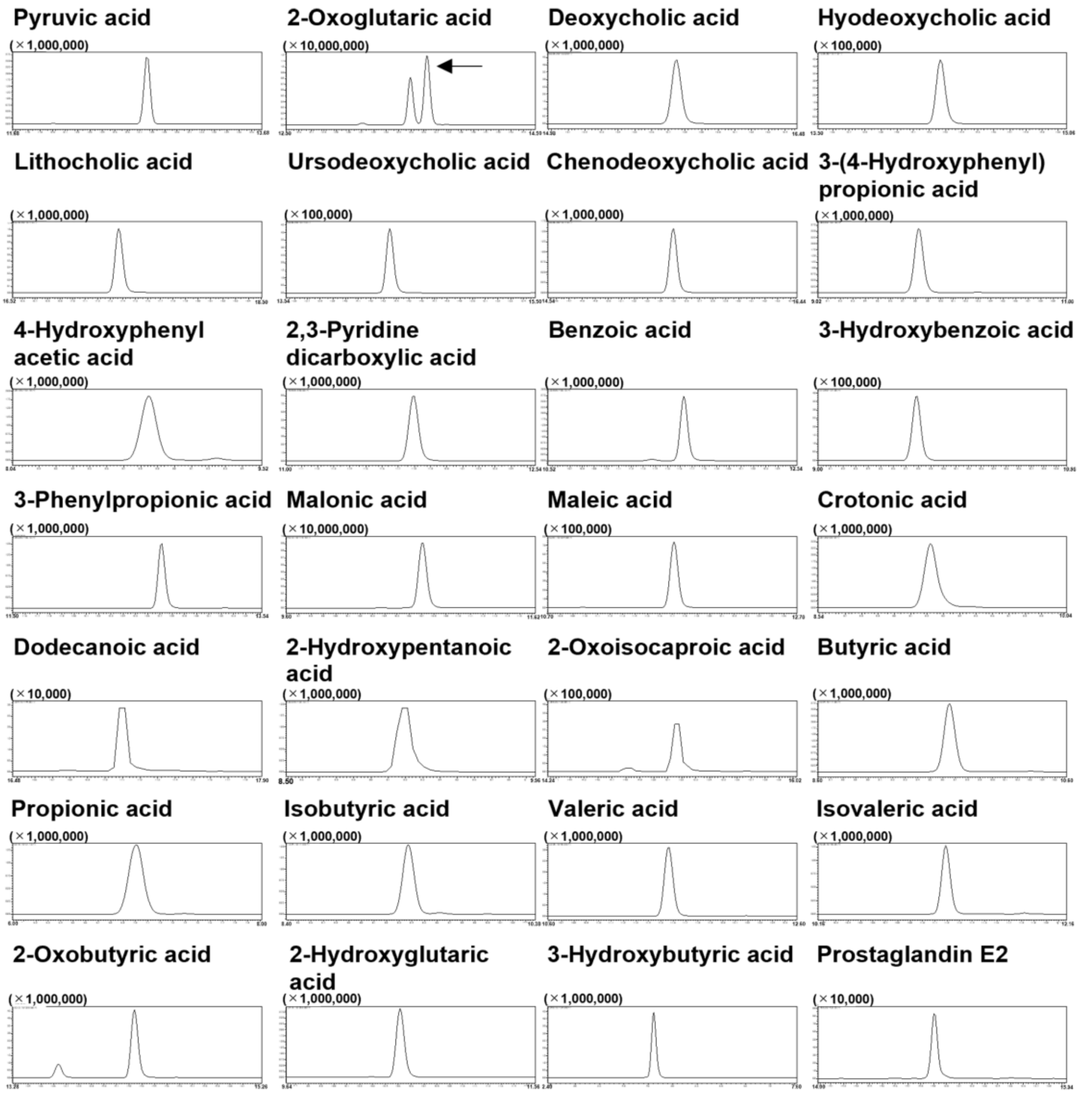

Table S1. Analytical validation for the determination of 215 metabolites. The table summarizes retention times, calibration ranges, and other performance metrics for each metabolite. Metabolites with a wide dynamic range (approximately 10^4–10^5) are indicated with an asterisk (*). Metabolites marked with “†” indicate that validation parameters were adopted from our previous report without revalidation.[13] Limit of detection was defined as the lowest tested calibration level at which the analyte peak was detectable at the expected retention time and MRM transition. Limit of quantitation was defined as the lowest calibration level included in the quantitative calibration range.

Metabolites	SMILES	MRM transition	Retention time (min)	Quantitative linear range (μM)	R^2	Dynamic range	Limit of quantitation (μM)	Limit of detection (μM)
Aconitic acid †	C(C(=CC(=O)O)C(=O)O)C(=O)O	172.90>85.05	3.80	0.005-10	0.9998	2.0×10^3	0.005	0.005
Citric acid †	C(C(=O)O)C(CC(=O)O)(C(=O)O)O	191.20>111.10	3.00	0.005-10	0.9992	2.0×10^3	0.005	0.005
Fumaric acid *†	C(=C/C(=O)O)\\C(=O)O	115.00>71.10	4.50	0.001-10	0.9988	1.0×10^4	0.001	0.001
Isocitric acid †	C(C(C(C(=O)O)O)C(=O)O)C(=O)O	191.20>111.10	2.16	0.005-10	0.9998	2.0×10^3	0.005	0.005
Malic acid †	C(C(C(=O)O)O)C(=O)O	133.10>114.95	2.22	0.01-10	0.9997	1.0×10^3	0.01	0.01
Succinic acid †	C(CC(=O)O)C(=O)O	117.30>73.00	3.91	0.005-10	0.9998	2.0×10^3	0.005	0.005
3-Dehydroquinic acid †	C1[C@H]([C@@H](C(=O)C[C@]1(C(=O)O)O)O)O	189.35>171.30	1.83	0.01-10	0.9991	1.0×10^3	0.01	0.005
3-Dehydroshikimic acid †	C1[C@H]([C@@H](C(=O)C=C1C(=O)O)O)O	171.45>109.05	2.71	0.05-10	0.9989	2.0×10^2	0.05	0.005

Table S1 (continued)

Metabolites	SMILES	MRM transition	Retention time (min)	Quantitative linear range (μM)	R^2	Dynamic range	Limit of quantitation (μM)	Limit of detection (μM)
Chorismic acid †	C=C(C(=O)O)O[C@@H]1C=C(C=C[C@H]1O)C(=O)O	225.00>137.10	8.00	0.05-10	0.9999	2.0×10^2	0.05	0.050
Shikimic acid †	C1[C@H]([C@@H]([C@@H](C=C1C(=O)O)O)O)O	173.20>93.00	2.27	0.005-10	0.9999	2.0×10^3	0.005	0.001
Phenylpyruvic acid †	C1=CC=C(C=C1)CC(=O)C(=O)O	163.15＞91.10	9.74	0.005-10	0.9994	2.0×10^3	0.005	0.001
3-Phosphoshikimic acid †	C1[C@H]([C@@H]([C@@H](C=C1C(=O)O)OP(=O)(O)O)O)O	253.20＞96.95	1.41	0.1-10	0.9999	1.0×10^2	0.1	0.050
Phenyllactic acid †	CC(C1=CC=CC=C1)(C(=O)O)O	165.15>147.30	8.60	0.01-10	0.9999	1.0×10^3	0.01	0.010
Anthranilic acid †	C1=CC=C(C(=C1)C(=O)O)N	138.00>119.95	8.70	0.005-10	0.9948	2.0×10^3	0.005	0.005
Mevalonic acid †	CC(CCO)(CC(=O)O)O	147.40＞59.10	4.49	0.01-10	0.9999	1.0×10^3	0.01	0.010
5-Phosphomevalonic acid †	CC(CCOP(=O)(O)O)(CC(=O)O)O	227.25＞97.00	1.87	0.005-10	0.9992	2.0×10^3	0.005	0.001
5-Diphosphomevalonic acid †	C[C@@](CCOP(=O)(O)OP(=O)(O)O)(CC(=O)O)O	306.90>79.05	1.30	0.5-10	0.9948	2.0×10	0.5	0.500
1-Deoxy-D-xylulose 5-phosphoric acid †	CC(=O)[C@H]([C@@H](COP(=O)(O)O)O)O	213.30＞97.00	1.49	0.005-10	0.9936	2.0×10^3	0.005	0.001
2-C-Methyl-D-erythritol 4-phosphoric acid †	C[C@](CO)([C@@H](COP(=O)(O)O)O)O	215.30＞97.00	1.38	0.01-1	0.9981	1.0×10^2	0.01	0.005
Biotin	C1[C@H]2[C@@H]([C@@H](S1)CCCCC(=O)O)NC(=O)N2	244.90>227.00	8.20	0.05-10	0.9989	2.0×10^2	0.05	0.005

Table S1 (continued)

Metabolites	SMILES	MRM transition	Retention time (min)	Quantitative linear range (μM)	R^2	Dynamic range	Limit of quantitation (μM)	Limit of detection (μM)
Flavin adenine dinucleotide *†	CC1=CC2=C(C=C1C)N(C3=NC(=O)NC(=O)C3=N2)C[C@@H]([C@@H]([C@@H](COP(=O)(O)OP(=O)(O)OC[C@@H]4[C@H]([C@H]([C@@H](O4)N5C=NC6=C(N=CN=C65)N)O)O)O)O)O	786.15＞136.10	7.64	0.0001-10	0.9951	1.0×10^5	0.0001	0.0001
Flavin mononucleotide †	CC1=CC2=C(C=C1C)N(C3=NC(=O)NC(=O)C3=N2)C[C@@H]([C@@H]([C@@H](COP(=O)(O)O)O)O)O	455.00＞78.90	7.70	0.005-10	0.9999	2.0×10^3	0.005	0.001
Nicotinamide adenine dinucleotide †	C1=CC(=C[N+](=C1)[C@H]2[C@@H]([C@@H]([C@H](O2)COP(=O)([O-])OP(=O)(O)OC[C@@H]3[C@H]([C@H]([C@@H](O3)N4C=NC5=C(N=CN=C54)N)O)O)O)O)C(=O)N	663.10＞541.05	2.76	0.005-10	0.9990	2.0×10^3	0.005	0.005
Cholic acid †	C[C@H](CCC(=O)O)[C@H]1CC[C@@H]2[C@@]1([C@H](C[C@H]3[C@H]2[C@@H](C[C@H]4[C@@]3(CC[C@H](C4)O)C)O)O)C	407.20＞345.25	14.75	0.1-10	0.9904	1.0×10^2	0.1	0.050
Taurocholic acid †	C[C@H](CCC(=O)NCCS(=O)(=O)O)[C@H]1CC[C@@H]2[C@@]1([C@H](C[C@H]3[C@H]2[C@@H](C[C@H]4[C@@]3(CC[C@H](C4)O)C)O)O)C	514.20＞107.10	8.83	0.01-10	0.9997	1.0×10^3	0.01	0.005
Pyridoxine *	CC1=NC=C(C(=C1O)CO)CO	170.00>152.00	8.90	0.0001-5	0.9928	5.0×10^4	0.0001	0.0001
Riboflavin	CC1=CC2=C(C=C1C)N(C3=NC(=O)NC(=O)C3=N2)C[C@@H]([C@@H]([C@@H](CO)O)O)O	376.90>243.05	8.00	0.005-10	0.9971	2.0×10^3	0.005	0.001
Nicotinic acid †	C1=CC(=CN=C1)C(=O)O	124.05＞80.05	3.92	0.005-10	0.9977	2.0×10^3	0.005	0.001
Pantothenic acid *†	CC(C)(CO)[C@H](C(=O)NCCC(=O)O)O	220.10＞90.15	7.77	0.0001-5	0.9973	5.0×10^4	0.0001	0.0001
Nicotinamide *†	C1=CC(=CN=C1)C(=O)N	123.10＞80.05	4.93	0.0001-10	0.9918	1.0×10^5	0.0001	0.0001
Cystathionine †	C(CSCC(C(=O)O)N)C(C(=O)O)N	223.00＞88.05	1.78	0.005-10	0.9997	2.0×10^3	0.005	0.005
Cysteine †	C([C@@H](C(=O)O)N)S	122.00＞76.05	2.01	0.005-10	0.9975	2.0×10^3	0.005	0.005

Table S1 (continued)

Metabolites	SMILES	MRM transition	Retention time (min)	Quantitative linear range (μM)	R^2	Dynamic range	Limit of quantitation (μM)	Limit of detection (μM)
Homocysteine †	C(CS)[C@@H](C(=O)O)N	136.00＞90.10	2.72	0.005-10	0.9997	2.0×10^3	0.005	0.001
5-Glutamylcysteine †	C(CC(=O)N[C@@H](CS)C(=O)O)[C@@H](C(=O)O)N	251.10＞84.10	3.76	0.005-10	0.9938	2.0×10^3	0.005	0.001
Glutathione *	C(CC(=O)N[C@@H](CS)C(=O)NCC(=O)O)[C@@H](C(=O)O)N	308.00＞179.10	4.00	0.001-10	0.9999	1.0×10^4	0.001	0.001
Oxidized glutathione *†	C(CC(=O)N[C@@H](CSSC[C@@H](C(=O)NCC(=O)O)NC(=O)CC[C@@H](C(=O)O)N)C(=O)NCC(=O)O)[C@@H](C(=O)O)N	611.10＞306.00	7.59	0.001-10	1.0000	1.0×10^4	0.001	0.0001
S-Adenosylhomocysteine †	C1=NC(=C2C(=N1)N(C=N2)[C@H]3[C@@H]([C@@H]([C@H](O3)CSCC[C @@H](C(=O)O)N)O)O)N	385.10＞134.00	8.65	0.005-10	0.9946	2.0×10^3	0.005	0.0001
S-Adenosylmethionine *†	C[S+](CC[C@@H](C(=O)[O-])N)C[C@@H]1[C@H]([C@H]([C@@H](O1)N2C=NC3=C(N=CN=C32)N)O)O	399.10＞250.05	7.21	0.0001-10	0.9935	1.0×10^5	0.0001	0.0001
Folic acid *	C1=CC(=CC=C1C(=O)N[C@@H](CCC(=O)O)C(=O)O)NCC2=CN=C3C(=N2) C(=O)NC(=N3)N	440.10>311.10	7.40	0.001-10	0.9992	1.0×10^4	0.001	0.001
Pyridoxal 5'-phosphoric acid	CC1=NC=C(C(=C1O)C=O)COP(=O)(O)O	248.00>150.20	2.70	0.05-10	0.9984	2.0×10^2	0.05	0.005
3,4-Dihydroxyphenylacetaldehyde	C1=CC(=C(C=C1CC=O)O)O	151.30>123.15	7.30	0.005-10	0.9995	2.0×10^3	0.005	0.005
3,4-Dihydroxyphenylacetic acid	C1=CC(=C(C=C1CC(=O)O)O)O	167.10>123.15	6.77	0.1-10	0.9993	1.0×10^2	0.1	0.010
Hydroxytyrosol	C1=CC(=C(C=C1CCO)O)O	153.00>123.05	7.20	0.5-10	0.9992	2.0×10^1	0.5	0.050
Methyldopa *	C[C@](CC1=CC(=C(C=C1)O)O)(C(=O)O)N	212.00>165.95	7.50	0.001-10	0.9999	1.0×10^4	0.001	0.0001

Table S1 (continued)

Metabolites	SMILES	MRM transition	Retention time (min)	Quantitative linear range (μM)	R^2	Dynamic range	Limit of quantitation (μM)	Limit of detection (μM)
Tyramine	C1=CC(=CC=C1CCN)O	138.00>121.15	11.29	0.001-1	0.9977	1.0×10^3	0.001	0.001
Hypoxanthine †	C1=NC2=C(N1)C(=O)NC=N2	137.00＞55.05	4.22	0.005-10	0.9969	2.0×10^3	0.005	0.0001
4-Aminobenzoic acid	C1=CC(=CC=C1C(=O)O)N	138.00>77.15	8.30	0.05-10	0.9999	2.0×10^2	0.05	0.050
4-Aminophenylalanine	C1=CC(=CC=C1C[C@@H](C(=O)O)N)N	181.00>122.10	3.50	0.01-10	0.9993	1.0×10^3	0.01	0.010
4-Aminophenylpyruvic acid	C1=CC(=CC=C1CC(=O)C(=O)O)N	180.20>91.20	4.40	0.05-10	0.9997	2.0×10^2	0.05	0.050
4-Hydroxybenzoic acid	C1=CC(=CC=C1C(=O)O)O	137.10>93.10	8.55	0.05-10	1.0000	2.0×10^2	0.05	0.0001
Caffeic acid *	C1=CC(=C(C=C1/C=C/C(=O)O)O)O	178.80>135.10	8.60	0.001-10	0.9998	1.0×10^4	0.001	0.001
Catechol	C1=CC=C(C(=C1)O)O	109.40>91.05	8.50	5-10	1.0000	2.0×10^0	5.000	5.000
Ferulic acid	COC1=C(C=CC(=C1)/C=C/C(=O)O)O	192.80>134.10	9.70	0.005-10	1.0000	2.0×10^3	0.005	0.005
4-Coumaric acid	C1=CC(=CC=C1C=CC(=O)O)O	162.80>119.20	9.30	0.005-10	1.0000	2.0×10^3	0.005	0.005
Protocatechualdehyde	C1=CC(=C(C=C1C=O)O)O	139.00>93.15	8.62	0.01-10	0.9994	1.0×10^3	0.01	0.010
Protocatechuic acid *	C1=CC(=C(C=C1C(=O)O)O)O	153.00>109.10	8.10	0.001-10	0.9997	1.0×10^4	0.001	0.001

Table S1 (continued)

Metabolites	SMILES	MRM transition	Retention time (min)	Quantitative linear range (μM)	R^2	Dynamic range	Limit of quantitation (μM)	Limit of detection (μM)
Salicylic acid	C1=CC=C(C(=C1)C(=O)O)O	137.00>93.00	12.47	0.01-10	0.9994	1.0×10^3	0.01	0.0001
Sinapic acid	COC1=CC(=CC(=C1O)OC)C=CC(=O)O	224.90>207.10	9.60	0.005-10	1.0000	2.0×10^3	0.005	0.005
Vanillic acid	COC1=C(C=CC(=C1)C(=O)O)O	169.10>93.10	8.77	0.5-10	0.9578	2.0×10^1	0.5	0.005
Vanillin	COC1=C(C=CC(=C1)C=O)O	153.30>65.00	9.88	0.01-10	0.9993	1.0×10^3	0.01	0.005
Indole	C1=CC=C2C(=C1)C=CN2	118.10>91.15	14.80	0.01-10	0.9984	1.0×10^3	0.01	0.001
5-Aminovaleric acid	C(CCN)CC(=O)O	118.10>101.10	5.60	0.005-10	0.9996	2.0×10^3	0.005	0.001
2-Oxoisopentanoic acid	CCCC(=O)C(=O)O	115.00>71.10	8.00	0.001-0.5	0.9904	5.0×10^2	0.001	0.100
5-Aminolevulinic acid *	C(CC(=O)O)C(=O)CN	132.10>86.25	3.70	0.0001-5	0.9985	5.0×10^4	0.0001	0.0001
2-Hydroxyisocaproic acid	CC(C)CC(C(=O)O)O	131.00>85.05	8.70	0.05-10	0.9874	2.0×10^2	0.05	0.050
4-Acetylbutyric acid	CC(=O)CCCC(=O)O	130.90>85.10	8.00	0.01-10	0.9923	1.0×10^3	0.01	0.005
Acetylcarnitine *†	CC(=O)O[C@H](CC(=O)[O-])C[N+](C)(C)C	204.10＞85.05	9.50	0.0001-1	0.9942	1.0×10^4	0.0001	0.0001
4-Aminobutyric acid *†	C(CC(=O)O)CN	104.10＞87.05	2.96	0.001-10	0.9922	1.0×10^4	0.001	0.001

Table S1 (continued)

Metabolites	SMILES	MRM transition	Retention time (min)	Quantitative linear range (μM)	R^2	Dynamic range	Limit of quantitation (μM)	Limit of detection (μM)
2-Aminobutyric acid *†	CCC(C(=O)O)N	104.10＞58.05	2.36	0.001-10	0.9982	1.0×10^4	0.001	0.001
Pipecolic acid	C1CCNC(C1)C(=O)O	130.00>84.10	4.70	0.005-10	0.9913	2.0×10^3	0.005	0.005
2-Isopropylmalic acid	CC(C)C(CC(=O)O)(C(=O)O)O	175.10>115.05	8.00	0.1-10	0.9980	1.0×10^2	0.1	0.005
2,6-Diaminopimelic acid	C(CC(C(=O)O)N)CC(C(=O)O)N	191.00>128.00	1.80	0.001-5	0.9961	5.0×10^3	0.001	0.0001
Creatine †	CN(CC(=O)O)C(=N)N	132.10＞44.05	3.06	0.005-1	0.9971	2.0×10^2	0.005	0.0001
Orotic acid †	C1=C(NC(=O)NC1=O)C(=O)O	155.00＞111.10	2.63	0.01-10	0.9985	1.0×10^3	0.01	0.005
Adenine †	C1=NC2=NC=NC(=C2N1)N	136.00＞119.05	6.50	0.005-5	0.9932	1.0×10^3	0.005	0.005
Cytosine *†	C1=C(NC(=O)N=C1)N	112.00＞95.10	3.82	0.001-10	0.9935	1.0×10^4	0.001	0.001
Guanine †	C1=NC2=C(N1)C(=O)NC(=N2)N	150.00＞133.00	5.00	0.01-10	0.9987	1.0×10^3	0.01	0.01
Thymine †	CC1=CNC(=O)NC1=O	127.10＞54.05	5.68	0.005-10	0.9969	2.0×10^3	0.005	0.005
Uracil †	C1=CNC(=O)NC1=O	113.00＞70.00	2.74	0.01-10	0.9979	1.0×10^3	0.01	0.005
Xanthine †	C1=NC2=C(N1)C(=O)NC(=O)N2	151.00＞108.00	4.22	0.005-10	0.9987	2.0×10^3	0.005	0.005

Table S1 (continued)

Metabolites	SMILES	MRM transition	Retention time (min)	Quantitative linear range (μM)	R^2	Dynamic range	Limit of quantitation (μM)	Limit of detection (μM)
Adenosine †	C1=NC(=C2C(=N1)N(C=N2)[C@H]3[C@@H]([C@@H]([C@H](O3)CO)O)O)N	268.10＞136.05	8.00	0.005-0.5	0.9949	1.0×10^2	0.005	0.0001
Cytidine †	C1=CN(C(=O)N=C1N)[C@H]2[C@@H]([C@@H]([C@H](O2)CO)O)O	244.10＞112.05	6.13	0.005-1	0.9962	2.0×10^2	0.005	0.0001
Guanosine †	C1=NC2=C(N1[C@H]3[C@@H]([C@@H]([C@H](O3)CO)O)O)N=C(NC2=O)N	284.00＞152.00	7.59	0.001-1	0.9964	1.0×10^3	0.001	0.0001
Inosine *†	C1=NC2=C(C(=O)N1)N=CN2[C@H]3[C@@H]([C@@H]([C@H](O3)CO)O)O	269.10＞137.05	7.49	0.0001-10	0.9935	1.0×10^5	0.0001	0.0001
Thymidine *†	CC1=CN(C(=O)NC1=O)[C@H]2C[C@@H]([C@H](O2)CO)O	243.10＞127.10	7.71	0.001-10	0.9990	1.0×10^4	0.001	0.0001
Uridine †	C1=CN(C(=O)NC1=O)[C@H]2[C@@H]([C@@H]([C@H](O2)CO)O)O	245.00＞113.05	4.34	0.01-10	0.9998	1.0×10^3	0.01	0.005
Adenosine 5'-monophosphoric acid †	C1=NC(=C2C(=N1)N(C=N2)[C@H]3[C@@H]([C@@H]([C@H](O3)COP(=O)(O)O)O)O)N	348.00＞136.05	2.98	0.005-10	1.0000	2.0×10^3	0.005	0.0001
Cytidine 3',5'-cyclic monophosphoric acid †	C1[C@@H]2[C@H]([C@H]([C@@H](O2)N3C=CC(=NC3=O)N)O)OP(=O)(O1)O	306.00＞112.10	3.99	0.005-10	0.9987	2.0×10^3	0.005	0.0001
Cytidine-5'-monophosphoric acid †	C1=CN(C(=O)N=C1N)[C@H]2[C@@H]([C@@H]([C@H](O2)COP(=O)(O)O)O)O	324.00＞112.05	2.05	0.005-10	0.9990	2.0×10^3	0.005	0.005
Guanosine 3',5'-cyclic monophosphoric acid †	C1[C@@H]2[C@H]([C@H]([C@@H](O2)N3C=NC4=C3N=C(NC4=O)N)O)OP(=O)(O1)O	346.00＞152.05	7.15	0.005-10	0.9999	2.0×10^3	0.005	0.005
Guanosine 5'-monophosphoric acid *†	C1=NC2=C(N1[C@H]3[C@@H]([C@@H]([C@H](O3)COP(=O)(O)O)O)O)N=C(NC2=O)N	364.00＞152.05	2.55	0.001-10	0.9999	1.0×10^4	0.001	0.001
Thymidine 5'-monophosphoric acid †	CC1=CN(C(=O)NC1=O)[C@H]2C[C@@H]([C@H](O2)COP(=O)(O)O)O	322.90＞81.10	3.29	0.005-10	0.9999	2.0×10^3	0.005	0.005
2'-Deoxyinosine	C1[C@@H]([C@H](O[C@H]1N2C=NC3=C2N=CNC3=O)CO)O	252.70>137.10	7.50	0.005-5	0.9988	1.0×10^3	0.005	0.005

Table S1 (continued)

Metabolites	SMILES	MRM transition	Retention time (min)	Quantitative linear range (μM)	R^2	Dynamic range	Limit of quantitation (μM)	Limit of detection (μM)
5'-Methylthioadenosine *	CSC[C@@H]1[C@H]([C@H]([C@@H](O1)N2C=NC3=C(N=CN=C32)N)O)O	298.00>135.95	10.20	0.0001-10	0.9933	1.0×10^5	0.0001	0.0001
5'-Deoxyadenosine	C[C@@H]1[C@H]([C@H]([C@@H](O1)N2C=NC3=C(N=CN=C32)N)O)O	252.20>136.15	8.90	0.001-0.5	0.9992	5.0×10^2	0.001	0.001
Adenylsuccinic acid †	C1=NC(=C2C(=N1)N(C=N2)[C@H]3[C@@H]([C@@H]([C@H](O3)COP(=O)(O)O)O)O)NC(CC(=O)O)C(=O)O	464.10＞252.10	7.23	0.005-10	0.9997	2.0×10^3	0.005	0.005
4-Hydroxyproline *†	C1C(CNC1C(=O)O)O	132.10＞86.05	1.79	0.001-10	0.9980	1.0×10^4	0.001	0.001
Alanine †	C[C@@H](C(=O)O)N	89.90＞44.10	1.99	0.01-10	0.9982	1.0×10^3	0.01	0.01
Arginine †	C(C[C@@H](C(=O)O)N)CN=C(N)N	175.10＞70.10	2.75	0.005-10	0.9948	2.0×10^3	0.005	0.005
Asparagine †	C([C@@H](C(=O)O)N)C(=O)N	133.10＞87.15	1.76	0.005-10	0.9994	2.0×10^3	0.005	0.001
Aspartic acid †	C([C@@H](C(=O)O)N)C(=O)O	134.00＞74.05	1.76	0.01-10	0.9992	1.0×10^3	0.01	0.005
Asymmetric dimethylarginine *†	CN(C)C(=NCCC[C@@H](C(=O)O)N)N	203.10＞70.10	5.47	0.001-10	0.9950	1.0×10^4	0.001	0.0001
Citrulline *†	C(C[C@@H](C(=O)O)N)CNC(=O)N	176.10＞70.05	2.07	0.001-10	0.9956	1.0×10^4	0.001	0.0001
Cystine *†	C([C@@H](C(=O)O)N)SSC[C@@H](C(=O)O)N	241.00＞151.95	1.77	0.0001-10	0.9990	1.0×10^5	0.0001	0.0001
Dimethylglycine †	CN(C)CC(=O)O	104.10＞58.05	1.96	0.005-10	0.9930	2.0×10^3	0.005	0.0001

Table S1 (continued)

Metabolites	SMILES	MRM transition	Retention time (min)	Quantitative linear range (μM)	R^2	Dynamic range	Limit of quantitation (μM)	Limit of detection (μM)
Glutamic acid [†]	C(CC(=O)O)[C@@H](C(=O)O)N	147.90 > 84.10	1.99	0.01-10	0.9995	1.0×10^3	0.01	0.0001
Glutamine [†]	C(CC(=O)N)[C@@H](C(=O)O)N	147.10 > 84.15	1.85	0.01-10	0.9974	1.0×10^3	0.01	0.005
Glycine [†]	C(C(=O)O)N	75.90 > 30.15	1.79	0.01-10	0.9978	1.0×10^3	0.01	0.01
Homocystine [*†]	C(CSSCCC(C(=O)O)N)C(C(=O)O)N	269.00 > 136.05	4.02	0.001-10	0.9997	1.0×10^4	0.001	0.001
Isoleucine [†]	CC[C@H](C)[C@@H](C(=O)O)N	132.10 > 86.20	8.01	0.005-10	0.9962	2.0×10^3	0.005	0.0001
Leucine [†]	CC(C)C[C@@H](C(=O)O)N	132.10 > 86.05	8.27	0.01-10	0.9952	1.0×10^3	0.01	0.0001
Lysine [†]	C(CCN)C[C@@H](C(=O)O)N	147.10 > 84.10	2.42	0.01-10	0.9995	1.0×10^3	0.01	0.0001
Methionine [*†]	CSCC[C@@H](C(=O)O)N	149.90 > 56.10	4.49	0.001-10	0.9970	1.0×10^4	0.001	0.001
Methionine sulfoxide [*†]	CS(=O)CC[C@@H](C(=O)O)N	166.00 > 74.10	1.98	0.001-10	0.9991	1.0×10^4	0.001	0.005
Ornithine [†]	C(C[C@@H](C(=O)O)N)CN	133.10 > 70.10	2.22	0.05-10	0.9964	2.0×10^2	0.05	0.01
Phenylalanine [†]	C1=CC=C(C=C1)C[C@@H](C(=O)O)N	166.10 > 120.10	8.95	0.005-5	0.9988	1.0×10^3	0.005	0.0001

Table S1 (continued)

Metabolites	SMILES	MRM transition	Retention time (min)	Quantitative linear range (μM)	R^2	Dynamic range	Limit of quantitation (μM)	Limit of detection (μM)
Proline *†	C1C[C@H](NC1)C(=O)O	116.10＞70.15	2.34	0.001-10	0.9966	1.0×10^4	0.001	0.001
Serine †	C([C@@H](C(=O)O)N)O	105.60＞60.10	1.74	0.05-10	0.9992	2.0×10^2	0.05	0.001
Symmetric dimethylarginine *†	CNC(=NC)NCCC[C@@H](C(=O)O)N	203.10＞70.15	5.37	0.001-10	0.9675	1.0×10^4	0.001	0.001
Threonine *†	C[C@H]([C@@H](C(=O)O)N)O	120.10＞74.15	1.89	0.001-10	0.9976	1.0×10^4	0.001	0.001
Tryptophan †	C1=CC=C2C(=C1)C(=CN2)C[C@@H](C(=O)O)N	205.10＞188.15	10.76	0.005-5	0.9997	1.0×10^3	0.005	0.001
Tyrosine *†	C1=CC(=CC=C1C[C@@H](C(=O)O)N)O	182.10＞136.10	7.82	0.001-10	0.9985	1.0×10^4	0.001	0.0001
Valine *†	CC(C)[C@@H](C(=O)O)N	118.10＞72.15	4.59	0.001-10	0.9939	1.0×10^4	0.001	0.0001
AICAR †	C1=NC(=C(N1[C@H]2[C@@H]([C@@H]([C@H](O2)COP(=O)(O)O)O)O)N)C(=O)N	337.20＞79.05	2.42	0.005-10	0.9986	2.0×10^3	0.005	0.005
Histidinol †	C1=C(NC=N1)CC(CO)N	142＞81.15	3.38	0.001-1	0.9981	1.0×10^3	0.001	0.0001
Ergothioneine *†	C[N+](C)(C)[C@@H](CC1=CNC(=S)N1)C(=O)[O-]	230.10＞127.10	2.53	0.001-10	0.9997	1.0×10^4	0.001	0.001
Argininosuccinic acid †	C(C[C@@H](C(=O)O)N)CN=C(N)N[C@@H](CC(=O)O)C(=O)O	291.00＞70.10	2.60	0.005-10	0.9998	2.0×10^3	0.005	0.005
Ophthalmic acid *†	CC[C@@H](C(=O)NCC(=O)O)NC(=O)CC[C@@H](C(=O)O)N	290.10＞58.10	4.95	0.001-10	0.9993	1.0×10^4	0.001	0.001

Table S1 (continued)

Metabolites	SMILES	MRM transition	Retention time (min)	Quantitative linear range (µM)	R^2	Dynamic range	Limit of quantitation (µM)	Limit of detection (µM)
Urocanic acid †	C1=C(NC=N1)/C=C/C(=O)O	137.50＞93.10	7.74	0.01-10	0.9990	1.0×10^3	0.01	0.005
Carnitine †	C[N+](C)(C)CC(CC(=O)[O-])O	162.10＞103.05	4.70	0.005-1	0.9965	2.0×10^2	0.005	0.0001
Trimethylamine *	CN(C)C	60.03>44.10	4.50	0.001-10	0.9806	1.0×10^4	0.001	0.001
Cadaverine *	C(CCN)CCN	103.20>86.15	4.50	0.0001-1	0.9942	1.0×10^4	0.0001	0.0001
Norspermidine	C(CN)CNCCCN	132.20>98.25	6.50	0.5-10	0.9971	2.0×10^1	0.5	0.005
Trimethylamine N-oxide	C[N+](C)(C)[O-]	76.00>58.00	5.00	0.001-5	0.9897	5.0×10^3	0.001	0.001
Putrescine	C(CCN)CN	89.20>72.20	3.70	0.01-1	0.9912	1.0×10^2	0.01	0.001
N1,N8-Diacetylspermidine	CC(=O)NCCCCNCCCNC(=O)C	230.10>100.00	9.70	0.0001-0.5	0.9984	5.0×10^3	0.0001	0.0001
Cysteamine †	C(CS)N	78.10＞61.05	3.32	0.05-10	0.9905	2.0×10^2	0.05	0.001
Dopamine *†	C1=CC(=C(C=C1CCN)O)O	154.10＞91.05	8.62	0.0001-10	0.9993	1.0×10^5	0.0001	0.0001
Histamine †	C1=C(NC=N1)CCN	112.10＞95.05	3.52	0.001-1	0.9992	1.0×10^3	0.001	0.001
Serotonin *†	C1=CC2=C(C=C1O)C(=CN2)CCN	177.10＞77.05	10.99	0.001-10	0.9939	1.0×10^4	0.001	0.001

Table S1 (continued)

Metabolites	SMILES	MRM transition	Retention time (min)	Quantitative linear range (μM)	R^2	Dynamic range	Limit of quantitation (μM)	Limit of detection (μM)
N-Acetyl-L-valine	CC(C)[C@@H](C(=O)O)NC(=O)C	160.00>72.10	8.00	0.005-10	0.9952	2.0×10^3	0.005	0.005
N2-Phenylacetylglutamine *	C1=CC=C(C=C1)CC(=O)NC(CCC(=O)N)C(=O)O	264.90>130.05	8.30	0.001-10	0.9928	1.0×10^4	0.001	0.0001
γ-Butyrobetaine	C[N+](C)(C)CCCC(=O)[O-]	146.10>87.10	7.70	0.005-1	0.9944	2.0×10^2	0.005	0.005
Taurine	C(CS(=O)(=O)O)N	124.10>80.00	1.50	0.05-5	0.9993	1.0×10^2	0.05	0.001
N-ε-Acetyl-L-lysine *	CC(=O)NCCCC[C@@H](C(=O)O)N	189.00>84.00	5.10	0.001-10	0.9990	1.0×10^4	0.001	0.001
N-γ-Ethyl-L-glutamine *	CCNC(=O)CC[C@@H](C(=O)O)N	175.00>83.95	4.20	0.001-10	0.9986	1.0×10^4	0.001	0.001
L-Cysteine S-sulfate	C([C@@H](C(=O)O)N)SS(=O)(=O)O	202.00>120.00	1.60	0.05-10	0.9999	2.0×10^2	0.05	0.050
Cycloleucine	C1CCC(C1)(C(=O)O)N	130.00>84.05	7.30	0.005-5	1.0000	1.0×10^3	0.005	0.005
3-Indoxylsulfuric acid	C1=CC=C2C(=C1)C(=CN2)OS(=O)(=O)O	212.00>80.05	7.70	0.5-10	0.9804	2.0×10^1	0.5	0.005
Indole-3-acetic acid	C1=CC=C2C(=C1)C(=CN2)CC(=O)O	175.80>130.00	10.90	0.005-10	0.9992	2.0×10^3	0.005	0.001
Methyl sulfate *	COS(=O)(=O)O	111.10>79.90	1.45	0.001-10	0.9944	1.0×10^4	0.001	0.0001
Vanillylmandelic acid *	COC1=C(C=CC(=C1)C(C(=O)O)O)O	197.00>137.20	7.70	0.001-10	0.9992	1.0×10^4	0.001	0.001

Table S1 (continued)

Metabolites	SMILES	MRM transition	Retention time (min)	Quantitative linear range (μM)	R^2	Dynamic range	Limit of quantitation (μM)	Limit of detection (μM)
1-Methyl-2-pyrrolidinone	CN1CCCC1=O	100.00>58.05	7.70	0.005-10	0.9957	1.0×10^3	0.005	0.001
6-Methylaminopurine *	CNC1=NC=NC2=C1NC=N2	150.30>108.00	8.70	0.0001-1	0.9990	1.0×10^4	0.0001	0.0001
Proline betaine	C[N+]1(CCC[C@H]1C(=O)[O-])C	144.10>58.25	3.40	0.0001-0.5	0.9959	5.0×10^3	0.0001	0.0001
Uric acid †	C12=C(NC(=O)N1)NC(=O)NC2=O	167.10＞123.95	3.25	0.005-10	0.9983	2.0×10^3	0.005	0.001
Acetylcholine †	CC(=O)OCC[N+](C)(C)C	147.10＞87.05	10.00	0.005-1	0.9992	2.0×10^2	0.005	0.0001
Allantoin †	C1(C(=O)NC(=O)N1)NC(=O)N	157.00＞97.10	1.78	0.01-10	0.9981	1.0×10^3	0.01	0.001
Choline †	C[N+](C)(C)CCO	104.10＞60.05	3.90	0.005-5	0.9918	1.0×10^3	0.005	0.005
Citicoline †	C[N+](C)(C)CCOP(=O)([O-])OP(=O)(O)OC[C@@H]1[C@H]([C@H]([C@@H](O1)N2C=CC(=NC2=O)N)O)O	489.10＞184.10	2.00	0.005-10	0.9986	2.0×10^3	0.005	0.0001
Creatinine †	CN1CC(=O)N=C1N	114.10＞44.05	4.20	0.005-1	0.9921	2.0×10^2	0.005	0.005
Dopa †	C1=CC(=C(C=C1C[C@@H](C(=O)O)N)O)O	198.10＞152.10	6.56	0.005-10	0.9993	2.0×10^3	0.005	0.0001
Adrenaline *†	CNC[C@@H](C1=CC(=C(C=C1)O)O)O	184.10＞166.10	7.63	0.001-10	0.9909	1.0×10^4	0.001	0.0001
Kynurenine *†	C1=CC=C(C(=C1)C(=O)C[C@@H](C(=O)O)N)N	209.10＞192.05	9.20	0.001-10	0.9970	1.0×10^4	0.001	0.001
Noradrenaline †	C1=CC(=C(C=C1[C@H](CN)O)O)O	170.10＞152.15	4.09	0.01-5	0.9984	5.0×10^2	0.01	0.010

Table S1 (continued)

Metabolites	SMILES	MRM transition	Retention time (min)	Quantitative linear range (μM)	R^2	Dynamic range	Limit of quantitation (μM)	Limit of detection (μM)
Thiamine	CC1=C(SC=[N+]1CC2=CN=C(N=C2N)C)CCO	265.50>122.20	0.67	0.005-1	0.9925	2.0×10^2	0.005	0.0001
Saccharic acid	[C@H]([C@@H]([C@@H](C(=O)O)O)O)([C@H](C(=O)O)O)O	209.10>85.00	0.96	0.005-10	0.9999	2.0×10^3	0.005	0.005
4-Oxopentanoic acid	CC(=O)CCC(=O)O	117.00>99.25	1.58	0.05-10	0.9987	2.0×10^2	0.05	0.005
Spermidine	C(CCNCCCN)CN	146.20>72.15	0.60	0.001-0.5	0.9999	5.0×10^2	0.001	0.001
Spermine	C(CCNCCCN)CNCCCN	203.10>112.30	0.60	0.01-10	0.9930	1.0×10^3	0.01	0.010
2-Phenylethylamine *	C1=CC=C(C=C1)CCN	122.20>105.25	1.60	0.0001-1	0.9981	1.0×10^4	0.0001	0.0001
N1-Acetylspermine *	CC(=O)NCCCNCCCCNCCCN	245.10>112.15	0.63	0.001-10	0.9924	1.0×10^4	0.001	0.001
N1-Acetylspermidine	CC(=O)NCCCNCCCCN	188.20>72.25	0.70	0.0001-0.05	0.9984	5.0×10^2	0.0001	0.0001
Tryptamine *	C1=CC=C2C(=C1)C(=CN2)CCN	161.20>144.35	2.00	0.0001-1	0.9962	1.0×10^4	0.0001	0.0001
α-Methylbenzylamine	CC(C1=CC=CC=C1)N	122.30>105.25	1.32	0.05-10	0.9805	2.0×10^2	0.05	0.050
N-Carbamoyl-L-aspartic acid *	C([C@@H](C(=O)O)NC(=O)N)C(=O)O	176.90>88.10	1.03	0.001-10	0.9995	1.0×10^4	0.001	0.0001
Serine O-sulfate *	C([C@@H](C(=O)O)N)OS(=O)(=O)O	184.20>96.95	1.11	0.001-10	0.9958	1.0×10^4	0.001	0.0001

Table S1 (continued)

Metabolites	SMILES	MRM transition	Retention time (min)	Quantitative linear range (μM)	R^2	Dynamic range	Limit of quantitation (μM)	Limit of detection (μM)
N-α-Benzoylarginine ethylester	CCOC(=O)[C@H](CCCN=C(N)N)NC(=O)C1=CC=CC=C1	307.00>104.90	3.46	0.001-0.5	0.9946	5.0×10^2	0.001	0.0001
Cysteine-glutathione disulphide *	C(CC(=O)N[C@@H](CSSC[C@@H](C(=O)O)N)C(=O)NCC(=O)O)[C@@H](C(=O)O)N	427.30>298.15	0.85	0.0001-10	0.9990	1.0×10^5	0.0001	0.0001
Phe-Phe	C1=CC=C(C=C1)C[C@@H](C(=O)N[C@@H](CC2=CC=CC=C2)C(=O)O)N	313.20>120.10	3.88	0.0001-0.5	0.9961	5.0×10^3	0.0001	0.0001
Sorbitol 6-phosphate	C([C@@H]([C@H]([C@@H]([C@@H](COP(=O)(O)O)O)O)O)O)O	263.00>180.90	0.67	0.01-10	0.9975	1.0×10^3	0.01	0.0001
Picolinic acid butyl ester	CCCCOC(=O)C1=CC=CC=N1	180.30>78.15	11.42	0.0001-0.5	0.9893	5.0×10^3	0.0001	0.0001
4-Hydroxymethylimidazole *	C1=C(NC=N1)CO	99.30>81.15	0.77	0.001-10	0.9930	1.0×10^4	0.001	0.0001
Pyruvic acid	CC(=O)C(=O)O	357.20>137.00	12.76	0.01-10	0.9914	1.0×10^3	0.01	0.01
2-Oxoglutaric acid *	C(CC(=O)O)C(=O)C(=O)O	552.20>399.30	13.60	0.0001-1	0.9997	1.0×10^4	0.0001	0.0001
Deoxycholic acid *	C[C@H](CCC(=O)O)[C@H]1CC[C@@H]2[C@@]1([C@H](C[C@H]3[C@H]2CC[C@H]4[C@@]3(CC[C@H](C4)O)C)O)C	526.20>152.20	15.70	0.0001-1	0.9996	1.0×10^4	0.0001	0.0001
Hyodeoxycholic acid *	C[C@H](CCC(=O)O)[C@H]1CC[C@@H]2[C@@]1(CC[C@H]3[C@H]2C[C@@H]([C@H]4[C@@]3(CC[C@H](C4)O)C)O)C	526.20>137.15	14.20	0.0001-1	0.9989	1.0×10^4	0.0001	0.0001
Lithocholic acid	C[C@H](CCC(=O)O)[C@H]1CC[C@@H]2[C@@]1(CC[C@H]3[C@H]2CC[C@H]4[C@@]3(CC[C@H](C4)O)C)C	510.20>137.15	17.30	0.001-0.5	0.9997	5.0×10^2	0.001	0.0001
Ursodeoxycholic acid	C[C@H](CCC(=O)O)[C@H]1CC[C@@H]2[C@@]1(CC[C@H]3[C@H]2[C@H](C[C@H]4[C@@]3(CC[C@H](C4)O)C)O)C	526.20>137.15	14.30	0.001-0.5	0.9998	5.0×10^2	0.001	0.001

Table S1 (continued)

Metabolites	SMILES	MRM transition	Retention time (min)	Quantitative linear range (μM)	R^2	Dynamic range	Limit of quantitation (μM)	Limit of detection (μM)
Chenodeoxycholic acid	C[C@H](CCC(=O)O)[C@H]1CC[C@@H]2[C@@]1(CC[C@H]3[C@H]2[C@@H](C[C@H]4[C@@]3(CC[C@H](C4)O)C)O)C	526.30>137.15	15.40	0.001-1	0.9985	1.0×10^3	0.001	0.0001
3-(4-Hydroxyphenyl)propionic acid *	C1=CC(=CC=C1CCC(=O)O)O	302.20>107.10	9.80	0.0001-10	0.9991	1.0×10^5	0.0001	0.0001
4-Hydroxyphenylacetic acid	C1=CC(=CC=C1CC(=O)O)O	287.80>107.10	8.85	0.001-5	0.9966	5.0×10^3	0.001	0.0001
2,3-Pyridinedicarboxylic acid	C1=CC(=C(N=C1)C(=O)O)C(=O)O	438.00>285.05	11.79	0.001-0.5	0.9989	5.0×10^2	0.001	0.001
Benzoic acid	C1=CC=C(C=C1)C(=O)O	258.00>105.15	11.60	0.01-5	0.9998	5.0×10^2	0.01	0.001
3-Hydroxybenzoic acid *	C1=CC(=CC(=C1)O)C(=O)O	273.80>121.05	9.70	0.001-10	0.9991	1.0×10^4	0.001	0.001
3-Phenylpropionic acid *	C1=CC=C(C=C1)CCC(=O)O	285.90>105.15	12.70	0.0001-5	0.9956	5.0×10^4	0.0001	0.0001
Malonic acid	C(C(=O)O)C(=O)O	373.10>178.10	10.70	0.005-10	0.9973	2.0×10^3	0.005	0.0001
Crotonic acid *	C/C=C/C(=O)O	221.90>69.10	9.20	0.0001-10	0.9989	1.0×10^5	0.0001	0.0001
Dodecanoic acid	CCCCCCCCCCCC(=O)O	334.10>144.95	17.00	0.01-5	0.9857	5.0×10^2	0.01	0.01
2-Hydroxypentanoic acid *	CCCC(C(=O)O)O	252.20>152.15	9.10	0.0001-10	0.9957	1.0×10^5	0.0001	0.0001
2-Oxoisocaproic acid	CC(C)CC(=O)C(=O)O	399.30>150.00	15.10	0.05-10	0.9985	2.0×10^2	0.05	0.001

Table S1 (continued)

Metabolites	SMILES	MRM transition	Retention time (min)	Quantitative linear range (μM)	R^2	Dynamic range	Limit of quantitation (μM)	Limit of detection (μM)
Butyric acid	CCCC(=O)O	224.10>71.05	9.60	0.01-10	0.9987	1.0×10^3	0.01	0.0001
Propionic acid	CCC(=O)O	210.10>57.15	7.00	0.01-10	0.9971	1.0×10^3	0.01	0.0001
Isobutyric acid *	CC(C)C(=O)O	224.10>71.05	9.30	0.001-10	0.9980	1.0×10^4	0.001	0.001
Valeric acid *	CCCCC(=O)O	238.10>85.05	11.50	0.001-10	0.9982	1.0×10^4	0.001	0.0001
Isovaleric acid	CC(C)CC(=O)O	238.10>85.05	11.19	0.01-10	0.9899	1.0×10^3	0.01	0.0001
2-Oxobutyric acid *	CCC(=O)C(=O)O	373.00>220.00	14.20	0.001-10	0.9990	1.0×10^4	0.001	0.0001
3-Hydroxybutyric acid *	CC(CC(=O)O)O	240.10>154.00	4.50	0.001-10	0.9977	1.0×10^4	0.001	0.0001
2-Hydroxyglutaric acid	C(CC(=O)O)C(C(=O)O)O	419.10>266.00	10.50	0.001-5	0.9987	5.0×10^3	0.001	0.0001
Prostaglandin E2	CCCCC[C@@H](/C=C/[C@H]1[C@@H](CC(=O)[C@@H]1C/C=C\\CCCC(=O)O)O)O	488.30>452.25	14.90	0.005-5	0.9941	1.0×10^3	0.005	0.010

Table S2. Displayed retention-time windows for the extracted ion chromatograms in Figure 3. Start and end times (min) of the displayed retention-time window for each chromatograms shown in Figure 3

(A) Nonderivatized: PFPP column

Metabolites	Time (min)
Biotin	7.20-9.20
Pyridoxine	8.00-10.00
Riboflavin	7.00-9.00
5-Aminolevulinic acid	3.04-4.52
2-Oxoisopentanoic acid	7.00-9.04
5-Aminovaleric acid	4.70-6.70
2-Hydroxyisocaproic acid	8.04-10.02
4-Acetylbutyric acid	7.58-8.41
Pipecolic acid	3.44-5.00
2-Isopropylmalic acid	7.00-9.00
2,6-Diaminopimelic acid	0.76-2.76
2'-Deoxyinosine	6.50-8.60
5'-Methylthioadenosine	9.02-10.96
5'-Deoxyadenosine	8.00-10.00
Trimethylamine	3.50-6.00
Cadaverine	3.45-5.55
Norspermidine	5.70-7.60
Trimethylamine N-oxide	4.02-6.00
Putrescine	2.72-4.70
N1,N8-Diacetylspermidine	8.50-11.10
N-Acetyl-L-valine	7.02-8.96
N2-Phenylacetylglutamine	7.32-9.30
γ-Butyrobetaine	6.70-8.70
Taurine	0.00-2.00
N-ε-Acetyl-L-lysine	4.04-6.02
N-γ-Ethyl-L-glutamine	3.05-5.40
L-Cysteine S-sulfate	0.52-2.54
Cycloleucine	6.50-8.54
3-Indoxylsulfuric acid	6.70-8.70
Indole-3-acetic acid	10.00-11.98
Methyl sulfate	0.00-2.55
Vanillylmandelic acid	7.04-8.48
1-Methyl-2-pyrrolidinone	7.04-9.00
6-Methylaminopurine	8.04-9.54
Proline betaine	2.52-4.46

(B) Nonderivatized: C18 column

Metabolites	Time (min)
Thiamine	0.00-1.48
Saccharic acid	0.00-2.04
4-Oxopentanoic acid	0.60-2.50
Spermidine	0.00-1.44
Spermine	0.00-1.52
2-Phenylethylamine	0.00-2.50
N1-Acetylspermine	0.00-1.04
N1-Acetylspermidine	0.00-1.54
Tryptamine	1.58-3.02
α-Methylbenzylamine	0.48-2.48
N1,N12-Diacetylspermine	0.00-1.52
N-Carbamoyl-L-aspartic acid	0.00-2.04
Serine O-sulfate	0.52-2.00
N-α-Benzoylarginine ethylester	2.56-4.46
Cysteine-glutathione disulphide	0.00-1.98
Phe-Phe	3.04-4.98
Sorbitol 6-phosphate	0.00-1.06
Picolinic acid butyl ester	10.52-12.48
4-Hydroxymethylimidazole	0.00-2.05

(C) Derivatized: C18 column

Metabolites	Time (min)
Pyruvic acid	11.68-13.68
2-Oxoglutaric acid	12.50-14.50
Deoxycholic acid	14.98-16.48
Hyodeoxycholic acid	13.50-15.06
Lithocholic acid	16.52-18.50
Ursodeoxycholic acid	13.54-15.50
Chenodeoxycholic acid	14.54-16.44
3-(4-Hydroxyphenyl)propionic acid	9.02-11.00
4-Hydroxyphenylacetic acid	8.04-9.52
2,3-Pyridinedicarboxylic acid	11.00-12.54
Benzoic acid	10.52-12.54
3-Hydroxybenzoic acid	9.00-10.98
3-Phenylpropionic acid	11.50-13.54
Malonic acid	9.60-11.62
Maleic acid	10.70-12.70
Crotonic acid	8.54-10.04
Dodecanoic acid	16.48-17.90
2-Hydroxypentanoic acid	8.50-9.96
2-Oxoisocaproic acid	14.24-16.02
Butyric acid	8.60-10.60
Propionic acid	6.00-8.00
Isobutyric acid	8.40-10.38
Valeric acid	10.60-12.60
Isovaleric acid	10.16-12.16
2-Oxobutyric acid	13.28-15.26
2-Hydroxyglutaric acid	9.64-11.56
3-Hydroxybutyric acid	2.40-7.60
Prostaglandin E2	14.00-15.94

Table S3. Comparison of KUSLAMS with our previous PFPP-based method and a representative 2D-LC×LC–MS workflow, including analyte coverage, total instrument time, and validation items. * For the offline 2D-LC×LC–MS workflow, one original sample is first separated by the 1D LC (28 min) and fractionated into 50 fractions; each fraction is then reinjected and analyzed by the 2D LC (17 min per fraction), resulting in a total LC time of 28 + (17 × 50) = 878 min per original sample (excluding additional overhead such as equilibration and fraction handling).

Mthod	Number of target metabolietes	Target metabolites	Columns / concept	Single mobile phase across columns?	Injection per sample	Run time per injection (min)	Total time per sample (min)	Switching/valving	Validation completeness	Reference
This study	215	Primary and secondary metabolites	PFPP + C18 (+ deriv-C18) serial	Yes	3	30/ 23/ 23	76	batch-level switching	✓	
Our privious method	113	Primary metabolites	PFPP only	No	1	30	30	none	✓	13
2D-LC–MS	355	Primary and secondary metabolites	1D×2D (C18+HILIC)	No	1 (1D) + 50 (2D)	28 (1D) / 17 (2D)	28 + (17 × 50) = 878*	none	-	14

Table S4. Instrumental validation of peak area repeatability using intracellular metabolite extracts from gut microbiota. Repeatability was assessed using six replicate injections (n = 6) of a single sample. CVs were calculated from the MRM peak areas for each metabolite.

Metabolites	CV (%)	Metabolites	CV (%)
Biotin	16.00	N1-Acetylspermine	4.45
Pyridoxine	21.74	N1-Acetylspermidine	2.58
Riboflavin	5.76	Tryptamine	1.57
5-Aminovaleric acid	1.07	α-Methylbenzylamine	Not detected
2-Oxoisopentanoic acid	Not detected	N1,N12-Diacetylspermine	2.44
5-Aminolevulinic acid	Not detected	N-Carbamoyl-L-aspartic acid	4.69
2-Hydroxyisocaproic acid	13.88	Serine O-sulfate	Not detected
4-Acetylbutyric acid	26.22	N-α-Benzoylarginine ethylester	30.65
Pipecolic acid	5.11	Cysteine-glutathione disulphide	Not detected
2-Isopropylmalic acid	13.13	Phe-Phe	9.71
2,6-Diaminopimelic acid	2.79	Sorbitol 6-phosphate	Not detected
2'-Deoxyinosine	7.36	Picolinic acid butyl ester	Not detected
5'-Methylthioadenosine	1.88	4-Hydroxymethylimidazole	9.85
5'-Deoxyadenosine	2.96	Pyruvic acid	1.41
Trimethylamine	4.92	2-Oxoglutaric acid	7.92
Cadaverine	2.29	Deoxycholic acid	Not detected
Norspermidine	Not detected	Hyodeoxycholic acid	Not detected
Trimethylamine N-oxide	Not detected	Lithocholic acid	2.14
Putrescine	5.49	Ursodeoxycholic acid	Not detected
N1,N8-Diacetylspermidine	5.27	Chenodeoxycholic acid	Not detected
N-Acetyl-L-valine	7.12	3-(4-Hydroxyphenyl)propionic acid	2.23
N2-Phenylacetylglutamine	Not detected	4-Hydroxyphenylacetic acid	3.56
γ-Butyrobetaine	7.38	2,3-Pyridinedicarboxylic acid	Not detected
Taurine	18.65	Benzoic acid	2.54
N-ε-Acetyl-L-lysine	4.50	3-Hydroxybenzoic acid	Not detected
N-γ-Ethyl-L-glutamine	11.26	3-Phenylpropionic acid	0.95
L-Cysteine S-sulfate	19.07	Malonic acid	3.23
Cycloleucine	9.46	Maleic acid	20.39
3-Indoxylsulfuric acid	Not detected	Crotonic acid	10.02
Indole-3-acetic acid	3.81	Dodecanoic acid	Not detected
Methyl sulfate	15.12	2-Hydroxypentanoic acid	0.68
Vanillylmandelic acid	Not detected	2-Oxoisocaproic acid	9.52
1-Methyl-2-pyrrolidinone	20.02	Butyric acid	6.65
6-Methylaminopurine	26.00	Propionic acid	3.20
Proline betaine	2.02	Isobutyric acid	18.65
Thiamine	7.38	Valeric acid	5.65
Saccharic acid	22.76	Isovaleric acid	8.79
4-Oxopentanoic acid	11.34	2-Oxobutyric acid	6.65
Spermidine	1.60	2-Hydroxyglutaric acid	2.76
Spermine	85.20	3-Hydroxybutyric acid	3.51
2-Phenylethylamine	6.44	Prostaglandin E2	Not detected

Table S5. Evaluation of carryover after injection of a high-concentration standard mix. Carryover (%) was calculated for each metabolite from the ratio of MRM peak areas in a blank injection performed immediately after the 10 μM standard mix.

Metabolites	Carryover (%)	Metabolites	Carryover (%)
Biotin	0.018	N1-Acetylspermine	0.001
Pyridoxine	0.009	N1-Acetylspermidine	0.018
Riboflavin	0.011	Tryptamine	0.003
5-Aminovaleric acid	Not detected in blank	α-Methylbenzylamine	Not detected in blank
2-Oxoisopentanoic acid	0.832	N1,N12-Diacetylspermine	0.009
5-Aminolevulinic acid	Not detected in blank	N-Carbamoyl-L-aspartic acid	0.008
2-Hydroxyisocaproic acid	0.024	Serine O-sulfate	0.001
4-Acetylbutyric acid	1.481	N-α-Benzoylarginine ethylester	0.044
Pipecolic acid	0.008	Cysteine-glutathione disulphide	Not detected in blank
2-Isopropylmalic acid	0.063	Phe-Phe	0.020
2,6-Diaminopimelic acid	0.001	Sorbitol 6-phosphate	1.269
2'-Deoxyinosine	Not detected in blank	Picolinic acid butyl ester	0.010
5'-Methylthioadenosine	0.033	4-Hydroxymethylimidazole	0.010
5'-Deoxyadenosine	0.014	Pyruvic acid	0.019
Trimethylamine	Not detected in blank	2-Oxoglutaric acid	0.020
Cadaverine	0.039	Deoxycholic acid	0.020
Norspermidine	0.007	Hyodeoxycholic acid	0.009
Trimethylamine N-oxide	Not detected in blank	Lithocholic acid	0.223
Putrescine	Not detected in blank	Ursodeoxycholic acid	0.007
N1,N8-Diacetylspermidine	0.006	Chenodeoxycholic acid	0.020
N-Acetyl-L-valine	0.009	3-(4-Hydroxyphenyl)propionic acid	0.011
N2-Phenylacetylglutamine	0.025	4-Hydroxyphenylacetic acid	Not detected in blank
γ-Butyrobetaine	0.003	2,3-Pyridinedicarboxylic acid	0.008
Taurine	0.040	Benzoic acid	0.002
N-ε-Acetyl-L-lysine	0.009	3-Hydroxybenzoic acid	Not detected in blank
N-γ-Ethyl-L-glutamine	0.002	3-Phenylpropionic acid	0.005
L-Cysteine S-sulfate	0.041	Malonic acid	Not detected in blank
Cycloleucine	Not detected in blank	Maleic acid	0.050
3-Indoxylsulfuric acid	Not detected in blank	Crotonic acid	0.002
Indole-3-acetic acid	0.011	Dodecanoic acid	0.871
Methyl sulfate	0.001	2-Hydroxypentanoic acid	Not detected in blank
Vanillylmandelic acid	0.023	2-Oxoisocaproic acid	0.339
1-Methyl-2-pyrrolidinone	0.173	Butyric acid	0.050
6-Methylaminopurine	0.019	Propionic acid	Not detected in blank
Proline betaine	Not detected in blank	Isobutyric acid	Not detected in blank
Thiamine	Not detected in blank	Valeric acid	Not detected in blank
Saccharic acid	Not detected in blank	Isovaleric acid	0.001
4-Oxopentanoic acid	8.308	2-Oxobutyric acid	0.050
Spermidine	0.030	2-Hydroxyglutaric acid	Not detected in blank
Spermine	0.040	3-Hydroxybutyric acid	Not detected in blank
2-Phenylethylamine	0.103	Prostaglandin E2	Not detected in blank

Table S6. Inter-day validation of repeatability using intracellular metabolite extracts from gut microbiota. Inter-day precision was assessed by analyzing the same intracellular metabolite extract on three separate days (n = 2 per day; total n = 6). CVs were calculated from MRM peak areas and calculated concentrations.

Metabolites	Peak-area CV (%)	Concentration CV (%)	Metabolites	Peak-area CV (%)	Concentration CV (%)
Biotin	23.81	22.01	N1-Acetylspermine	7.47	5.52
Pyridoxine	8.17	6.76	N1-Acetylspermidine	5.41	4.22
Riboflavin	10.80	9.33	Tryptamine	5.35	2.45
5-Aminovaleric acid	Saturated	Saturated	α-Methylbenzylamine	Not detected	Not detected
2-Oxoisopentanoic acid	Not detected	Not detected	N1,N12-Diacetylspermine	8.30	9.88
5-Aminolevulinic acid	Not detected	Not detected	N-Carbamoyl-L-aspartic acid	10.56	9.72
2-Hydroxyisocaproic acid	5.18	10.57	Serine O-sulfate	Not detected	Not detected
4-Acetylbutyric acid	17.14	23.35	N-α-Benzoylarginine ethylester	Not detected	Not detected
Pipecolic acid	5.45	9.01	Cysteine-glutathione disulphide	Not detected	Not detected
2-Isopropylmalic acid	13.28	22.37	Phe-Phe	3.95	8.35
2,6-Diaminopimelic acid	10.94	24.77	Sorbitol 6-phosphate	Not detected	Not detected
2'-Deoxyinosine	7.25	14.36	Picolinic acid butyl ester	Not detected	Not detected
5'-Methylthioadenosine	3.79	15.15	4-Hydroxymethylimidazole	Not detected	Not detected
5'-Deoxyadenosine	4.45	15.96	Pyruvic acid	11.59	11.36
Trimethylamine	7.53	9.70	2-Oxoglutaric acid	12.19	6.13
Cadaverine	Saturated	Saturated	Deoxycholic acid	Not detected	Not detected
Norspermidine	Not detected	Not detected	Hyodeoxycholic acid	Not detected	Not detected
Trimethylamine N-oxide	Not detected	Not detected	Lithocholic acid	4.75	10.72
Putrescine	2.45	13.48	Ursodeoxycholic acid	Not detected	Not detected
N1,N8-Diacetylspermidine	8.21	21.54	Chenodeoxycholic acid	Not detected	Not detected
N-Acetyl-L-valine	8.22	19.81	3-(4-Hydroxyphenyl)propionic acid	5.37	3.47
N2-Phenylacetylglutamine	Not detected	Not detected	4-Hydroxyphenylacetic acid	15.41	9.50
γ-Butyrobetaine	Saturated	Saturated	2,3-Pyridinedicarboxylic acid	Not detected	Not detected
Taurine	10.65	10.90	Benzoic acid	5.32	2.07
N-ε-Acetyl-L-lysine	7.59	21.38	3-Hydroxybenzoic acid	Not detected	Not detected
N-γ-Ethyl-L-glutamine	8.04	15.75	3-Phenylpropionic acid	4.58	9.51
L-Cysteine S-sulfate	Saturated	Saturated	Malonic acid	4.52	10.55
Cycloleucine	10.47	8.57	Maleic acid	8.61	12.63
3-Indoxylsulfuric acid	Not detected	Not detected	Crotonic acid	8.95	12.62
Indole-3-acetic acid	8.40	21.24	Dodecanoic acid	Not detected	Not detected
Methyl sulfate	18.24	13.50	2-Hydroxypentanoic acid	2.76	9.02
Vanillylmandelic acid	Not detected	Not detected	2-Oxoisocaproic acid	14.51	20.50
1-Methyl-2-pyrrolidinone	35.79	27.25	Butyric acid	9.65	3.53
6-Methylaminopurine	14.15	13.56	Propionic acid	4.75	4.75
Proline betaine	5.56		Isobutyric acid	6.75	4.79
Thiamine	17.10	14.47	Valeric acid	3.81	4.52
Saccharic acid	17.01	12.57	Isovaleric acid	16.37	15.06
4-Oxopentanoic acid	13.07	10.28	2-Oxobutyric acid	12.84	11.82
Spermidine	3.12	4.47	2-Hydroxyglutaric acid	1.39	5.29
Spermine	118.97	116.82	3-Hydroxybutyric acid	7.74	4.31
2-Phenylethylamine	8.13	3.60	Prostaglandin E2	Not detected	Not detected

Table S7. Evaluation of sample stability under three conditions (autosampler, bench-top, and freeze–thaw). Stability (%) was calculated for each metabolite from the ratio of MRM peak areas in samples stored under each condition to those in the freshly prepared sample (Time 0) (n = 3).

Metabolites	Autosampler (24–36 h)	Bench-top (1.5 h)	Freeze–thaw (1 cycle)	Metabolites	Autosampler (24–36 h)	Bench-top (1.5 h)	Freeze–thaw (1 cycle)
Biotin	139.98	75.65	80.62	N1-Acetylspermine	111.76	101.83	105.20
Pyridoxine	108.08	78.72	117.00	N1-Acetylspermidine	108.21	103.06	101.59
Riboflavin	109.87	57.05	99.89	Tryptamine	99.52	99.35	98.67
5-Aminovaleric acid	Saturated	Saturated	Saturated	α-Methylbenzylamine	Not detected	Not detected	Not detected
2-Oxoisopentanoic acid	Not detected	Not detected	Not detected	N1,N12-Diacetylspermine	114.36	107.24	104.03
5-Aminolevulinic acid	Not detected	Not detected	Not detected	N-Carbamoyl-L-aspartic acid	115.46	103.59	101.25
2-Hydroxyisocaproic acid	99.10	97.39	98.11	Serine O-sulfate	Not detected	Not detected	Not detected
4-Acetylbutyric acid	94.39	96.61	88.31	N-α-Benzoylarginine ethylester	Not detected	Not detected	Not detected
Pipecolic acid	110.21	94.22	100.62	Cysteine-glutathione disulphide	Not detected	Not detected	Not detected
2-Isopropylmalic acid	102.27	106.80	116.31	Phe-Phe	109.01	104.19	95.46
2,6-Diaminopimelic acid	83.16	92.70	87.33	Sorbitol 6-phosphate	Not detected	Not detected	Not detected
2'-Deoxyinosine	96.51	90.31	100.55	Picolinic acid butyl ester	Not detected	Not detected	Not detected
5'-Methylthioadenosine	95.40	92.52	99.95	4-Hydroxymethylimidazole	Not detected	Not detected	Not detected
5'-Deoxyadenosine	94.45	100.60	101.20	Pyruvic acid	115.55	92.11	101.45
Trimethylamine	102.24	97.27	109.44	2-Oxoglutaric acid	118.94	59.16	66.59
Cadaverine	Saturated	Saturated	Saturated	Deoxycholic acid	Not detected	Not detected	Not detected
Norspermidine	Not detected	Not detected	Not detected	Hyodeoxycholic acid	Not detected	Not detected	Not detected
Trimethylamine N-oxide	Not detected	Not detected	Not detected	Lithocholic acid	90.93	95.79	95.41
Putrescine	98.84	100.89	102.04	Ursodeoxycholic acid	Not detected	Not detected	Not detected
N1,N8-Diacetylspermidine	89.50	95.96	100.51	Chenodeoxycholic acid	Not detected	Not detected	Not detected
N-Acetyl-L-valine	85.41	98.26	104.05	3-(4-Hydroxyphenyl)propionic acid	109.25	93.17	80.32
N2-Phenylacetylglutamine	Not detected	Not detected	Not detected	4-Hydroxyphenylacetic acid	132.50	78.88	63.22
γ-Butyrobetaine	Saturated	Saturated	Saturated	2,3-Pyridinedicarboxylic acid	Not detected	Not detected	Not detected
Taurine	117.60	88.49	112.25	Benzoic acid	112.92	91.05	89.12
N-ε-Acetyl-L-lysine	83.97	86.42	88.61	3-Hydroxybenzoic acid	Not detected	Not detected	Not detected
N-γ-Ethyl-L-glutamine	99.10	93.26	90.60	3-Phenylpropionic acid	94.86	109.51	113.21
L-Cysteine S-sulfate	Saturated	Saturated	Saturated	Malonic acid	90.47	90.85	101.56
Cycloleucine	119.95	85.77	111.01	Maleic acid	94.99	108.25	71.64
3-Indoxylsulfuric acid	Not detected	Not detected	Not detected	Crotonic acid	100.57	71.91	83.03
Indole-3-acetic acid	84.53	96.54	93.31	Dodecanoic acid	Not detected	Not detected	Not detected
Methyl sulfate	129.27	103.61	93.23	2-Hydroxypentanoic acid	95.24	108.25	107.78
Vanillylmandelic acid	Not detected	Not detected	Not detected	2-Oxoisocaproic acid	78.33	102.14	116.49
1-Methyl-2-pyrrolidinone	192.89	106.15	68.32	Butyric acid	120.97	91.48	81.10
6-Methylaminopurine	103.66	73.79	56.46	Propionic acid	110.47	99.46	93.44
Proline betaine	106.92	96.23	100.99	Isobutyric acid	103.58	84.37	88.89
Thiamine	76.22	95.07	100.63	Valeric acid	105.77	106.23	107.49
Saccharic acid	102.39	101.87	83.67	Isovaleric acid	120.12	85.13	80.17
4-Oxopentanoic acid	88.93	86.75	98.01	2-Oxobutyric acid	118.71	102.62	91.81
Spermidine	96.88	99.91	102.05	2-Hydroxyglutaric acid	105.07	89.49	81.44
Spermine	17.32	82.60	89.70	3-Hydroxybutyric acid	112.69	104.71	98.20
2-Phenylethylamine	101.26	128.55	103.23	Prostaglandin E2	Not detected	Not detected	Not detected

Table S8. Spike–recovery validation using intracellular metabolite extracts from gut microbiota. Spike–recovery was evaluated for intracellular metabolite extracts by spiking standards at three concentration levels: low (L, 0.01 μM), mid (M, 0.1 μM), and high (H, 1 μM). Recovery is reported as Recovery_L (%), Recovery_M (%), and Recovery_H (%) for each metabolite. Metabolites not detected in the unspiked sample were marked as "Not detected," and spike–recovery was not calculated for those metabolites.

Metabolites	Recovery_L (%)	Recovery_M (%)	Recovery_H (%)	Metabolites	Recovery_L (%)	Recovery_M (%)	Recovery_H (%)
Biotin	Not detected	Not detected	Not detected	N1-Acetylspermine	79.92	86.86	115.02
Pyridoxine	106.00	205.71	107.91	N1-Acetylspermidine	109.37	116.55	97.70
Riboflavin	113.50	138.72	104.46	Tryptamine	51.51	66.27	86.37
5-Aminovaleric acid	-1945.58	85.47	91.34	α-Methylbenzylamine	Not detected	Not detected	Not detected
2-Oxoisopentanoic acid	Not detected	Not detected	Not detected	N1,N12-Diacetylspermine	129.18	151.88	111.11
5-Aminolevulinic acid	Not detected	Not detected	Not detected	N-Carbamoyl-L-aspartic acid	100.22	51.79	42.75
2-Hydroxyisocaproic acid	-431.94	104.95	90.94	Serine O-sulfate	Not detected	Not detected	Not detected
4-Acetylbutyric acid	Not detected	Not detected	Not detected	N-α-Benzoylarginine ethylester	51.29	64.17	90.30
Pipecolic acid	95.65	210.29	106.36	Cysteine-glutathione disulphide	Not detected	Not detected	Not detected
2-Isopropylmalic acid	196.84	204.56	118.83	Phe-Phe	96.92	97.15	93.99
2,6-Diaminopimelic acid	81.67	188.17	94.96	Sorbitol 6-phosphate	Not detected	Not detected	Not detected
2'-Deoxyinosine	79.23	177.20	99.42	Picolinic acid butyl ester	Not detected	Not detected	Not detected
5'-Methylthioadenosine	106.29	182.49	102.83	4-Hydroxymethylimidazole	19.00	22.16	26.62
5'-Deoxyadenosine	91.90	169.13	96.29	Pyruvic acid	41.65	113.01	117.69
Trimethylamine	Not detected	Not detected	Not detected	2-Oxoglutaric acid	Not detected	Not detected	Not detected
Cadaverine	100.58	182.99	63.74	Deoxycholic acid	101.32	103.79	100.35
Norspermidine	Not detected	Not detected	Not detected	Hyodeoxycholic acid	Not detected	Not detected	Not detected
Trimethylamine N-oxide	Not detected	Not detected	Not detected	Lithocholic acid	107.41	108.73	103.53
Putrescine	232.30	199.79	92.67	Ursodeoxycholic acid	Not detected	Not detected	Not detected
N1,N8-Diacetylspermidine	101.07	185.30	100.61	Chenodeoxycholic acid	Not detected	Not detected	Not detected
N-Acetyl-L-valine	-33.41	188.47	106.92	3-(4-Hydroxyphenyl)propionic acid	87.92	79.86	79.38
N2-Phenylacetylglutamine	117.75	246.34	117.59	4-Hydroxyphenylacetic acid	52.85	58.39	61.70
γ-Butyrobetaine	189.95	144.50	92.39	2,3-Pyridinedicarboxylic acid	Not detected	Not detected	Not detected
Taurine	Not detected	Not detected	Not detected	Benzoic acid	24.16	68.57	84.17
N-ε-Acetyl-L-lysine	101.39	189.69	99.13	3-Hydroxybenzoic acid	Not detected	Not detected	Not detected
N-γ-Ethyl-L-glutamine	Not detected	Not detected	Not detected	3-Phenylpropionic acid	1371.50	118.17	108.26
L-Cysteine S-sulfate	Not detected	Not detected	Not detected	Malonic acid	113.00	114.34	116.36
Cycloleucine	136.37	212.26	113.21	Maleic acid	78.46	94.48	91.58
3-Indoxylsulfuric acid	Not detected	Not detected	Not detected	Crotonic acid	51.48	85.50	83.41
Indole-3-acetic acid	104.26	189.83	102.65	Dodecanoic acid	Not detected	Not detected	Not detected
Methyl sulfate	122.33	100.42	90.88	2-Hydroxypentanoic acid	233.73	109.55	99.74
Vanillylmandelic acid	Not detected	Not detected	Not detected	2-Oxoisocaproic acid	87.37	84.84	90.40
1-Methyl-2-pyrrolidinone	91.14	151.96	102.57	Butyric acid	32.74	83.68	75.31
6-Methylaminopurine	87.63	147.50	79.45	Propionic acid	21.64	69.03	75.73
Proline betaine	87.32	104.50	139.68	Isobutyric acid	48.02	79.32	79.14
Thiamine	110.11	105.13	87.83	Valeric acid	67.65	99.29	95.64
Saccharic acid	Not detected	Not detected	Not detected	Isovaleric acid	49.93	84.45	81.46
4-Oxopentanoic acid	74.16	74.25	90.13	2-Oxobutyric acid	91.57	99.93	92.61
Spermidine	28.11	69.45	102.74	2-Hydroxyglutaric acid	77.49	89.21	87.99
Spermine	Not detected	Not detected	Not detected	3-Hydroxybutyric acid	115.42	92.93	85.50
2-Phenylethylamine	78.31	91.36	97.20	Prostaglandin E2	Not detected	Not detected	Not detected

Table S9. Matrix effect evaluation using slope comparison. Matrix effect (%) was calculated for each metabolite as (slope_matrix/slope_solvent) × 100 using calibration curves at 0.01 µM, 0.1 µM, and 1 µM. “Not evaluable” indicates insufficient quantifiable calibration points for reliable slope comparison between solvent and matrix.

Metabolites	Matrix effect (%)	Metabolites	Matrix effect (%)
Biotin	93.4	N1-Acetylspermine	83.9
Pyridoxine	92.8	N1-Acetylspermidine	105.5
Riboflavin	94.8	Tryptamine	113.5
5-Aminovaleric acid	89.8	α-Methylbenzylamine	133.2
2-Oxoisopentanoic acid	Not evaluable	N1,N12-Diacetylspermine	92.7
5-Aminolevulinic acid	Not evaluable	N-Carbamoyl-L-aspartic acid	239.2
2-Hydroxyisocaproic acid	105.8	Serine O-sulfate	53.2
4-Acetylbutyric acid	95.1	N-α-Benzoylarginine ethylester	102.7
Pipecolic acid	93.9	Cysteine-glutathione disulphide	Not evaluable
2-Isopropylmalic acid	84.2	Phe-Phe	107.1
2,6-Diaminopimelic acid	105.5	Sorbitol 6-phosphate	122.8
2'-Deoxyinosine	98.9	Picolinic acid butyl ester	101.6
5'-Methylthioadenosine	96.8	4-Hydroxymethylimidazole	369.5
5'-Deoxyadenosine	104.0	Pyruvic acid	83.7
Trimethylamine	101.3	2-Oxoglutaric acid	173.7
Cadaverine	164.2	Deoxycholic acid	99.8
Norspermidine	109.0	Hyodeoxycholic acid	98.9
Trimethylamine N-oxide	95.8	Lithocholic acid	96.8
Putrescine	109.9	Ursodeoxycholic acid	86.5
N1,N8-Diacetylspermidine	99.4	Chenodeoxycholic acid	94.0
N-Acetyl-L-valine	92.5	3-(4-Hydroxyphenyl)propionic acid	126.1
N2-Phenylacetylglutamine	85.8	4-Hydroxyphenylacetic acid	161.5
γ-Butyrobetaine	108.5	2,3-Pyridinedicarboxylic acid	95.3
Taurine	92.8	Benzoic acid	116.1
N-ε-Acetyl-L-lysine	101.2	3-Hydroxybenzoic acid	138.7
N-γ-Ethyl-L-glutamine	96.4	3-Phenylpropionic acid	98.4
L-Cysteine S-sulfate	70.0	Malonic acid	85.9
Cycloleucine	88.8	Maleic acid	109.2
3-Indoxylsulfuric acid	Not evaluable	Crotonic acid	119.6
Indole-3-acetic acid	97.8	Dodecanoic acid	Not evaluable
Methyl sulfate	111.2	2-Hydroxypentanoic acid	101.3
Vanillylmandelic acid	Not evaluable	2-Oxoisocaproic acid	110.1
1-Methyl-2-pyrrolidinone	96.4	Butyric acid	132.0
6-Methylaminopurine	125.5	Propionic acid	129.0
Proline betaine	69.5	Isobutyric acid	126.0
Thiamine	118.9	Valeric acid	104.5
Saccharic acid	Not evaluable	Isovaleric acid	122.5
4-Oxopentanoic acid	109.2	2-Oxobutyric acid	108.4
Spermidine	92.5	2-Hydroxyglutaric acid	113.6
Spermine	112.0	3-Hydroxybutyric acid	117.6
2-Phenylethylamine	102.1	Prostaglandin E2	70.2

Table S10. Metabolite order used in Figure 6 (heatmap) and detection status.

Order in Figure 6 (left to right)	Metabolites	Status
1	4-Hydroxyproline	Detected
2	3-Dehydroquinic acid	Detected
3	Dopamine	Detected
4	Threonine	Detected
5	2,6-Diaminopimelic acid	Detected
6	Malonic acid	Detected
7	5'-Deoxyadenosine	Detected
8	Dopa	Detected
9	Creatine	Detected
10	Cycloleucine	Detected
11	Cystine	Detected
12	2-Hydroxypentanoic acid	Detected
13	2-Oxoglutaric acid	Detected
14	3-Phenylpropionic acid	Detected
15	Cytidine	Detected
16	Serotonin	Detected
17	γ-Butyrobetaine	Detected
18	Phenyllactic acid	Detected
19	Biotin	Detected
20	4-Hydroxymethylimidazole	Detected
21	N-Acetyl-L-valine	Detected
22	Cadaverine	Detected
23	Cysteine	Detected
24	Putrescine	Detected
25	4-Aminobutyric acid	Detected
26	5-Aminovaleric acid	Detected
27	4-Aminobenzoic acid	Detected
28	Tyramine	Detected
29	Pantothenic acid	Detected
30	Picolinic acid butyl ester	Detected
31	Anthranilic acid	Detected
32	Pyridoxal 5'-phosphoric acid	Detected
33	Indole	Detected
34	N1-Acetylspermine	Detected
35	Adenosine 3',5'-cyclic phosphoric acid	Detected
36	Xanthine	Detected
37	Indole-3-acetic acid	Detected

Table S10 (continued)

Order in Figure 6 (left to right)	Metabolites	Status
38	3-Hydroxybenzoic acid	Detected
39	Benzoic acid	Detected
40	6-Methylaminopurine	Detected
41	4-Acetylbutyric acid	Detected
42	Thymidine 5'-monophosphoric acid	Detected
43	N-γ-Ethyl-L-glutamine	Detected
44	Deoxycholic acid	Detected
45	Lithocholic acid	Detected
46	Cytidine-5'-monophosphoric acid	Detected
47	Vanillic acid	Detected
48	2-Phenylethylamine	Detected
49	N-Carbamoyl-L-aspartic acid	Detected
50	Isobutyric acid	Detected
51	Butyric acid	Detected
52	Propionic acid	Detected
53	Valeric acid	Detected
55	Isovaleric acid	Detected
56	Cytosine	Detected
57	N-α-Benzoylarginine ethylester	Detected
58	α-Methylbenzylamine	Detected
59	Proline betaine	Detected
60	Crotonic acid	Detected
61	Riboflavin	Detected
62	Flavin mononucleotide	Detected
63	5-Phosphomevalonic acid	Detected
64	4-Oxopentanoic acid	Detected
65	1-Methyl-2-pyrrolidinone	Detected
66	Salicylic acid	Detected
67	N1,N12-Diacetylspermine	Detected
68	Flavin adenine dinucleotide	Detected
69	4-Hydroxybenzoic acid	Detected
70	Guanosine 3',5'-cyclic monophosphoric acid	Detected
71	Thymine	Detected
72	3-Hydroxybutyric acid	Detected
73	Acetylcarnitine	Detected
74	Mevalonic acid	Detected

Table S10 (continued)

Order in Figure 6 (left to right)	Metabolites	Status
75	Pyridoxine	Detected
76	Guanosine	Detected
77	Tryptamine	Detected
78	N1,N8-Diacetylspermidine	Detected
79	Thymidine	Detected
80	Kynurenine	Detected
81	Uric acid	Detected
82	Adenine	Detected
83	Guanine	Detected
84	Creatinine	Detected
85	Citrulline	Detected
86	Urocanic acid	Detected
87	Histamine	Detected
88	Tyrosine	Detected
89	Oxidized glutathione	Detected
90	Ornithine	Detected
91	Proline	Detected
92	Glutathione	Detected
93	Tryptophan	Detected
94	Glutamic acid	Detected
95	Ophthalmic acid	Detected
96	Serine O-sulfate	Detected
97	Aspartic acid	Detected
98	Argininosuccinic acid	Detected
99	Histidinol	Detected
100	Phe-Phe	Detected
101	Ergothioneine	Detected
102	Glutamine	Detected
103	Histidine	Detected
104	Phenylpyruvic acid	Detected
105	Methionine	Detected
106	Malic acid	Detected
107	Methionine sulfoxide	Detected
108	2-C-Methyl-D-erythritol 4-phosphoric acid	Detected
109	Protocatechuic acid	Detected
110	Carnosine	Detected

Table S10 (continued)

Order in Figure 6 (left to right)	Metabolites	Status
111	3-Phosphoshikimic acid	Detected
112	4-Hydroxyphenylacetic acid	Detected
113	Choline	Detected
114	Succinic acid	Detected
115	S-Adenosylmethionine	Detected
116	Nicotinamide adenine dinucleotide	Detected
117	Nicotinamide	Detected
118	3-(4-Hydroxyphenyl)propionic acid	Detected
119	Arginine	Detected
120	N1-Acetylspermidine	Detected
121	Lysine	Detected
122	5'-Methylthioadenosine	Detected
123	2-Hydroxyisocaproic acid	Detected
124	Saccharic acid	Detected
125	Guanosine 5'-monophosphoric acid	Detected
126	Valine	Detected
127	Phenylalanine	Detected
128	Asparagine	Detected
129	Isoleucine	Detected
130	Leucine	Detected
131	2-Aminobutyric acid	Detected
132	Uracil	Detected
133	Serine	Detected
134	Fumaric acid	Detected
135	Cystathionine	Detected
136	Carnitine	Detected
137	Citicoline	Detected
138	Nicotinic acid	Detected
139	Pyruvic acid	Detected
140	Asymmetric dimethylarginine	Detected
141	Alanine	Detected
142	Uridine	Detected
143	Adenosine	Detected
144	2-Oxobutyric acid	Detected
145	2-Oxoisocaproic acid	Detected
146	Maleic acid	Detected

Table S10 (continued)

Order in Figure 6 (left to right)	Metabolites	Status
147	Glycine	Detected
148	Adenylsuccinic acid	Detected
149	Citric acid	Detected
150	2-Isopropylmalic acid	Detected
151	Inosine	Detected
152	Hypoxanthine	Detected
153	S-Adenosylhomocysteine	Detected
154	2-Hydroxyglutaric acid	Detected
155	2,3-Pyridinedicarboxylic acid	Detected
156	Trimethylamine	Detected
157	Adenosine 5'-monophosphoric acid	Detected
158	Spermine	Detected
159	Spermidine	Detected
160	Cysteine-glutathione disulphide	Not detected
161	Sorbitol 6-phosphoate	Not detected
162	Chenodeoxycholic acid	Not detected
163	Dodecanoic acid	Not detected
164	Hyodeoxycholic acid	Not detected
165	Prostaglandin E2	Not detected
166	Ursodeoxycholic acid	Not detected
167	5-Diphosphomevalonic acid	Not detected
168	Symmetric dimethylarginine	Not detected
169	5-Glutamylcysteine	Not detected
170	Acetylcholine	Not detected
171	Aconitic acid	Not detected
172	Adrenaline	Not detected
173	Allantoin	Not detected
174	Cytidine 3',5'-cyclic monophosphoric acid	Not detected
175	Cholic acid	Not detected
176	Cysteamine	Not detected
177	Dimethylglycine	Not detected
178	Isocitric acid	Not detected
179	Taurocholic acid	Not detected
180	Homocysteine	Not detected
181	Homocystine	Not detected
182	Noradrenaline	Not detected

Table S10 (continued)

Order in Figure 6 (left to right)	Metabolites	Status
183	Orotic acid	Not detected
184	3-Dehydroshikimic acid	Not detected
185	AICAR	Not detected
186	Chorismic acid	Not detected
187	1-Deoxy-D-xylulose 5-phosphoric acid	Not detected
188	Shikimic acid	Not detected
189	2'-Deoxyinosine	Not detected
190	2-Oxoisopentanoic acid	Not detected
191	Vanillylmandelic acid	Not detected
192	5-Aminolevulinic acid	Not detected
193	L-Cysteine S-sulfate	Not detected
194	Folic acid	Not detected
195	3-Indoxylsulfuric acid	Not detected
196	Methyl sulfate	Not detected
197	N-ε-Acetyl-L-lysine	Not detected
198	Norspermidine	Not detected
199	4-Coumaric acid	Not detected
200	N2-Phenylacetylglutamine	Not detected
201	Pipecolic acid	Not detected
202	Taurine	Not detected
203	Trimethylamine N-oxide	Not detected
204	3,4-Dihydroxyphenylacetaldehyde	Not detected
205	3,4-Dihydroxyphenylacetic acid	Not detected
206	Hydroxytyrosol	Not detected
207	Methyldopa	Not detected
208	4-Aminophenylalanine	Not detected
209	4-Aminophenylpyruvic acid	Not detected
210	Caffeic acid	Not detected
211	Catechol	Not detected
212	Ferulic acid	Not detected
213	Protocatechualdehyde	Not detected
214	Sinapic acid	Not detected
215	Vanillin	Not detected

Table S11. Raw p-values and Benjamini–Hochberg FDR-adjusted p-values for differential metabolites (Figure 7). Raw p-values and Benjamini–Hochberg FDR-adjusted p-values were reported for the intracellular and extracellular metabolite comparisons shown in Figure 7. FDR correction was applied separately within each dataset (intracellular and extracellular).

Intracellular

Metabolites	Raw p-value	Adjusted p-value
Anthranilic acid	0.000235	0.035919
N-Carbamoyl-L-aspartic acid	0.000683	0.052257
4-Hydroxybenzoic acid	0.001706	0.061229
Creatinine	0.001983	0.061229
Xanthine	0.002001	0.061229
Proline betaine	0.002451	0.062503
Nicotinic acid	0.003298	0.068857
Saccharic acid	0.003600	0.068857
Asparagine	0.004186	0.071167
4-Acetylbutyric acid	0.007341	0.111598
Uracil	0.008638	0.111598
Valine	0.008753	0.111598
Isoleucine	0.009877	0.114594
Lysine	0.011392	0.114594
N-Acetyl-L-valine	0.011914	0.114594
3-Phenylpropionic acid	0.011984	0.114594
Protocatechuic acid	0.014625	0.130321
Picolinic acid butyl ester	0.015332	0.130321
Asymmetric dimethylarginine	0.022252	0.175872
Spermine	0.022990	0.175872
Indole-3-acetic acid	0.027136	0.191724
2-Oxoisocaproic acid	0.027568	0.191724
N-γ-Ethyl-L-glutamine	0.030709	0.199363
2-Hydroxyglutaric acid	0.033212	0.199363
3-(4-Hydroxyphenyl)propionic acid	0.034600	0.199363

Table S11 (continued)

Metabolites	Raw p-value	Adjusted p-value
1-Methyl-2-pyrrolidinone	0.035182	0.199363
N1-Acetylspermine	0.037147	0.202984
2-Isopropylmalic acid	0.048120	0.253630
Maleic acid	0.050489	0.253630
Adenosine 5'-monophosphoric acid	0.051389	0.253630
Pyridoxal 5'-phosphoric acid	0.055361	0.262628
Guanosine 5'-monophosphoric acid	0.056645	0.262628
Thymidine 5'-monophosphoric acid	0.060406	0.271827
Cytosine	0.067226	0.275950
Salicylic acid	0.068373	0.275950
Histidine	0.068513	0.275950
Adenosine	0.068537	0.275950
Leucine	0.075380	0.283891
Methionine sulfoxide	0.076046	0.283891
Guanosine	0.076075	0.283891
Dopa	0.084653	0.296829
Spermidine	0.088341	0.296829
Vanillic acid	0.089518	0.296829
Uridine	0.091563	0.296829
2-Phenylethylamine	0.092243	0.296829
Citric acid	0.092483	0.296829
Kynurenine	0.093123	0.296829
α-Methylbenzylamine	0.101634	0.311134
Pyruvic acid	0.101678	0.311134
Carnitine	0.109112	0.319983
Citicoline	0.109112	0.319983
4-Hydroxymethylimidazole	0.110844	0.319983
N-α-Benzoylarginine ethylester	0.121084	0.340496
Adenosine 3',5'-cyclic phosphoric acid	0.122401	0.340496
Flavin adenine dinucleotide	0.127355	0.347952
Trimethylamine	0.133576	0.358545
2-Aminobutyric acid	0.137003	0.361406
2,3-Pyridinedicarboxylic acid	0.141633	0.367285

Table S11 (continued)

Metabolites	Raw p-value	Adjusted p-value
Inosine	0.151506	0.382534
Crotonic acid	0.152514	0.382534
5'-Methylthioadenosine	0.163353	0.403114
Citrulline	0.169587	0.404697
Riboflavin	0.174250	0.404697
Phenylpyruvic acid	0.174334	0.404697
Arginine	0.174575	0.404697
Flavin mononucleotide	0.177569	0.405494
Indole	0.181245	0.406545
4-Oxopentanoic acid	0.187328	0.406545
Histamine	0.188617	0.406545
Alanine	0.188658	0.406545
2-Oxobutyric acid	0.196583	0.417739
Ornithine	0.203688	0.426908
3-Hydroxybenzoic acid	0.209076	0.427460
Cystathionine	0.210905	0.427460
Lithocholic acid	0.216080	0.427460
Serine	0.216604	0.427460
Isobutyric acid	0.217921	0.427460
Isovaleric acid	0.225895	0.431555
Cytidine-5'-monophosphoric acid	0.232333	0.431555
Methionine	0.235488	0.431555
Deoxycholic acid	0.239782	0.431555
2-C-Methyl-D-erythritol 4-phosphoric acid	0.240246	0.431555
Glutamine	0.242028	0.431555
Thymine	0.244444	0.431555
Malic acid	0.247433	0.431555
S-Adenosylmethionine	0.248116	0.431555
N1,N12-Diacetylspermine	0.248215	0.431555
Butyric acid	0.251766	0.432811
Valeric acid	0.263772	0.448412
Propionic acid	0.272092	0.457473
Dopamine	0.277839	0.462059

Table S11 (continued)

Metabolites	Raw p-value	Adjusted p-value
3-Dehydroquinic acid	0.285205	0.463255
Phenyllactic acid	0.288544	0.463255
Threonine	0.290477	0.463255
2-Hydroxypentanoic acid	0.290670	0.463255
Thiamine	0.294989	0.465292
Phenylalanine	0.310709	0.484071
Tyrosine	0.313222	0.484071
Benzoic acid	0.321946	0.490836
4-Hydroxyproline	0.333188	0.490836
3-Phosphoshikimic acid	0.334166	0.490836
2,6-Diaminopimelic acid	0.334731	0.490836
Adenylsuccinic acid	0.335536	0.490836
Glutamic acid	0.336848	0.490836
Proline	0.357187	0.514917
Pyridoxine	0.360105	0.514917
Malonic acid	0.424290	0.601078
Fumaric acid	0.431272	0.603672
5-Phosphomevalonic acid	0.434013	0.603672
Nicotinamide adenine dinucleotide	0.443242	0.610955
4-Aminobenzoic acid	0.457442	0.624899
Hypoxanthine	0.486809	0.658555
Tryptophan	0.490688	0.658555
Carnosine	0.508371	0.667075
Putrescine	0.511028	0.667075
Cystine	0.511794	0.667075
Tyramine	0.517476	0.667075
Biotin	0.520773	0.667075
5-Aminovaleric acid	0.529780	0.667075
2-Oxoglutaric acid	0.531275	0.667075
Glycine	0.534316	0.667075
5'-Deoxyadenosine	0.537478	0.667075
Aspartic acid	0.541421	0.667075
Adenine	0.544996	0.667075
Pantothenic acid	0.570193	0.685072

Table S11 (continued)

Metabolites	Raw p-value	Adjusted p-value
Choline	0.575834	0.685072
4-Aminobutyric acid	0.580929	0.685072
6-Methylaminopurine	0.582069	0.685072
Succinic acid	0.583923	0.685072
γ-Butyrobetaine	0.586565	0.685072
Cycloleucine	0.608761	0.704903
Phe-Phe	0.612981	0.704903
4-Hydroxyphenylacetic acid	0.619591	0.704903
N1-Acetylspermidine	0.621974	0.704903
Serotonin	0.635908	0.711666
Uric acid	0.637243	0.711666
Guanine	0.654068	0.725162
Mevalonic acid	0.723790	0.796690
Guanosine 3',5'-cyclic monophosphoric acid	0.748932	0.818475
Cysteine	0.767273	0.832572
Ergothioneine	0.829989	0.891101
N1,N8-Diacetylspermidine	0.832859	0.891101
Acetylcarnitine	0.848310	0.897482
Cytidine	0.854940	0.897482
Urocanic acid	0.860304	0.897482
Nicotinamide	0.862286	0.897482
S-Adenosylhomocysteine	0.875199	0.904766
Tryptamine	0.900016	0.924178
Ophthalmic acid	0.912753	0.931008
3-Hydroxybutyric acid	0.926653	0.938927
Creatine	0.943237	0.949442
Cadaverine	0.980251	0.980251
Cysteine-glutathione disulphide	Not detected	Not detected
Serine O-sulfate	Not detected	Not detected
Sorbitol 6-phosphate	Not detected	Not detected
Chenodeoxycholic acid	Not detected	Not detected
Dodecanoic acid	Not detected	Not detected
Hyodeoxycholic acid	Not detected	Not detected

Table S11 (continued)

Metabolites	Raw p-value	Adjusted p-value
Prostaglandin E2	Not detected	Not detected
Ursodeoxycholic acid	Not detected	Not detected
Argininosuccinic acid	Not detected	Not detected
Oxidized glutathione	Not detected	Not detected
Symmetric dimethylarginine	Not detected	Not detected
5-Glutamylcysteine	Not detected	Not detected
Acetylcholine	Not detected	Not detected
Aconitic acid	Not detected	Not detected
Adrenaline	Not detected	Not detected
Allantoin	Not detected	Not detected
Cytidine 3',5'-cyclic monophosphoric acid	Not detected	Not detected
Cholic acid	Not detected	Not detected
Cysteamine	Not detected	Not detected
Dimethylglycine	Not detected	Not detected
Glutathione	Not detected	Not detected
Isocitric acid	Not detected	Not detected
Taurocholic acid	Not detected	Not detected
Homocysteine	Not detected	Not detected
Homocystine	Not detected	Not detected
Noradrenaline	Not detected	Not detected
Orotic acid	Not detected	Not detected
3-Dehydroshikimic acid	Not detected	Not detected
AICAR	Not detected	Not detected
Chorismic acid	Not detected	Not detected
1-Deoxy-D-xylulose 5-phosphoric acid	Not detected	Not detected
Histidinol	Not detected	Not detected
Shikimic acid	Not detected	Not detected
2'-Deoxyinosine	Not detected	Not detected
2-Oxoisopentanoic acid	Not detected	Not detected
Vanillylmandelic acid	Not detected	Not detected
5-Aminolevulinic acid	Not detected	Not detected
L-Cysteine S-sulfate	Not detected	Not detected
Folic acid	Not detected	Not detected

Table S11 (continued)

Metabolites	Raw p-value	Adjusted p-value
3-Indoxylsulfuric acid	Not detected	Not detected
Methyl sulfate	Not detected	Not detected
N-ε-Acetyl-L-lysine	Not detected	Not detected
Norspermidine	Not detected	Not detected
4-Coumaric acid	Not detected	Not detected
N2-Phenylacetylglutamine	Not detected	Not detected
Pipecolic acid	Not detected	Not detected
Taurine	Not detected	Not detected
Trimethylamine N-oxide	Not detected	Not detected
3,4-Dihydroxyphenylacetaldehyde	Not detected	Not detected
3,4-Dihydroxyphenylacetic acid	Not detected	Not detected
Hydroxytyrosol	Not detected	Not detected
Methyldopa	Not detected	Not detected
4-Aminophenylalanine	Not detected	Not detected
4-Aminophenylpyruvic acid	Not detected	Not detected
Caffeic acid	Not detected	Not detected
Catechol	Not detected	Not detected
Ferulic acid	Not detected	Not detected
Protocatechualdehyde	Not detected	Not detected
Sinapic acid	Not detected	Not detected
Vanillin	Not detected	Not detected
2-Hydroxyisocaproic acid	Not detected	Not detected
5-Diphosphomevalonic acid	Not detected	Not detected

Extracellular

Metabolites	Raw p-value	Adjusted p-value
Valine	0.000002	0.000329
N-Acetyl-L-valine	0.000145	0.011130
Dopa	0.000227	0.011565
Leucine	0.000644	0.024634

Table S11 (continued)

Metabolites	Raw p-value	Adjusted p-value
3-(4-Hydroxyphenyl)propionic acid	0.000889	0.027210
Isoleucine	0.001115	0.028421
Isobutyric acid	0.001944	0.038316
Anthranilic acid	0.002003	0.038316
Phenylalanine	0.006978	0.118620
4-Hydroxyphenylacetic acid	0.007799	0.119319
3-Phenylpropionic acid	0.010223	0.142196
Valeric acid	0.014555	0.185576
2,6-Diaminopimelic acid	0.016733	0.196940
Cycloleucine	0.019757	0.215912
γ-Butyrobetaine	0.031047	0.301410
Adenosine	0.032555	0.301410
Tyrosine	0.033490	0.301410
Asymmetric dimethylarginine	0.035480	0.301582
Arginine	0.047701	0.384119
2-Aminobutyric acid	0.054215	0.414748
Alanine	0.073766	0.524623
Crotonic acid	0.075918	0.524623
2-Oxoglutaric acid	0.078865	0.524623
Glutamine	0.101277	0.619818
4-Hydroxyproline	0.110234	0.648683
Histidine	0.118311	0.662758
N-ε-Acetyl-L-lysine	0.121289	0.662758
Guanosine	0.146878	0.760977
Nicotinamide	0.149211	0.760977
Spermidine	0.175517	0.818132
Proline	0.177457	0.818132
Methionine sulfoxide	0.179719	0.818132
Creatinine	0.181807	0.818132
Cytidine	0.188885	0.825699
1-Methyl-2-pyrrolidinone	0.239694	1.018698
Proline betaine	0.256514	1.050693
Asparagine	0.260957	1.050693

Table S11 (continued)

Metabolites	Raw p-value	Adjusted p-value
4-Aminobenzoic acid	0.269021	1.055389
Butyric acid	0.302174	1.122284
N1,N12-Diacetylspermine	0.308011	1.122284
N1,N8-Diacetylspermidine	0.308078	1.122284
Tryptamine	0.326681	1.161223
2-Hydroxyglutaric acid	0.335608	1.161223
Picolinic acid butyl ester	0.341536	1.161223
Ornithine	0.372448	1.238793
Kynurenine	0.394044	1.265537
Malonic acid	0.409592	1.265537
2-Oxobutyric acid	0.428909	1.265537
Threonine	0.432162	1.265537
4-Aminobutyric acid	0.433480	1.265537
α-Methylbenzylamine	0.442149	1.265537
4-Oxopentanoic acid	0.442365	1.265537
Isovaleric acid	0.446660	1.265537
Adenine	0.462413	1.286349
Benzoic acid	0.502110	1.371835
Indole	0.531512	1.426690
Vanillin	0.585602	1.459234
4-Hydroxybenzoic acid	0.594926	1.459234
Propionic acid	0.605761	1.459234
Cadaverine	0.613232	1.459234
Dopamine	0.614588	1.459234
Serine	0.618927	1.459234
2-Oxoisocaproic acid	0.619178	1.459234
3-Hydroxybenzoic acid	0.619936	1.459234
Spermine	0.636433	1.475367
2-Hydroxypentanoic acid	0.686537	1.550130
2-Phenylethylamine	0.692503	1.550130
Tryptophan	0.706814	1.550130
Lysine	0.709210	1.550130
N1-Acetylspermidine	0.720323	1.552246

Table S11 (continued)

Metabolites	Raw p-value	Adjusted p-value
Glutamic acid	0.738099	1.568461
Tyramine	0.757589	1.587823
Pyruvic acid	0.782128	1.611153
N-α-Benzoylarginine ethylester	0.789781	1.611153
4-Hydroxymethylimidazole	0.841339	1.682196
Citrulline	0.846595	1.682196
Glycine	0.936945	1.786704
Trimethylamine	0.943380	1.786704
3-Hydroxybutyric acid	0.948791	1.786704
Phe-Phe	0.954620	1.786704
Aspartic acid	0.957580	1.786704
Putrescine	0.983065	1.812155
5-Glutamylcysteine	Not detected	Not detected
Acetylcarnitine	Not detected	Not detected
Acetylcholine	Not detected	Not detected
Aconitic acid	Not detected	Not detected
Adenylsuccinic acid	Not detected	Not detected
Adrenaline	Not detected	Not detected
Allantoin	Not detected	Not detected
Adenosine 5'-monophosphoric acid	Not detected	Not detected
Argininosuccinic acid	Not detected	Not detected
Adenosine 3',5'-cyclic phosphoric acid	Not detected	Not detected
Carnitine	Not detected	Not detected
Carnosine	Not detected	Not detected
Cytidine 3',5'-cyclic monophosphoric acid	Not detected	Not detected
Guanosine 3',5'-cyclic monophosphoric acid	Not detected	Not detected
Cholic acid	Not detected	Not detected
Choline	Not detected	Not detected
Citicoline	Not detected	Not detected
Citric acid	Not detected	Not detected
Cytidine-5'-monophosphoric acid	Not detected	Not detected
Creatine	Not detected	Not detected
Cysteine	Not detected	Not detected
Cysteamine	Not detected	Not detected

Table S11 (continued)

Metabolites	Raw p-value	Adjusted p-value
Cytosine	Not detected	Not detected
Dimethylglycine	Not detected	Not detected
Flavin adenine dinucleotide	Not detected	Not detected
Flavin mononucleotide	Not detected	Not detected
Fumaric acid	Not detected	Not detected
Glutathione	Not detected	Not detected
Guanosine 5'-monophosphoric acid	Not detected	Not detected
Histamine	Not detected	Not detected
Inosine	Not detected	Not detected
Isocitric acid	Not detected	Not detected
Malic acid	Not detected	Not detected
Methionine	Not detected	Not detected
Nicotinamide adenine dinucleotide	Not detected	Not detected
Nicotinic acid	Not detected	Not detected
Ophthalmic acid	Not detected	Not detected
Oxidized glutathione	Not detected	Not detected
Pantothenic acid	Not detected	Not detected
S-Adenosylmethionine	Not detected	Not detected
Serotonin	Not detected	Not detected
Succinic acid	Not detected	Not detected
Symmetric dimethylarginine	Not detected	Not detected
Taurocholic acid	Not detected	Not detected
Thymidine	Not detected	Not detected
Thymidine 5'-monophosphoric acid	Not detected	Not detected
Uridine	Not detected	Not detected
Cystathionine	Not detected	Not detected
Cystine	Not detected	Not detected
Guanine	Not detected	Not detected
Homocysteine	Not detected	Not detected
Homocystine	Not detected	Not detected
Hypoxanthine	Not detected	Not detected
Noradrenaline	Not detected	Not detected
Orotic acid	Not detected	Not detected

Table S11 (continued)

Metabolites	Raw p-value	Adjusted p-value
S-Adenosylhomocysteine	Not detected	Not detected
Thymine	Not detected	Not detected
Uracil	Not detected	Not detected
Uric acid	Not detected	Not detected
Xanthine	Not detected	Not detected
3-Dehydroquinic acid	Not detected	Not detected
3-Dehydroshikimic acid	Not detected	Not detected
3-Phosphoshikimic acid	Not detected	Not detected
5-Phosphomevalonic acid	Not detected	Not detected
AICAR	Not detected	Not detected
Chorismic acid	Not detected	Not detected
1-Deoxy-D-xylulose 5-phosphoric acid	Not detected	Not detected
Ergothioneine	Not detected	Not detected
Histidinol	Not detected	Not detected
2-C-Methyl-D-erythritol 4-phosphoric acid	Not detected	Not detected
Mevalonic acid	Not detected	Not detected
Phenyllactic acid	Not detected	Not detected
Phenylpyruvic acid	Not detected	Not detected
Shikimic acid	Not detected	Not detected
Urocanic acid	Not detected	Not detected
2'-Deoxyinosine	Not detected	Not detected
2-Hydroxyisocaproic acid	Not detected	Not detected
2-Isopropylmalic acid	Not detected	Not detected
2-Oxoisopentanoic acid	Not detected	Not detected
Indole-3-acetic acid	Not detected	Not detected
4-Acetylbutyric acid	Not detected	Not detected
Vanillylmandelic acid	Not detected	Not detected
5-Aminolevulinic acid	Not detected	Not detected
5'-Deoxyadenosine	Not detected	Not detected
5'-Methylthioadenosine	Not detected	Not detected
6-Methylaminopurine	Not detected	Not detected
Biotin	Not detected	Not detected
L-Cysteine S-sulfate	Not detected	Not detected

Table S11 (continued)

Metabolites	Raw p-value	Adjusted p-value
Folic acid	Not detected	Not detected
3-Indoxylsulfuric acid	Not detected	Not detected
Methyl sulfate	Not detected	Not detected
N-γ-Ethyl-L-glutamine	Not detected	Not detected
Norspermidine	Not detected	Not detected
4-Coumaric acid	Not detected	Not detected
N2-Phenylacetylglutamine	Not detected	Not detected
Pipecolic acid	Not detected	Not detected
Pyridoxal 5'-phosphoric acid	Not detected	Not detected
Pyridoxine	Not detected	Not detected
Riboflavin	Not detected	Not detected
Taurine	Not detected	Not detected
Trimethylamine N-oxide	Not detected	Not detected
3,4-Dihydroxyphenylacetaldehyde	Not detected	Not detected
3,4-Dihydroxyphenylacetic acid	Not detected	Not detected
Hydroxytyrosol	Not detected	Not detected
Methyldopa	Not detected	Not detected
4-Aminophenylalanine	Not detected	Not detected
4-Aminophenylpyruvic acid	Not detected	Not detected
Caffeic acid	Not detected	Not detected
Catechol	Not detected	Not detected
Ferulic acid	Not detected	Not detected
Protocatechualdehyde	Not detected	Not detected
Protocatechuic acid	Not detected	Not detected
Salicylic acid	Not detected	Not detected
Sinapic acid	Not detected	Not detected
Vanillic acid	Not detected	Not detected
Cysteine-glutathione disulphide	Not detected	Not detected
N1-Acetylspermine	Not detected	Not detected
N-Carbamoyl-L-aspartic acid	Not detected	Not detected
Saccharic acid	Not detected	Not detected
Serine O-sulfate	Not detected	Not detected
Sorbitol 6-phosphate	Not detected	Not detected

Table S11 (continued)

Metabolites	Raw p-value	Adjusted p-value
Thiamine	Not detected	Not detected
2,3-Pyridinedicarboxylic acid	Not detected	Not detected
Chenodeoxycholic acid	Not detected	Not detected
Deoxycholic acid	Not detected	Not detected
Dodecanoic acid	Not detected	Not detected
Hyodeoxycholic acid	Not detected	Not detected
Lithocholic acid	Not detected	Not detected
Maleic acid	Not detected	Not detected
Prostaglandin E2	Not detected	Not detected
Ursodeoxycholic acid	Not detected	Not detected
5-Diphosphomevalonic acid	Not detected	Not detected

Table S12. MRM parameters for targeted metabolites. Ionization mode, adduct ion, and fragmentation energy used for MRM quantification are listed for each metabolites.

Metabolites	Ionization mode	Adduct ion	Fragmentation energy (eV)
Aconitic acid	Negative	$[M-H]^-$	14
Citric acid	Negative	$[M-H]^-$	13
Fumaric acid	Negative	$[M-H]^-$	11
Isocitric acid	Negative	$[M-H]^-$	15
Malic acid	Negative	$[M-H]^-$	17
Succinic acid	Negative	$[M-H]^-$	13
3-Dehydroquinic acid	Negative	$[M-H]^-$	12
3-Dehydroshikimic acid	Negative	$[M-H]^-$	17
Chorismic acid	Negative	$[M-H]^-$	16
Shikimic acid	Negative	$[M-H]^-$	20
Phenylpyruvic acid	Negative	$[M-H]^-$	11
3-Phosphoshikimic acid	Negative	$[M-H]^-$	17

Table S12 (continued)

Metabolites	Ionization mode	Adduct ion	Fragmentation energy (eV)
Phenyllactic acid	Negative	$[M-H]^-$	15
Anthranilic acid	Positive	$[M+H]^+$	-15
Mevalonic acid	Negative	$[M-H]^-$	12
5-Phosphomevalonic acid	Negative	$[M-H]^-$	20
5-Diphosphomevalonic acid	Negative	$[M-H]^-$	39
1-Deoxy-D-xylulose 5-phosphoric acid	Negative	$[M-H]^-$	13
2-C-Methyl-D-erythritol 4-phosphoric acid	Negative	$[M-H]^-$	20
Biotin	Positive	$[M+H]^+$	-11
Flavin adenine dinucleotide	Positive	$[M+H]^+$	-47
Flavin mononucleotide	Negative	$[M-H]^-$	28
Nicotinamide adenine dinucleotide	Negative	$[M-H]^-$	17
Cholic acid	Negative	$[M-H]^-$	34
Taurocholic acid	Negative	$[M-H]^-$	55
Pyridoxine	Positive	$[M+H]^+$	-15
Riboflavin	Positive	$[M+H]^+$	-25
Nicotinic acid	Positive	$[M+H]^+$	-22
Pantothenic acid	Positive	$[M+H]^+$	-15
Nicotinamide	Positive	$[M+H]^+$	-23
Cystathionine	Positive	$[M+H]^+$	-27
Cysteine	Positive	$[M+H]^+$	-16
Homocysteine	Positive	$[M+H]^+$	-13
5-Glutamylcysteine	Positive	$[M+H]^+$	-26
Glutathione	Positive	$[M+H]^+$	-13
Oxidized glutathione	Negative	$[M-H]^-$	24
S-Adenosylhomocysteine	Positive	$[M+H]^+$	-21
S-Adenosylmethionine	Positive	$[M+H]^+$	-16
Folic acid	Negative	$[M-H]^-$	22
Pyridoxal 5'-phosphoric acid	Positive	$[M+H]^+$	-16
3,4-Dihydroxyphenylacetaldehyde	Negative	$[M-H]^-$	16
3,4-Dihydroxyphenylacetic acid	Negative	$[M-H]^-$	12
Hydroxytyrosol	Negative	$[M-H]^-$	17
Methyldopa	Positive	$[M+H]^+$	-15
Tyramine	Positive	$[M+H]^+$	-14
Hypoxanthine	Positive	$[M+H]^+$	-32
4-Aminobenzoic acid	Positive	$[M+H]^+$	-22
4-Aminophenylalanine	Positive	$[M+H]^+$	-17
4-Aminophenylpyruvic acid	Positive	$[M+H]^+$	-26
4-Hydroxybenzoic acid	Negative	$[M-H]^-$	15

Table S12 (continued)

Metabolites	Ionization mode	Adduct ion	Fragmentation energy (eV)
Caffeic acid	Negative	$[M-H]^-$	17
Catechol	Negative	$[M-H]^-$	21
Ferulic acid	Negative	$[M-H]^-$	18
4-Coumaric acid	Negative	$[M-H]^-$	16
Protocatechualdehyde	Positive	$[M+H]^+$	-13
Protocatechuic acid	Negative	$[M-H]^-$	15
Salicylic acid	Negative	$[M-H]^-$	17
Sinapic acid	Positive	$[M+H]^+$	-10
Vanillic acid	Positive	$[M+H]^+$	-15
Vanillin	Positive	$[M+H]^+$	-23
Indole	Positive	$[M+H]^+$	-23
5-Aminovaleric acid	Positive	$[M+H]^+$	-14
2-Oxoisopentanoic acid	Negative	$[M-H]^-$	11
5-Aminolevulinic acid	Positive	$[M+H]^+$	-19
2-Hydroxyisocaproic acid	Negative	$[M-H]^-$	15
4-Acetylbutyric acid	Positive	$[M+H]^+$	-14
Acetylcarnitine	Positive	$[M+H]^+$	-22
4-Aminobutyric acid	Positive	$[M+H]^+$	-14
2-Aminobutyric acid	Positive	$[M+H]^+$	-12
Pipecolic acid	Positive	$[M+H]^+$	-15
2-Isopropylmalic acid	Negative	$[M-H]^-$	16
2,6-Diaminopimelic acid	Positive	$[M+H]^+$	-15
Creatine	Positive	$[M+H]^+$	-22
Orotic acid	Negative	$[M-H]^-$	13
Adenine	Positive	$[M+H]^+$	-26
Cytosine	Positive	$[M+H]^+$	-23
Guanine	Negative	$[M-H]^-$	19
Thymine	Positive	$[M+H]^+$	-29
Uracil	Positive	$[M+H]^+$	-17
Xanthine	Negative	$[M-H]^-$	20
Adenosine	Positive	$[M+H]^+$	-18
Cytidine	Positive	$[M+H]^+$	-13
Guanosine	Positive	$[M+H]^+$	-12
Inosine	Positive	$[M+H]^+$	-10
Thymidine	Positive	$[M+H]^+$	-12
Uridine	Positive	$[M+H]^+$	-10
Adenosine 3',5'-cyclic phosphoric acid	Positive	$[M+H]^+$	-26
Adenosine 5'-monophosphoric acid	Positive	$[M+H]^+$	-20
Cytidine 3',5'-cyclic monophosphoric acid	Positive	$[M+H]^+$	-22
Cytidine-5'-monophosphoric acid	Positive	$[M+H]^+$	-14

Table S12 (continued)

Metabolites	Ionization mode	Adduct ion	Fragmentation energy (eV)
Guanosine 3',5'-cyclic monophosphoric acid	Positive	$[M+H]^+$	-22
Guanosine 5'-monophosphoric acid	Positive	$[M+H]^+$	-17
Thymidine 5'-monophosphoric acid	Positive	$[M+H]^+$	-22
2'-Deoxyinosine	Positive	$[M+H]^+$	-16
5'-Methylthioadenosine	Positive	$[M+H]^+$	-20
5'-Deoxyadenosine	Positive	$[M+H]^+$	-16
Adenylsuccinic acid	Positive	$[M+H]^+$	-21
4-Hydroxyproline	Positive	$[M+H]^+$	-15
Alanine	Positive	$[M+H]^+$	-12
Arginine	Positive	$[M+H]^+$	-23
Asparagine	Positive	$[M+H]^+$	-12
Aspartic acid	Positive	$[M+H]^+$	-15
Asymmetric dimethylarginine	Positive	$[M+H]^+$	-25
Citrulline	Positive	$[M+H]^+$	-25
Cystine	Positive	$[M+H]^+$	-14
Dimethylglycine	Positive	$[M+H]^+$	-16
Glutamic acid	Positive	$[M+H]^+$	-17
Glutamine	Positive	$[M+H]^+$	-18
Glycine	Positive	$[M+H]^+$	-11
Histidine	Positive	$[M+H]^+$	-15
Homocystine	Positive	$[M+H]^+$	-11
Isoleucine	Positive	$[M+H]^+$	-12
Leucine	Positive	$[M+H]^+$	-12
Lysine	Positive	$[M+H]^+$	-18
Methionine	Positive	$[M+H]^+$	-18
Methionine sulfoxide	Positive	$[M+H]^+$	-14
Ornithine	Positive	$[M+H]^+$	-18
Phenylalanine	Positive	$[M+H]^+$	-15
Proline	Positive	$[M+H]^+$	-18
Serine	Positive	$[M+H]^+$	-12
Symmetric dimethylarginine	Positive	$[M+H]^+$	-27
Threonine	Positive	$[M+H]^+$	-13
Tryptophan	Positive	$[M+H]^+$	-12
Tyrosine	Positive	$[M+H]^+$	-15
Valine	Positive	$[M+H]^+$	-13
AICAR	Negative	$[M-H]^-$	31
Histidinol	Positive	$[M+H]^+$	-23
Ergothioneine	Positive	$[M+H]^+$	-18
Argininosuccinic acid	Positive	$[M+H]^+$	-35
Ophthalmic acid	Positive	$[M+H]^+$	-23

Table S12 (continued)

Metabolites	Ionization mode	Adduct ion	Fragmentation energy (eV)
Urocanic acid	Negative	$[M-H]^-$	16
Carnitine	Positive	$[M+H]^+$	-18
Carnosine	Positive	$[M+H]^+$	-24
Trimethylamine	Positive	$[M+H]^+$	-23
Cadaverine	Positive	$[M+H]^+$	-13
Norspermidine	Positive	$[M+H]^+$	-19
Trimethylamine N-oxide	Positive	$[M+H]^+$	-20
Putrescine	Positive	$[M+H]^+$	-13
N1,N8-Diacetylspermidine	Positive	$[M+H]^+$	-20
Cysteamine	Positive	$[M+H]^+$	-13
Dopamine	Positive	$[M+H]^+$	-27
Histamine	Positive	$[M+H]^+$	-17
Serotonin	Positive	$[M+H]^+$	-13
N-Acetyl-L-valine	Positive	$[M+H]^+$	-20
N2-Phenylacetylglutamine	Positive	$[M+H]^+$	-16
γ-Butyrobetaine	Positive	$[M+H]^+$	-16
Taurine	Negative	$[M-H]^-$	22
N-ε-Acetyl-L-lysine	Positive	$[M+H]^+$	-25
N-γ-Ethyl-L-glutamine	Positive	$[M+H]^+$	-25
L-Cysteine S-sulfate	Positive	$[M+H]^+$	-14
Cycloleucine	Positive	$[M+H]^+$	-15
3-Indoxylsulfuric acid	Negative	$[M-H]^-$	22
Indole-3-acetic acid	Positive	$[M+H]^+$	-17
Methyl sulfate	Negative	$[M-H]^-$	21
Vanillylmandelic acid	Negative	$[M-H]^-$	22
1-Methyl-2-pyrrolidinone	Positive	$[M+H]^+$	-25
6-Methylaminopurine	Positive	$[M+H]^+$	-30
Proline betaine	Positive	$[M+H]^+$	-27
Uric acid	Negative	$[M-H]^-$	19
Acetylcholine	Positive	$[M+H]^+$	-16
Allantoin	Negative	$[M-H]^-$	15
Choline	Positive	$[M+H]^+$	-22
Citicoline	Positive	$[M+H]^+$	-43
Creatinine	Positive	$[M+H]^+$	-19
Dopa	Positive	$[M+H]^+$	-14
Adrenaline	Positive	$[M+H]^+$	-12
Kynurenine	Positive	$[M+H]^+$	-11
Noradrenaline	Positive	$[M+H]^+$	-10
Thiamine	Positive	$[M+H]^+$	-19
Saccharic acid	Negative	$[M-H]^-$	12

Table S12 (continued)

Metabolites	Ionization mode	Adduct ion	Fragmentation energy (eV)
4-Oxopentanoic acid	Positive	$[M+H]^+$	-9
Spermidine	Positive	$[M+H]^+$	-16
Spermine	Positive	$[M+H]^+$	-18
2-Phenylethylamine	Positive	$[M+H]^+$	-14
N1-Acetylspermine	Positive	$[M+H]^+$	-26
N1-Acetylspermidine	Positive	$[M+H]^+$	-22
Tryptamine	Positive	$[M+H]^+$	-14
α-Methylbenzylamine	Positive	$[M+H]^+$	-13
N1,N12-Diacetylspermine	Positive	$[M+H]^+$	-16
N-Carbamoyl-L-aspartic acid	Positive	$[M+H]^+$	-15
Serine O-sulfate	Negative	$[M-H]^-$	24
N-α-Benzoylarginine ethylester	Positive	$[M+H]^+$	-30
Cysteine-glutathione disulphide	Positive	$[M+H]^+$	-17
Phe-Phe	Positive	$[M+H]^+$	-20
Sorbitol 6-phosphate	Positive	$[M+H]^+$	-12
Picolinic acid butyl ester	Positive	$[M+H]^+$	-27
4-Hydroxymethylimidazole	Positive	$[M+H]^+$	-11
Pyruvic acid	Negative	$[M-H]^-$	22
2-Oxoglutaric acid	Positive	$[M+H]^+$	-30
Deoxycholic acid	Negative	$[M-H]^-$	39
Hyodeoxycholic acid	Negative	$[M-H]^-$	46
Lithocholic acid	Negative	$[M-H]^-$	45
Ursodeoxycholic acid	Negative	$[M-H]^-$	46
Chenodeoxycholic acid	Negative	$[M-H]^-$	47
3-(4-Hydroxyphenyl)propionic acid	Positive	$[M+H]^+$	-21
4-Hydroxyphenylacetic acid	Positive	$[M+H]^+$	-22
2,3-Pyridinedicarboxylic acid	Positive	$[M+H]^+$	-15
Benzoic acid	Positive	$[M+H]^+$	-15
3-Hydroxybenzoic acid	Positive	$[M+H]^+$	-16
3-Phenylpropionic acid	Positive	$[M+H]^+$	-17
Malonic acid	Negative	$[M-H]^-$	17
Maleic acid	Positive	$[M+H]^+$	-13
Crotonic acid	Positive	$[M+H]^+$	-14
Dodecanoic acid	Negative	$[M-H]^-$	25
2-Hydroxypentanoic acid	Negative	$[M-H]^-$	16
2-Oxoisocaproic acid	Negative	$[M-H]^-$	21
Butyric acid	Positive	$[M+H]^+$	-13
Propionic acid	Positive	$[M+H]^+$	-16
Isobutyric acid	Positive	$[M+H]^+$	-13
Valeric acid	Positive	$[M+H]^+$	-11

Table S12 (continued)

Metabolites	Ionization mode	Adduct ion	Fragmentation energy (eV)
Isovaleric acid	Positive	$[M+H]^+$	-14
2-Oxobutyric acid	Positive	$[M+H]^+$	-13
3-Hydroxybutyric acid	Positive	$[M+H]^+$	-9
2-Hydroxyglutaric acid	Positive	$[M+H]^+$	-14
Prostaglandin E2	Positive	$[M+H]^+$	-13

Table S13. Processed metabolite concentration values (intracellular and extracellular) for the KUHIMM experiments reported in this study.

Intracellular results (nmol/ dry-cell weight)

Sample name	1-Deoxy-D-xylulose 5-phosphoric acid	1-Methyl-2-pyrrolidinone	2,3-Pyridinedicarboxylic acid	2,6-Diaminopimelic acid	2-Aminobutyric acid	2-C-Methyl-D-erythritol 4-phosphoric acid	2'-Deoxyinosine	2-Hydroxyglutaric acid
CUL-24 h-1	N/A	0.0022622980	0.0018860940	0.0162771440	18.6221153800	0.0122409930	N/A	0.2972019230
CUL-24 h-2	N/A	0.0015845070	0.0030040970	0.0008794250	9.3931054370	0.0105077550	N/A	0.2701183690
CUL-24 h-3	N/A	0.0030321920	0.0024591120	0.0010574170	11.9048624700	0.0169232180	N/A	0.2242366540
INU-24 h-1	N/A	0.0022622980	0.0018860940	0.0162771440	18.6221153800	0.0122409930	N/A	0.2972019230
INU-24 h-2	N/A	0.0015845070	0.0030040970	0.0008794250	9.3931054370	0.0105077550	N/A	0.2701183690
INU-24 h-3	N/A	0.0030321920	0.0024591120	0.0010574170	11.9048624700	0.0169232180	N/A	0.2242366540
CUL-48 h-1	N/A	0.0040139210	0.0023759190	0.0012467290	30.1517566900	0.0104826430	N/A	0.2714465130
CUL-48 h-2	N/A	0.0049653930	0.0027391560	0.0015072160	11.2950111600	0.0153413800	N/A	0.4361016360
CUL-48 h-3	N/A	0.0048736150	0.0027660190	0.0016022430	29.6325520800	0.0183212980	N/A	0.3392465280
INU-48 h-1	N/A	0.0032255590	0.0008706030	0.0016708750	53.0763132500	0.0078104360	N/A	0.0484190820
INU-48 h-2	N/A	0.0043414220	0.0022849330	0.0015558910	35.4924922900	0.0077926960	N/A	0.1429331790
INU-48 h-3	N/A	0.0034413100	0.0014456320	0.0011285370	33.3985888000	0.0091709080	N/A	0.0785128420
CUL-72 h-1	N/A	0.0055600060	0.0025430090	0.0004756010	3.8947025170	0.0070616190	N/A	0.3233765730
CUL-72 h-2	N/A	0.0061839450	0.0015930190	0.0024802840	7.9559332510	0.0069559920	N/A	0.1514472190
CUL-72 h-3	N/A	0.0067390930	0.0028115460	0.0011650100	3.1761794280	0.0048876000	N/A	0.3222156160
INU-72 h-1	N/A	0.0051258550	0.0009098650	0.1573783520	16.6911921100	0.0049429480	N/A	0.0704749040
INU-72 h-2	N/A	0.0051718220	0.0016740450	0.0100844200	20.3105534700	0.0061582100	N/A	0.0955136060
INU-72 h-3	N/A	0.0047496470	0.0018164120	0.0024242180	65.0851907900	0.0027821490	N/A	0.0799699080

Sample name	2-Hydroxyisocaproic acid	2-Hydroxypentanoic acid	2-Isopropylmalic acid	2-Oxoglutaric acid	2-Oxobutyric acid	2-Oxoisocaproic acid	2-Oxoisopentanoic acid	2-Phenylethylamine	3-(4-Hydroxyphenyl)propionic acid	3,4-Dihydroxyphenylacetaldehyde
CUL-24 h-1	2.4632886170	1240.1000310000	0.0032792430	0.0357603640	0.0466782030	1373.0466810000	N/A	0.0002141720	0.0548227900	N/A
CUL-24 h-2	1.2203727350	1027.6449750000	0.0043159060	0.0327789500	0.0414957990	973.5115321000	N/A	0.0001567050	0.0595430810	N/A
CUL-24 h-3	1.8231225590	1299.4213870000	0.0057315470	0.0356343590	0.0343093100	1109.6598310000	N/A	0.0001714000	0.0459910970	N/A
INU-24 h-1	2.4632886170	1240.1000310000	0.0032792430	0.0357603640	0.0466782030	1373.0466810000	N/A	0.0002141720	0.0548227900	N/A
INU-24 h-2	1.2203727350	1027.6449750000	0.0043159060	0.0327789500	0.0414957990	973.5115321000	N/A	0.0001567050	0.0595430810	N/A
INU-24 h-3	1.8231225590	1299.4213870000	0.0057315470	0.0356343590	0.0343093100	1109.6598310000	N/A	0.0001714000	0.0459910970	N/A
CUL-48 h-1	2.3601337180	650.8485842000	0.0048580960	0.0600320600	0.0324980070	721.1999213000	N/A	0.0004350790	0.1661377820	N/A
CUL-48 h-2	0.6306017600	1975.7969760000	0.0067739590	0.0504917950	0.0341607210	524.0417700000	N/A	0.0008157380	0.2151783590	N/A
CUL-48 h-3	2.0083965770	2788.1125990000	0.0104295940	0.0559912020	0.0380929940	1170.4319200000	N/A	0.0007497160	0.1319857390	N/A
INU-48 h-1	6.1159466120	2953.1861060000	0.0025682050	0.0693704970	0.0143930000	213.3386578000	N/A	0.0002107200	0.3081167450	N/A
INU-48 h-2	5.1247473580	3639.1567590000	0.0030179520	0.0852607880	0.0299555590	194.5237781000	N/A	0.0002192280	0.4566835090	N/A
INU-48 h-3	1.3894420320	2622.0701260000	0.0034455210	0.0787533110	0.0174552320	261.5194312000	N/A	0.0002462620	0.3437465260	N/A
CUL-72 h-1	N/A	3096.7162470000	0.0047631350	0.0794246280	0.0436248280	1769.7239700000	N/A	0.0068403570	0.1108992560	N/A
CUL-72 h-2	N/A	3302.2156980000	0.0031493380	0.0818739960	0.0318571230	1317.6282450000	N/A	0.0013643590	0.0663036000	N/A
CUL-72 h-3	N/A	2918.2682390000	0.0041825540	0.0819503250	0.0535877960	1755.0947410000	N/A	0.0036319400	0.1271607210	N/A
INU-72 h-1	7.9831968630	4130.4027310000	0.0020244620	0.0835729390	0.0220534950	824.1543390000	N/A	0.0005702550	0.2053488030	N/A
INU-72 h-2	8.8824825800	4032.9972550000	0.0027359820	0.0946454680	0.0343441990	1186.4525710000	N/A	0.0004552640	0.2180339420	N/A
INU-72 h-3	8.7117105260	2785.2618420000	0.0028181490	0.1148678290	0.0366911320	985.7500000000	N/A	0.0002944870	0.1477697370	N/A

Table S13 (continued)

Sample name	3-Dehydroquinic acid	3-Dehydroshikimic acid	3-Hydroxybenzoic acid	3-Hydroxybutyric acid	3-Indoxylsulfuric acid	3-Phenylpropionic acid	3-Phosphoshikimic acid	4-Acetylbutyric acid	4-Aminobenzoic acid
CUL-24 h-1	0.0132867670	N/A	0.0098928580	0.3971315910	N/A	3.2148867870	0.0040838980	N/A	5.2901674940
CUL-24 h-2	0.0028687350	N/A	0.0094923150	0.4226054370	N/A	2.9158294890	0.0039294400	N/A	4.1642668860
CUL-24 h-3	0.0041531050	N/A	0.0084158120	0.3299474280	N/A	3.8226383460	0.0062581830	N/A	4.3369873050
INU-24 h-1	0.0132867670	N/A	0.0098928580	0.3971315910	N/A	3.2148867870	0.0040838980	N/A	5.2901674940
INU-24 h-2	0.0028687350	N/A	0.0094923150	0.4226054370	N/A	2.9158294890	0.0039294400	N/A	4.1642668860
INU-24 h-3	0.0041531050	N/A	0.0084158120	0.3299474280	N/A	3.8226383460	0.0062581830	N/A	4.3369873050
CUL-48 h-1	0.0025793770	N/A	0.0108309960	0.6030953070	N/A	27.6826953300	0.0031220410	N/A	8.5119756160
CUL-48 h-2	0.0027334910	N/A	0.0121562000	0.5669103870	N/A	35.2404933100	0.0045965190	N/A	6.9310299950
CUL-48 h-3	0.0027124700	N/A	0.0116285830	0.5840018600	N/A	22.5426711300	0.0044324410	N/A	8.4993365580
INU-48 h-1	0.0024473920	N/A	0.0056244530	0.3087513940	N/A	29.3232740100	0.0033481230	N/A	11.7452991000
INU-48 h-2	0.0030336670	N/A	0.0077834950	0.5843710920	N/A	35.8054271200	0.0026738080	N/A	9.5650099080
INU-48 h-3	0.0035967290	N/A	0.0055400620	0.3929781810	N/A	24.4763894900	0.0024017290	N/A	8.5665002170
CUL-72 h-1	0.0014005910	N/A	0.0189411040	0.7824559500	N/A	8.6751544620	0.0005867190	0.0076832310	7.5966604690
CUL-72 h-2	0.0040577130	N/A	0.0110996170	0.5654466930	N/A	8.4911186650	0.0019066300	0.0093180080	10.9614415900
CUL-72 h-3	0.0022346360	N/A	0.0195901810	0.9359277520	N/A	9.3172998840	0.0014711510	0.0112115320	8.2212957590
INU-72 h-1	0.0451089760	N/A	0.0072352340	0.5079451560	N/A	28.0326167600	0.0018448630	0.0033473290	8.9026630220
INU-72 h-2	0.0089435770	N/A	0.0138854390	0.8165805800	N/A	35.0433118700	0.0024913790	0.0039467460	8.9144650020
INU-72 h-3	0.0026486110	N/A	0.0130757620	0.9115461840	N/A	20.0939078900	0.0013227710	0.0046001010	13.5441776300

Sample name	4-Aminobutyric acid	4-Aminophenylalanine	4-Aminophenylpyruvic acid	4-Coumaric acid	4-Hydroxybenzoic acid	4-Hydroxymethylimidazole	4-Hydroxyphenylacetic acid	4-Hydroxyproline	4-Oxopentanoic acid
CUL-24 h-1	32.7754303700	N/A	N/A	N/A	0.0099865850	0.0031510850	0.0663845690	0.3603830650	0.0339818550
CUL-24 h-2	25.8357619400	N/A	N/A	N/A	0.0078248310	0.0030198040	0.0604414500	0.0256969400	0.0255547650
CUL-24 h-3	26.6596842400	N/A	N/A	N/A	0.0139280920	0.0044812420	0.0408483240	0.0263602500	0.0241224490
INU-24 h-1	32.7754303700	N/A	N/A	N/A	0.0099865850	0.0031510850	0.0663845690	0.3603830650	0.0339818550
INU-24 h-2	25.8357619400	N/A	N/A	N/A	0.0078248310	0.0030198040	0.0604414500	0.0256969400	0.0255547650
INU-24 h-3	26.6596842400	N/A	N/A	N/A	0.0139280920	0.0044812420	0.0408483240	0.0263602500	0.0241224490
CUL-48 h-1	49.2578919800	N/A	N/A	N/A	0.0230984010	0.0057104720	0.0440214340	0.0124097010	0.0671518750
CUL-48 h-2	43.8283775400	N/A	N/A	N/A	0.0338592150	0.0043252110	0.0356126800	0.0340713560	0.0675457360
CUL-48 h-3	49.8974082300	N/A	N/A	N/A	0.0458364090	0.0052776050	0.0573952380	0.0272493680	0.0666369050
INU-48 h-1	74.8559712700	N/A	N/A	N/A	0.0224711250	0.0057183430	0.0242167990	0.0226999890	0.0463109780
INU-48 h-2	52.6260017600	N/A	N/A	N/A	0.0225013980	0.0051523120	0.0562799210	0.0162046350	0.0511790950
INU-48 h-3	56.6350955300	N/A	N/A	N/A	0.0218977960	0.0061030230	0.0304434650	0.0261283110	0.0606270080
CUL-72 h-1	42.3917262600	N/A	N/A	N/A	0.0504059710	0.0042550820	0.0275181780	0.0069008090	0.0699775530
CUL-72 h-2	59.8809255300	N/A	N/A	N/A	0.0443108390	0.0064176890	0.0227190820	0.0063005160	0.1024208900
CUL-72 h-3	43.2236965100	N/A	N/A	N/A	0.0447488880	0.0052672700	0.0177681100	0.0123718900	0.1440045760
INU-72 h-1	47.1087287300	N/A	N/A	N/A	0.0263216650	0.0066464970	0.0123056240	1.0376162680	0.0706786190
INU-72 h-2	44.5871714000	N/A	N/A	N/A	0.0310501870	0.0062932530	0.0289353620	0.0664258470	0.0689080980
INU-72 h-3	73.1814473700	N/A	N/A	N/A	0.0283800990	0.0070561510	0.0176547890	0.0174945660	0.0744150660

Table S13 (continued)

Sample name	5-Aminolevulinic acid	5-Aminovaleric acid	5'-Deoxyadenosine	5-Diphosphomevalonic acid	5-Glutamylcysteine	5'-Methylthioadenosine	5-Phosphomevalonic acid	6-Methylaminopurine	Acetylcarnitine
CUL-24 h-1	N/A	15.9167920300	0.0019637350	N/A	N/A	0.1255523810	0.0004449350	0.0000194000	0.0000573000
CUL-24 h-2	N/A	11.9033525500	0.0017754600	N/A	N/A	0.1097128500	0.0002219500	0.0000148000	0.0000690000
CUL-24 h-3	N/A	10.7627319300	0.0025581880	N/A	N/A	0.1110298670	0.0005190650	0.0000267000	0.0000581000
INU-24 h-1	N/A	15.9167920300	0.0019637350	N/A	N/A	0.1255523810	0.0004449350	0.0000194000	0.0000573000
INU-24 h-2	N/A	11.9033525500	0.0017754600	N/A	N/A	0.1097128500	0.0002219500	0.0000148000	0.0000690000
INU-24 h-3	N/A	10.7627319300	0.0025581880	N/A	N/A	0.1110298670	0.0005190650	0.0000267000	0.0000581000
CUL-48 h-1	N/A	21.6495804900	0.0018848030	N/A	N/A	0.1153680520	0.0004200650	0.0000053900	0.0000688000
CUL-48 h-2	N/A	21.6330565200	0.0024204900	N/A	N/A	0.0922702030	0.0009809100	0.0000746000	0.0001081520
CUL-48 h-3	N/A	21.6533916200	0.0019575710	N/A	N/A	0.1196779510	0.0008619520	0.0000114000	0.0000785000
INU-48 h-1	N/A	31.1698756400	0.0015136230	N/A	N/A	0.2198872750	0.0004012130	0.0000063800	0.0001072870
INU-48 h-2	N/A	24.1003467600	0.0022132210	N/A	N/A	0.2061908850	0.0005878770	0.0000120000	0.0000820000
INU-48 h-3	N/A	25.8917010400	0.0017598570	N/A	N/A	0.1798314160	0.0007210950	0.0000170000	0.0000743000
CUL-72 h-1	N/A	20.3526887900	0.0022238630	N/A	N/A	0.0534421980	0.0008293050	0.0000239000	0.0000930000
CUL-72 h-2	N/A	30.3689894900	0.0022973790	N/A	N/A	0.1111885040	0.0009089380	0.0000726000	0.0001358330
CUL-72 h-3	N/A	18.7764243400	0.0017503340	N/A	N/A	0.0697195800	0.0007361040	0.0000529000	0.0001143190
INU-72 h-1	N/A	25.6376410100	0.0020093600	N/A	N/A	0.1773694460	0.0008175420	0.0000670000	0.0000939000
INU-72 h-2	N/A	20.6543100700	0.0024380640	N/A	N/A	0.1080070210	0.0006176500	0.0000307000	0.0001472800
INU-72 h-3	N/A	34.3385131600	0.0022518160	N/A	N/A	0.1013064470	0.0008209380	0.0000138000	0.0001141350

Sample name	Acetylcholine	Aconitic acid	Adenine	Adenosine	Adenosine 3',5'-cyclic phosphoric acid	Adenosine 5'-monophosphoric acid	Adenylsuccinic acid	Adrenaline	AICAR	Alanine	Allantoin	Anthranilic acid
CUL-24 h-1	N/A	N/A	0.1267670980	0.9497371280	0.0001661650	0.9843653070	0.0034145760	N/A	N/A	22.4172883100	N/A	0.0033693840
CUL-24 h-2	N/A	N/A	0.1372132210	0.7798883860	0.0000705000	0.6270243000	0.0041016800	N/A	N/A	16.2951976900	N/A	0.0024291800
CUL-24 h-3	N/A	N/A	0.1358016360	0.9519470210	0.0001181660	0.5172863770	0.0033171110	N/A	N/A	17.8337972000	N/A	0.0034220390
INU-24 h-1	N/A	N/A	0.1267670980	0.9497371280	0.0001661650	0.9843653070	0.0034145760	N/A	N/A	22.4172883100	N/A	0.0033693840
INU-24 h-2	N/A	N/A	0.1372132210	0.7798883860	0.0000705000	0.6270243000	0.0041016800	N/A	N/A	16.2951976900	N/A	0.0024291800
INU-24 h-3	N/A	N/A	0.1358016360	0.9519470210	0.0001181660	0.5172863770	0.0033171110	N/A	N/A	17.8337972000	N/A	0.0034220390
CUL-48 h-1	N/A	N/A	0.2274835470	0.4790168460	0.0003285770	0.9706213950	0.0070583770	N/A	N/A	4.4124895120	N/A	0.0184454900
CUL-48 h-2	N/A	N/A	0.2250095440	0.4952857590	0.0004435430	0.8414978930	0.0138945650	N/A	N/A	1.1210777140	N/A	0.0228131380
CUL-48 h-3	N/A	N/A	0.2557211060	0.5778638390	0.0004432960	0.8411960570	0.0061513750	N/A	N/A	4.2576103670	N/A	0.0243022820
INU-48 h-1	N/A	N/A	0.1257203040	1.1464885290	0.0002238920	1.0709718050	0.0023642390	N/A	N/A	13.2207761600	N/A	0.0051084630
INU-48 h-2	N/A	N/A	0.1918879900	0.5884648830	0.0002146310	0.5332628250	0.0024405210	N/A	N/A	10.2561261600	N/A	0.0038376430
INU-48 h-3	N/A	N/A	0.1499503370	0.7113411850	0.0001882260	0.4936778120	0.0016448110	N/A	N/A	9.4959672170	N/A	0.0032915410
CUL-72 h-1	N/A	N/A	0.1174862700	0.2073912330	0.0004113420	0.7226601830	0.0012119720	N/A	N/A	0.3005903890	N/A	0.0310254220
CUL-72 h-2	N/A	N/A	0.2110676760	0.4172288320	0.0003562140	1.5891679540	0.0027502440	N/A	N/A	0.2204157910	N/A	0.0250152660
CUL-72 h-3	N/A	N/A	0.2118194930	0.2553240200	0.0002827710	1.0891692450	0.0043354460	N/A	N/A	0.1256498940	N/A	0.0280392260
INU-72 h-1	N/A	N/A	0.2194988740	0.7159385510	0.0001897210	0.4693494580	0.0022243620	N/A	N/A	4.3358196860	N/A	0.0042715790
INU-72 h-2	N/A	N/A	0.2423696820	0.3877209800	0.0003237070	0.4508284950	0.0017793010	N/A	N/A	4.5069969380	N/A	0.0027227740
INU-72 h-3	N/A	N/A	0.1581122370	0.6869921050	0.0001063790	0.4027794740	0.0011702290	N/A	N/A	26.0725789500	N/A	0.0054660490

Table S13 (continued)

Sample name	Arginine	Argininosuccinic acid	Asparagine	Aspartic acid	Asymmetric dimethylarginine	Benzoic acid	Biotin	Butyric acid	Cadaverine	Caffeic acid	Carnitine
CUL-24 h-1	0.6660150430	0.0006945100	0.6964267990	5.6192424010	0.0396213170	0.2568643770	N/A	6.8817997830	3.7068815910	N/A	0.0039782030
CUL-24 h-2	0.5310724880	0.0006101180	0.5778809720	4.2881919280	0.0367126850	0.3119223230	N/A	9.8534019770	2.9983995060	N/A	0.0046189210
CUL-24 h-3	0.5545581050	0.0008185030	0.5121879070	3.6018505860	0.0362542770	0.2329313150	N/A	8.0647290040	3.1550508630	N/A	0.0046687170
INU-24 h-1	0.6660150430	0.0006945100	0.6964267990	5.6192424010	0.0396213170	0.2568643770	N/A	6.8817997830	3.7068815910	N/A	0.0039782030
INU-24 h-2	0.5310724880	0.0006101180	0.5778809720	4.2881919280	0.0367126850	0.3119223230	N/A	9.8534019770	2.9983995060	N/A	0.0046189210
INU-24 h-3	0.5545581050	0.0008185030	0.5121879070	3.6018505860	0.0362542770	0.2329313150	N/A	8.0647290040	3.1550508630	N/A	0.0046687170
CUL-48 h-1	0.7980715780	N/A	0.3877355790	2.1776763240	0.0282586520	0.3694800730	0.0032518000	15.0987808100	7.8559910850	N/A	0.0016812860
CUL-48 h-2	0.8524082800	N/A	0.6557356220	2.1823425880	0.0269112480	0.3400798220	0.0043135790	12.3156990600	7.0852689640	N/A	0.0019721920
CUL-48 h-3	0.6466313240	N/A	0.6506901040	2.1863244050	0.0200763950	0.3718828130	0.0032344930	13.4753224200	7.7238901290	N/A	0.0032563550
INU-48 h-1	1.8439708400	0.0005230540	0.9628859350	2.7412349910	0.0309206530	0.1788668520	0.0023404030	10.9458619200	10.9947202000	N/A	0.0039024660
INU-48 h-2	1.5232342580	0.0007319580	0.4182825850	1.7398904670	0.0372605240	0.3140841040	0.0026000030	14.7702003500	9.4496477320	N/A	0.0031573440
INU-48 h-3	1.3403430310	0.0008753290	0.7272964610	1.8150542770	0.0303284470	0.2239461570	0.0026280260	11.9402518500	8.8941489360	N/A	0.0061124240
CUL-72 h-1	0.3376306490	N/A	0.3624830520	1.1277302630	0.0129726190	0.5069991420	0.0026728630	22.8009153300	11.9212743100	N/A	0.0007397890
CUL-72 h-2	0.7567867740	N/A	0.2462294500	2.1930554700	0.0105516220	0.3575693760	0.0030468650	20.2102132300	13.8782911000	N/A	0.0029810010
CUL-72 h-3	0.6014149100	N/A	0.2621639760	1.3690778870	0.0114082320	0.6021505220	0.0040313760	29.4616686000	11.1670211400	N/A	0.0020558960
INU-72 h-1	0.8326402630	N/A	0.7733450430	1.6987977440	0.0211104290	0.2710619930	0.0021937890	10.3099928300	11.0152199200	N/A	0.0056797490
INU-72 h-2	0.6502110170	N/A	0.6975071260	1.1772401550	0.0328859140	0.4177781880	0.0030559970	17.5127085100	8.2933052680	N/A	0.0072921840
INU-72 h-3	0.9814197370	N/A	0.5802250000	1.0582105260	0.0392570130	0.4656665790	0.0033698520	24.6666184200	17.4346447400	N/A	0.0024506050

Sample name	Carnosine	Catechol	Chenodeoxycholic acid	Cholic acid	Choline	Chorismic acid	Citicoline	Citric acid	Citrulline	Creatine	Creatinine	Crotonic acid	Cycloleucine
CUL-24 h-1	0.0016582550	N/A	N/A	N/A	0.2173280860	N/A	0.0039782030	0.0140924430	2.8755823510	0.0001657200	0.0158877750	0.0223416330	0.0921714100
CUL-24 h-2	0.0011835820	N/A	N/A	N/A	0.1433752880	N/A	0.0046189210	0.0092985170	2.0408393740	0.0001083660	0.0094695550	0.0263684600	0.0627651570
CUL-24 h-3	0.0019520740	N/A	N/A	N/A	0.1439219160	N/A	0.0046687170	0.0126070720	2.0759529620	0.0001122290	0.0121294720	0.0234645430	0.0611561690
INU-24 h-1	0.0016582550	N/A	N/A	N/A	0.2173280860	N/A	0.0039782030	0.0140924430	2.8755823510	0.0001657200	0.0158877750	0.0223416330	0.0921714100
INU-24 h-2	0.0011835820	N/A	N/A	N/A	0.1433752880	N/A	0.0046189210	0.0092985170	2.0408393740	0.0001083660	0.0094695550	0.0263684600	0.0627651570
INU-24 h-3	0.0019520740	N/A	N/A	N/A	0.1439219160	N/A	0.0046687170	0.0126070720	2.0759529620	0.0001122290	0.0121294720	0.0234645430	0.0611561690
CUL-48 h-1	0.0012587720	N/A	N/A	N/A	0.1396464340	N/A	0.0016812860	0.0174157970	0.8607583900	0.0000876000	0.0041615440	0.0363968410	0.1122127690
CUL-48 h-2	0.0012168180	N/A	N/A	N/A	0.1046698690	N/A	0.0019721920	0.0284379710	0.6140569530	0.0000884000	0.0039536770	0.0382506570	0.0891380760
CUL-48 h-3	0.0020734430	N/A	N/A	N/A	0.1273410220	N/A	0.0032563550	0.0326092880	0.5276610860	0.0001446420	0.0060429330	0.0415596480	0.1156733010
INU-48 h-1	0.0011617620	N/A	N/A	N/A	0.0731299310	N/A	0.0039024660	0.0057513780	1.1121928600	0.0001670390	0.0042881700	0.0280562930	0.1754261360
INU-48 h-2	0.0015167560	N/A	N/A	N/A	0.0714755610	N/A	0.0031573440	0.0048683760	1.3217404230	0.0002498110	0.0081869770	0.0312429110	0.1339438570
INU-48 h-3	0.0010209390	N/A	N/A	N/A	0.0708905230	N/A	0.0061124240	0.0063125430	0.8817515200	0.0003175770	0.0087130320	0.0262302210	0.1578147520
CUL-72 h-1	0.0010912890	N/A	N/A	N/A	0.0201554420	N/A	0.0007397890	0.0079208450	0.1514995710	0.0000973000	0.0005092740	0.0455581380	0.1570779460
CUL-72 h-2	0.0010591230	N/A	N/A	N/A	0.0587987480	N/A	0.0029810010	0.0174939890	0.3555571690	0.0000715000	0.0010599530	0.0438390760	0.2677548670
CUL-72 h-3	0.0010959200	N/A	N/A	N/A	0.0264083040	N/A	0.0020558960	0.0107824100	0.1845503830	0.0006025310	0.0009281150	0.0547895880	0.2165874740
INU-72 h-1	0.0010191540	N/A	N/A	N/A	0.0307351640	N/A	0.0056797490	0.0056164970	0.5141717790	0.0001950490	0.0034374840	0.0279134800	0.1642958390
INU-72 h-2	0.0000911000	N/A	N/A	N/A	0.0182837380	N/A	0.0072921840	0.0057905670	0.2651644320	0.0003797220	0.0041307490	0.0404449300	0.1463074060
INU-72 h-3	0.0013272280	N/A	N/A	N/A	0.0329545130	N/A	0.0024506050	0.0060345960	0.3928880260	0.0002378550	0.0049369730	0.0441817890	0.2540017760

Table S13 (continued)

Sample name	Cystathionine	Cysteamine	Cysteine	Cysteine-glutathione disulphide	Cystine	Cytidine	Cytidine 3',5'-cyclic monophosphoric acid	Cytidine-5'-monophosphoric acid	Cytosine
CUL-24 h-1	0.0013382330	N/A	4.1312771400	N/A	0.0156055060	0.0390591270	N/A	0.0005395740	0.0046137290
CUL-24 h-2	0.0001730440	N/A	2.7815185340	N/A	0.0122948350	0.0348073310	N/A	0.0004197880	0.0041643370
CUL-24 h-3	0.0005066110	N/A	1.5270629880	N/A	0.0098314090	0.0330178260	N/A	0.0003116770	0.0053823200
INU-24 h-1	0.0013382330	N/A	4.1312771400	N/A	0.0156055060	0.0390591270	N/A	0.0005395740	0.0046137290
INU-24 h-2	0.0001730440	N/A	2.7815185340	N/A	0.0122948350	0.0348073310	N/A	0.0004197880	0.0041643370
INU-24 h-3	0.0005066110	N/A	1.5270629880	N/A	0.0098314090	0.0330178260	N/A	0.0003116770	0.0053823200
CUL-48 h-1	0.0000432000	N/A	5.1899023340	N/A	0.0222247840	0.0498701490	N/A	0.0004506710	0.0080811620
CUL-48 h-2	0.0000343000	N/A	4.7189111300	N/A	0.0081404690	0.0615273740	N/A	0.0006946490	0.0095986860
CUL-48 h-3	0.0000393000	N/A	4.5994723460	N/A	0.0150354100	0.0377289990	N/A	0.0006737120	0.0078530820
INU-48 h-1	0.0000580000	N/A	6.9046258580	N/A	0.0506090270	0.0422099110	N/A	0.0004399860	0.0051968710
INU-48 h-2	0.0002326720	N/A	6.3801959490	N/A	0.0121723190	0.0519305040	N/A	0.0003153840	0.0059484420
INU-48 h-3	0.0003725460	N/A	4.1388585540	N/A	0.0171221120	0.0438982520	N/A	0.0002909320	0.0056210320
CUL-72 h-1	0.0000161000	N/A	5.2363579810	N/A	0.0284631580	0.0241680710	N/A	0.0004048210	0.0091430920
CUL-72 h-2	0.0000375000	N/A	7.4242297590	N/A	0.0310203260	0.0570982000	N/A	0.0010246580	0.0115426140
CUL-72 h-3	0.0000458000	N/A	6.7734016500	N/A	0.0129061770	0.0483001660	N/A	0.0009326280	0.0095468630
INU-72 h-1	0.0015875900	N/A	5.9136385560	N/A	0.0359945520	0.0399264920	N/A	0.0003376120	0.0075669340
INU-72 h-2	0.0004708550	N/A	4.9678961680	N/A	0.0209117070	0.0537663710	N/A	0.0006881360	0.0088075840
INU-72 h-3	0.0000693000	N/A	7.5966578950	N/A	0.0309995460	0.0421534610	N/A	0.0004038000	0.0068406840

Sample name	Deoxycholic acid	Dimethylglycine	Dodecanoic acid	Dopa	Dopamine	Ergothioneine	Ferulic acid	Flavin adenine dinucleotide	Flavin mononucleotide	Folic acid	Fumaric acid	Glutamic acid	Glutamine
CUL-24 h-1	0.0102171680	N/A	N/A	0.0003369520	0.0063015080	0.0072786450	N/A	0.0339394970	0.0357966230	N/A	0.0016235290	19.8176682700	1.2136174010
CUL-24 h-2	0.0126612190	N/A	N/A	0.0001696430	0.0037752480	0.0077142500	N/A	0.0272520550	0.0286893620	N/A	0.0012081400	13.5665774300	1.5959176280
CUL-24 h-3	0.0093883540	N/A	N/A	0.0006142550	0.0014781100	0.0118333170	N/A	0.0370272140	0.0256747600	N/A	0.0012150910	12.8926513700	1.0164290360
INU-24 h-1	0.0102171680	N/A	N/A	0.0003369520	0.0063015080	0.0072786450	N/A	0.0339394970	0.0357966230	N/A	0.0016235290	19.8176682700	1.2136174010
INU-24 h-2	0.0126612190	N/A	N/A	0.0001696430	0.0037752480	0.0077142500	N/A	0.0272520550	0.0286893620	N/A	0.0012081400	13.5665774300	1.5959176280
INU-24 h-3	0.0093883540	N/A	N/A	0.0006142550	0.0014781100	0.0118333170	N/A	0.0370272140	0.0256747600	N/A	0.0012150910	12.8926513700	1.0164290360
CUL-48 h-1	0.0209307810	N/A	N/A	0.0003693630	0.0050840630	0.0028141070	N/A	0.0816952670	0.1253057160	N/A	0.0008471160	6.8361497120	0.5823139750
CUL-48 h-2	0.0193645140	N/A	N/A	0.0004950320	0.0018011250	0.0041234860	N/A	0.1190025410	0.1525708970	N/A	0.0007954130	5.3442854490	0.4469721120
CUL-48 h-3	0.0207182290	N/A	N/A	0.0005882660	0.0097760170	0.0049075890	N/A	0.0968467880	0.1375553080	N/A	0.0010386570	5.4943483380	0.5368293650
INU-48 h-1	0.0137616420	N/A	N/A	0.0005258150	0.0034558940	0.0067925650	N/A	0.0660217620	0.0890270150	N/A	0.0010017980	6.9591177100	2.1681710980
INU-48 h-2	0.0191127480	N/A	N/A	0.0010918090	0.0052273820	0.0060686540	N/A	0.0659479300	0.0922673930	N/A	0.0010501820	4.4676420080	0.6779551960
INU-48 h-3	0.0140811120	N/A	N/A	0.0010431160	0.0044807920	0.0071480130	N/A	0.0665375600	0.0640905340	N/A	0.0012146300	4.4623860180	0.6384883850
CUL-72 h-1	0.0422734550	N/A	N/A	0.0005854120	0.0022843660	0.0010275480	N/A	0.1169471540	0.1674838390	N/A	0.0003993790	0.9828046340	0.2419844110
CUL-72 h-2	0.0363382260	N/A	N/A	0.0004415070	0.0034617820	0.0026388780	N/A	0.1360245670	0.1420644310	N/A	0.0013507180	3.5412021010	0.5584393540
CUL-72 h-3	0.0775754380	N/A	N/A	0.0007936960	0.0042671700	0.0021189610	N/A	0.1555855250	0.1703210400	N/A	0.0010596550	2.1064127350	0.2861757220
INU-72 h-1	0.0228176080	N/A	N/A	0.0007857990	0.0028126330	0.0018373820	N/A	0.0908099770	0.1039928010	N/A	0.0014417060	4.3853187460	0.6495874980
INU-72 h-2	0.0357131810	N/A	N/A	0.0009220280	0.0083481510	0.0024536540	N/A	0.1282984060	0.1641132420	N/A	0.0014827310	2.8722986170	0.4000182780
INU-72 h-3	0.0401868680	N/A	N/A	0.0011975750	0.0293138360	0.0018494610	N/A	0.0630463360	0.1044033550	N/A	0.0008156710	2.4599065790	0.5412115790

Table S13 (continued)

Sample name	Glutathione	Glycine	Guanine	Guanosine	Guanosine 3',5'-cyclic monophosphoric acid	Guanosine 5'-monophosphoric acid	Histamine	Histidine	Histidinol
CUL-24 h-1	0.1232657030	5.2265353600	0.0295602630	0.0076120000	0.0001144900	0.0035738670	0.0152513690	0.2333029620	0.0006141040
CUL-24 h-2	0.0856177920	3.2255576610	0.0287400250	0.0100861330	0.0000230000	0.0038536560	0.0129936660	0.2261196870	0.0006714810
CUL-24 h-3	0.0915135090	5.9605387370	0.0552246910	0.0100775270	0.0000781000	0.0037862350	0.0148779420	0.2227296550	0.0010413900
INU-24 h-1	0.1232657030	5.2265353600	0.0295602630	0.0076120000	0.0001144900	0.0035738670	0.0152513690	0.2333029620	0.0006141040
INU-24 h-2	0.0856177920	3.2255576610	0.0287400250	0.0100861330	0.0000230000	0.0038536560	0.0129936660	0.2261196870	0.0006714810
INU-24 h-3	0.0915135090	5.9605387370	0.0552246910	0.0100775270	0.0000781000	0.0037862350	0.0148779420	0.2227296550	0.0010413900
CUL-48 h-1	N/A	3.2231463030	0.0455557810	0.0158257280	0.0001692860	0.0026313810	0.0028442720	0.0521553490	N/A
CUL-48 h-2	N/A	3.1476853000	0.0520512210	0.0144768100	0.0002437200	0.0036448070	0.0049481400	0.0623292640	N/A
CUL-48 h-3	N/A	3.7150037200	0.0480525920	0.0148268230	0.0004156160	0.0036877540	0.0043449840	0.0646755950	N/A
INU-48 h-1	0.0193850560	4.1624035160	0.0340967460	0.0163341930	0.0001297760	0.0059731720	0.0077219450	0.1496989710	0.0009127750
INU-48 h-2	0.0138224020	3.3379634520	0.0503051790	0.0161054270	0.0001366110	0.0057730960	0.0068944520	0.2011700240	0.0012561000
INU-48 h-3	0.0228740120	3.5631328700	0.0378962170	0.0118196160	0.0001426150	0.0058849220	0.0085255970	0.2015779420	0.0011038600
CUL-72 h-1	N/A	3.3875600690	0.0435714670	0.0078171270	0.0002062110	0.0006408090	0.0022992860	0.0170817220	N/A
CUL-72 h-2	N/A	5.0054442210	0.0315009500	0.0083377090	0.0003376210	0.0040045910	0.0019939520	0.0440173210	N/A
CUL-72 h-3	N/A	4.5221174920	0.0504605090	0.0083746700	0.0002414740	0.0021536450	0.0033064940	0.0334014080	N/A
INU-72 h-1	N/A	4.7117764200	0.0369384030	0.0118828670	0.0002517730	0.0047675880	0.0026479910	0.0463073730	N/A
INU-72 h-2	N/A	2.5656322580	0.0686219910	0.0163654390	0.0003318240	0.0082437710	0.0039618810	0.0782190270	N/A
INU-72 h-3	N/A	4.0206375000	0.0372110460	0.0099327300	0.0002506580	0.0053424350	0.0036532880	0.0599716120	N/A

Sample name	Homocysteine	Homocystine	Hydroxytyrosol	Hyodeoxycholic acid	Hypoxanthine	Indole	Indole-3-acetic acid	Inosine	Isobutyric acid	Isocitric acid	Isoleucine	Isovaleric acid	Kynurenine	L-Cysteine S-sulfate
CUL-24 h-1	N/A	N/A	N/A	N/A	0.0782789620	0.0554661140	0.0015370230	0.0146680600	0.4879464950	N/A	26.6417881500	0.6305545130	0.0029817290	N/A
CUL-24 h-2	N/A	N/A	N/A	N/A	0.1204200580	0.0237576610	0.0013255060	0.0259283530	0.7231929160	N/A	21.6233360800	0.9345420100	0.0027300260	N/A
CUL-24 h-3	N/A	N/A	N/A	N/A	0.1915800370	0.0273991780	0.0009982530	0.0218288490	0.6144581710	N/A	25.9138387000	0.7505140790	0.0035750910	N/A
INU-24 h-1	N/A	N/A	N/A	N/A	0.0782789620	0.0554661140	0.0015370230	0.0146680600	0.4879464950	N/A	26.6417881500	0.6305545130	0.0029817290	N/A
INU-24 h-2	N/A	N/A	N/A	N/A	0.1204200580	0.0237576610	0.0013255060	0.0259283530	0.7231929160	N/A	21.6233360800	0.9345420100	0.0027300260	N/A
INU-24 h-3	N/A	N/A	N/A	N/A	0.1915800370	0.0273991780	0.0009982530	0.0218288490	0.6144581710	N/A	25.9138387000	0.7505140790	0.0035750910	N/A
CUL-48 h-1	N/A	N/A	N/A	N/A	0.2433609730	0.1535144210	0.0015922120	0.0635233220	1.1888084690	N/A	15.0888896200	1.4915823280	0.0042890030	N/A
CUL-48 h-2	N/A	N/A	N/A	N/A	0.2523780990	0.1728714680	0.0014454040	0.0508645700	0.9469981410	N/A	3.0089607090	1.1418611800	0.0046504600	N/A
CUL-48 h-3	N/A	N/A	N/A	N/A	0.2790594000	0.2005802950	0.0047356210	0.0661009420	1.0636737350	N/A	21.6158420100	1.2734288190	0.0063093810	N/A
INU-48 h-1	N/A	N/A	N/A	N/A	0.0825954120	0.0656009860	0.0010977690	0.0315501770	1.2041498710	N/A	67.6580724700	1.0741487990	0.0035517770	N/A
INU-48 h-2	N/A	N/A	N/A	N/A	0.1204751210	0.0814634520	0.0046910240	0.0374498840	1.2129832670	N/A	38.7852322800	1.4044760020	0.0037760300	N/A
INU-48 h-3	N/A	N/A	N/A	N/A	0.1205354430	0.1292476660	0.0021650410	0.0216663700	1.0009776380	N/A	49.3929331300	1.1300727310	0.0036355260	N/A
CUL-72 h-1	N/A	N/A	N/A	N/A	0.0209191290	0.1861854260	0.0054734660	0.0117349540	1.7845294620	N/A	0.0888709950	2.1564802630	0.0036516550	N/A
CUL-72 h-2	N/A	N/A	N/A	N/A	0.1014031980	0.0549160540	0.0057003820	0.0140080190	1.5981551300	N/A	0.1136763750	2.0091022870	0.0031594640	N/A
CUL-72 h-3	N/A	N/A	N/A	N/A	0.0654004740	0.1405400880	0.0040268830	0.0123705690	2.2384522430	N/A	0.0851840840	2.8730197860	0.0032551680	N/A
INU-72 h-1	N/A	N/A	N/A	N/A	0.1143403670	0.0689608280	0.0006923160	0.0194391700	0.7900961060	N/A	29.8642885400	0.9706812780	0.0035618140	N/A
INU-72 h-2	N/A	N/A	N/A	N/A	0.1147673410	0.0738494380	0.0015650830	0.0219417960	1.3596613700	N/A	35.5514740800	1.6502810920	0.0046034870	N/A
INU-72 h-3	N/A	N/A	N/A	N/A	0.0379384470	0.0484365460	0.0033429560	0.0123688290	1.8703868420	N/A	59.1495197400	2.3554500000	0.0041175740	N/A

Table S13 (continued)

Sample name	Leucine	Lithocholic acid	Lysine	Maleic acid	Malic acid	Malonic acid	Methionine	Methionine sulfoxide	Methyl sulfate	Methyldopa	Mevalonic acid	N1,N12-Diacetylspermine
CUL-24 h-1	45.3102512400	0.0314651050	4.5862515510	0.4311850960	0.0034358070	0.0286434170	0.2111848640	0.0008973770	N/A	N/A	0.0031947470	0.0034147930
CUL-24 h-2	32.3463138400	0.0328362600	4.5216268530	0.3452909390	0.0028790660	0.0445362600	0.2723850490	0.0008888740	N/A	N/A	0.0037047550	0.0045092300
CUL-24 h-3	36.1753011100	0.0253669680	5.2735961910	0.2710168460	0.0054245440	0.0415537030	0.2986518550	0.0008235820	N/A	N/A	0.0045311120	0.0035257260
INU-24 h-1	45.3102512400	0.0314651050	4.5862515510	0.4311850960	0.0034358070	0.0286434170	0.2111848640	0.0008973770	N/A	N/A	0.0031947470	0.0034147930
INU-24 h-2	32.3463138400	0.0328362600	4.5216268530	0.3452909390	0.0028790660	0.0445362600	0.2723850490	0.0008888740	N/A	N/A	0.0037047550	0.0045092300
INU-24 h-3	36.1753011100	0.0253669680	5.2735961910	0.2710168460	0.0054245440	0.0415537030	0.2986518550	0.0008235820	N/A	N/A	0.0045311120	0.0035257260
CUL-48 h-1	15.2208704800	0.0588462110	4.9850222860	0.1845038020	0.0019602080	0.0368951100	0.1836649190	0.0008507910	N/A	N/A	0.0084760820	0.0078345370
CUL-48 h-2	1.0960213190	0.0472115520	4.1581600150	0.1872245910	0.0048020570	0.0393668320	0.2180976080	0.0010341580	N/A	N/A	0.0136962070	0.0074464920
CUL-48 h-3	35.5453373000	0.0568205230	4.7514397320	0.1746392610	0.0039972560	0.0575700640	0.2582582470	0.0009483890	N/A	N/A	0.0164130270	0.0084395830
INU-48 h-1	96.0344661200	0.0365645480	16.0195540300	0.0441590370	0.0040005420	0.0339441470	0.1341328800	0.0007295790	N/A	N/A	0.0112859990	0.0068055480
INU-48 h-2	72.4416006200	0.0539052070	10.8330306000	0.1648784680	0.0020491260	0.0413531480	0.1318540840	0.0009075590	N/A	N/A	0.0122805810	0.0054165820
INU-48 h-3	66.4995657800	0.0363866480	18.1683836300	0.0781917390	0.0028802220	0.0236458100	0.1831043750	0.0006765170	N/A	N/A	0.0063053730	0.0072908220
CUL-72 h-1	0.6557648740	0.0832781750	1.9483681350	0.2639485130	0.0017220450	0.0483073370	0.0378156460	0.0001144540	N/A	N/A	0.0058681640	0.0064596790
CUL-72 h-2	0.6927343940	0.0767209360	4.0326220640	0.1561721260	0.0014533610	0.0310003240	0.1222993670	0.0005537960	N/A	N/A	0.0116684720	0.0113283610
CUL-72 h-3	0.5669663570	0.1667937940	1.8762728800	0.3112899590	0.0006250850	0.0698876640	0.1070648040	0.0002167250	N/A	N/A	0.0274742200	0.0107160510
INU-72 h-1	19.0181946400	0.0457274020	6.8276333370	0.0810335200	0.0047459140	0.0571630470	0.2260436250	0.0005972260	N/A	N/A	0.0127876880	0.0076425080
INU-72 h-2	45.7614284200	0.0699388200	5.5071618980	0.1265800120	0.0041811270	0.0473156010	0.1539069760	0.0008271260	N/A	N/A	0.0199171640	0.0079362530
INU-72 h-3	93.7780263200	0.0768661180	7.2682828950	0.1236865390	0.0004743870	0.0910674740	0.0871985530	0.0005714900	N/A	N/A	0.0201485920	0.0064577890

Sample name	N1,N8-Diacetylspermidine	N1-Acetylspermidine	N1-Acetylspermine	N2-Phenylacetylglutamine	N-Acetyl-L-valine	N-Carbamoyl-L-aspartic acid	Nicotinamide	Nicotinamide adenine dinucleotide	Nicotinic acid	Noradrenaline
CUL-24 h-1	0.0045093360	0.0371503800	0.0036914200	N/A	0.4296960300	0.0001766730	1.4185150430	4.6577116940	0.6394684400	N/A
CUL-24 h-2	0.0052774840	0.0550943160	0.0047576850	N/A	0.3722989290	0.0001410900	1.2334271000	2.7191243820	0.4804040360	N/A
CUL-24 h-3	0.0071229980	0.0685452470	0.0051362060	N/A	0.3723477780	0.0000635000	1.1564501950	4.3017333980	0.5325919600	N/A
INU-24 h-1	0.0045093360	0.0371503800	0.0036914200	N/A	0.4296960300	0.0001766730	1.4185150430	4.6577116940	0.6394684400	N/A
INU-24 h-2	0.0052774840	0.0550943160	0.0047576850	N/A	0.3722989290	0.0001410900	1.2334271000	2.7191243820	0.4804040360	N/A
INU-24 h-3	0.0071229980	0.0685452470	0.0051362060	N/A	0.3723477780	0.0000635000	1.1564501950	4.3017333980	0.5325919600	N/A
CUL-48 h-1	0.0080624870	0.0574853830	0.0225236890	N/A	1.7423394070	0.0016550120	1.2341105140	3.1352517040	0.3652592420	N/A
CUL-48 h-2	0.0109337510	0.0479827650	0.0206987730	N/A	1.2232672290	0.0032613890	1.0254598410	2.8827169060	0.3785672410	N/A
CUL-48 h-3	0.0091968690	0.0523979480	0.0212304070	N/A	1.6340178570	0.0011385600	1.1113430060	3.8100930060	0.3650897200	N/A
INU-48 h-1	0.0069552850	0.0866208190	0.0093487560	N/A	2.3900798670	0.0003778190	1.7497126930	4.3357102270	0.6131871780	N/A
INU-48 h-2	0.0104126710	0.0920000550	0.0082009140	N/A	1.2402741080	0.0002132710	0.9822996480	3.8347710260	0.3843616800	N/A
INU-48 h-3	0.0092714010	0.0893430850	0.0082193880	N/A	1.3110513460	0.0003225060	1.0307940730	4.1512407730	0.4160123210	N/A
CUL-72 h-1	0.0053645140	0.0407050200	0.0193387010	N/A	1.1902295480	0.0048353010	0.5857791050	1.2011670480	0.1173914470	N/A
CUL-72 h-2	0.0069783970	0.0343264220	0.0181636900	N/A	1.4568734550	0.0054705440	1.2702649880	3.4600015450	0.1884418260	N/A
CUL-72 h-3	0.0048154490	0.0396908510	0.0287893140	N/A	0.7361255320	0.0065543630	0.8543237630	2.1214423820	0.1276267240	N/A
INU-72 h-1	0.0047565020	0.0296236830	0.0066345300	N/A	2.2055672370	0.0009093260	1.0489047350	2.4037916830	0.6379304370	N/A
INU-72 h-2	0.0067363810	0.0367419240	0.0092452890	N/A	1.9897302580	0.0005760110	0.7792190140	1.6330104520	0.4357999890	N/A
INU-72 h-3	0.0062611840	0.0669293420	0.0138307240	N/A	2.0708276320	0.0003657580	0.7602756580	0.6148610530	0.5343450660	N/A

Table S13 (continued)

Sample name	Norspermidine	N-α-Benzoylarginine ethylester	N-γ-Ethyl-L-glutamine	N-ε-Acetyl-L-lysine	Ophthalmic acid	Ornithine	Orotic acid	Oxidized glutathione	Pantothenic acid	Phenylalanine
CUL-24 h-1	N/A	0.0000133000	0.0059356350	N/A	0.0515923150	25.6952776100	N/A	0.0013667280	0.0139200920	28.7853520500
CUL-24 h-2	N/A	0.0000083400	0.0048282580	N/A	0.0335395350	14.4232619400	N/A	0.0014837600	0.0141682700	18.5805477800
CUL-24 h-3	N/A	0.0000101000	0.0062531900	N/A	0.0352350260	17.6053182000	N/A	0.0015615920	0.0149364220	21.5678670200
INU-24 h-1	N/A	0.0000133000	0.0059356350	N/A	0.0515923150	25.6952776100	N/A	0.0013667280	0.0139200920	28.7853520500
INU-24 h-2	N/A	0.0000083400	0.0048282580	N/A	0.0335395350	14.4232619400	N/A	0.0014837600	0.0141682700	18.5805477800
INU-24 h-3	N/A	0.0000101000	0.0062531900	N/A	0.0352350260	17.6053182000	N/A	0.0015615920	0.0149364220	21.5678670200
CUL-48 h-1	N/A	0.0000197000	0.0112526870	N/A	0.0193094450	1.5862893290	N/A	N/A	0.0157179010	27.1594782400
CUL-48 h-2	N/A	0.0000164000	0.0104443850	N/A	0.0133331930	4.1541683190	N/A	N/A	0.0294059430	0.7986836890
CUL-48 h-3	N/A	0.0000162000	0.0113358690	N/A	0.0161113650	1.3094630460	N/A	N/A	0.0356780380	21.7595176100
INU-48 h-1	N/A	0.0000126000	0.0048724020	N/A	0.0416986970	2.6595433100	N/A	N/A	0.0133511420	58.9683211800
INU-48 h-2	N/A	0.0000128000	0.0066280000	N/A	0.0099419530	5.1610744170	N/A	N/A	0.0009624740	45.8580030800
INU-48 h-3	N/A	0.0000115000	0.0077098350	N/A	0.0111864090	2.0945902080	N/A	N/A	0.0008130890	38.3806882300
CUL-72 h-1	N/A	0.0000158000	0.0144403680	N/A	0.0064760780	0.4415150170	N/A	N/A	0.0007936480	0.5801618990
CUL-72 h-2	N/A	0.0000211000	0.0237929390	N/A	0.0119744280	0.9187724040	N/A	N/A	0.0122130100	0.8593943140
CUL-72 h-3	N/A	0.0000230000	0.0227658550	N/A	0.0124126220	1.2724204050	N/A	N/A	0.0647190160	0.5986186030
INU-72 h-1	N/A	0.0000149000	0.0083986990	N/A	0.0095753640	2.3243419750	N/A	N/A	0.0312303640	0.5682619850
INU-72 h-2	N/A	0.0000150000	0.0101376760	N/A	0.0058521280	0.7328949800	N/A	N/A	0.0050534150	6.6604993670
INU-72 h-3	N/A	0.0000168000	0.0118168550	N/A	0.0143461450	2.0819453950	N/A	N/A	0.0009529720	56.8792763200

Sample name	Phenyllactic acid	Phenylpyruvic acid	Phe-Phe	Picolinic acid butyl ester	Pipecolic acid	Proline	Proline betaine	Propionic acid	Prostaglandin E2	Protocatechualdehyde	Protocatechuic acid
CUL-24 h-1	0.0786833510	0.0085224800	0.0004072850	0.0000059400	N/A	22.9617051800	0.0003835030	7.8317385240	N/A	N/A	0.0170538770
CUL-24 h-2	0.0736426280	0.0051477680	0.0003786560	0.0000046400	N/A	13.6071787500	0.0003885830	10.9972075800	N/A	N/A	0.0251079940
CUL-24 h-3	0.1016075850	0.0072326290	0.0004076770	0.0000044100	N/A	26.3601928700	0.0005125240	9.0258789060	N/A	N/A	0.0471343180
INU-24 h-1	0.0786833510	0.0085224800	0.0004072850	0.0000059400	N/A	22.9617051800	0.0003835030	7.8317385240	N/A	N/A	0.0170538770
INU-24 h-2	0.0736426280	0.0051477680	0.0003786560	0.0000046400	N/A	13.6071787500	0.0003885830	10.9972075800	N/A	N/A	0.0251079940
INU-24 h-3	0.1016075850	0.0072326290	0.0004076770	0.0000044100	N/A	26.3601928700	0.0005125240	9.0258789060	N/A	N/A	0.0471343180
CUL-48 h-1	0.2986267040	0.0032822780	0.0002641550	0.0000074600	N/A	0.6150407050	0.0007438690	17.1694022000	N/A	N/A	0.0205800730
CUL-48 h-2	0.2641274170	0.0000989000	0.0001926940	0.0000065300	N/A	0.6467866880	0.0010571700	14.3712320300	N/A	N/A	0.0322074000
CUL-48 h-3	0.4012707710	0.0027599130	0.0002175650	0.0000061200	N/A	0.6063617930	0.0010235790	15.6889384900	N/A	N/A	0.0396292100
INU-48 h-1	0.2213358700	0.0058284790	0.0003522980	0.0000051700	N/A	3.8920073970	0.0006508650	13.3541380800	N/A	N/A	0.0155795400
INU-48 h-2	0.1909974680	0.0046007770	0.0003796450	0.0000045000	N/A	0.4682211580	0.0006292560	17.1242844600	N/A	N/A	0.0041177850
INU-48 h-3	0.2211849760	0.0052513300	0.0003341310	0.0000050700	N/A	0.7035138950	0.0007220320	13.5641120300	N/A	N/A	0.0036446870
CUL-72 h-1	0.2600781610	0.0006222190	0.0002285300	0.0000064100	N/A	0.1917914760	0.0015204420	25.3417763200	N/A	N/A	0.0074599900
CUL-72 h-2	0.2529966010	0.0006620200	0.0002355620	0.0000069700	N/A	0.3360509890	0.0013675350	22.4230686000	N/A	N/A	0.0127434640
CUL-72 h-3	0.2745819640	0.0002385630	0.0002676040	0.0000078900	N/A	0.2518190090	0.0016361080	33.0687193900	N/A	N/A	0.0141540430
INU-72 h-1	0.1304779340	0.0008914220	0.0001990480	0.0000054100	N/A	0.3902705080	0.0009036480	11.3850930100	N/A	N/A	0.0037695090
INU-72 h-2	0.3835072450	0.0011530960	0.0002370900	0.0000051100	N/A	0.2565782700	0.0009987710	19.6175570100	N/A	N/A	0.0020368880
INU-72 h-3	0.3442484870	0.0032784880	0.0003973370	0.0000053700	N/A	0.3108385530	0.0009381030	27.9314868400	N/A	N/A	0.0025979160

Table S13 (continued)

Sample name	Putrescine	Pyridoxal 5'-phosphoric acid	Pyridoxine	Pyruvic acid	Riboflavin	Saccharic acid	S-Adenosylhomocysteine	S-Adenosylmethionine	Salicylic acid	Serine
CUL-24 h-1	1.6856052260	0.0192895980	0.0000230000	0.0387786910	0.0036605590	0.0041649780	0.0018304590	0.0147139890	0.0089876550	0.2246584600
CUL-24 h-2	1.4421252060	0.0137628580	0.0000188000	0.0126799090	0.0038409750	0.0041201850	0.0016210480	0.0135044810	0.0083933770	0.0359054610
CUL-24 h-3	1.6045402020	0.0180665040	0.0000308000	0.0165598840	0.0046527060	0.0038903150	0.0026459890	0.0151475670	0.0102974040	0.0542193600
INU-24 h-1	1.6856052260	0.0192895980	0.0000230000	0.0387786910	0.0036605590	0.0041649780	0.0018304590	0.0147139890	0.0089876550	0.2246584600
INU-24 h-2	1.4421252060	0.0137628580	0.0000188000	0.0126799090	0.0038409750	0.0041201850	0.0016210480	0.0135044810	0.0083933770	0.0359054610
INU-24 h-3	1.6045402020	0.0180665040	0.0000308000	0.0165598840	0.0046527060	0.0038903150	0.0026459890	0.0151475670	0.0102974040	0.0542193600
CUL-48 h-1	4.7582977190	0.0375166490	0.0001145540	0.0169854220	0.0081953660	0.0030395210	0.0020586770	0.0114506620	0.0163235840	0.0190152140
CUL-48 h-2	3.9405726330	0.0501144770	0.0000465000	0.0154507620	0.0112653140	0.0026637670	0.0027635480	0.0138518470	0.0197969940	0.0228090050
CUL-48 h-3	4.0824435760	0.0490273070	0.0001396900	0.0171886220	0.0063777030	0.0033015170	0.0025240170	0.0101394970	0.0213657800	0.0225177210
INU-48 h-1	6.3471751720	0.0240496360	0.0000590000	0.0188408930	0.0061303330	0.0059755310	0.0014091340	0.0106465430	0.0141263720	0.0167094230
INU-48 h-2	5.3017167550	0.0148941710	0.0000598000	0.0202213120	0.0054724370	0.0055142940	0.0020563580	0.0134595000	0.0161650370	0.0195440060
INU-48 h-3	3.6352594440	0.0130079730	0.0000802000	0.0198082610	0.0060473570	0.0055423040	0.0020012150	0.0125677590	0.0156492560	0.0336475850
CUL-72 h-1	6.0767791760	0.0300981620	0.0001277010	0.0131184210	0.0151065570	0.0014230110	0.0011911930	0.0027872180	0.0228679490	0.0063158390
CUL-72 h-2	5.7908621760	0.0662137210	0.0001295830	0.0135872370	0.0081883810	0.0025166390	0.0020151910	0.0058320130	0.0256568370	0.0099780590
CUL-72 h-3	6.4172837720	0.0525142510	0.0001307960	0.0106466470	0.0136893210	0.0026064210	0.0011260110	0.0047879170	0.0248300870	0.0100336020
INU-72 h-1	5.1440827230	0.0275814620	0.0001107440	0.0199933390	0.0071934870	0.0058098080	0.0019405320	0.0086840990	0.0172652320	0.1483068850
INU-72 h-2	4.2770619990	0.0135330710	0.0001558910	0.0211906940	0.0105656740	0.0057988730	0.0016359570	0.0074646000	0.0230506890	0.0497473470
INU-72 h-3	7.0503421050	0.0164186910	0.0002150350	0.0227556710	0.0074980390	0.0075108680	0.0009603410	0.0039993390	0.0187579410	0.0095752110

Sample name	Serine O-sulfate	Serotonin	Shikimic acid	Sinapic acid	Sorbitol 6-phosphate	Spermidine	Spermine	Succinic acid	Symmetric dimethylarginine	Taurine	Taurocholic acid
CUL-24 h-1	0.0017427640	0.0000971000	N/A	N/A	N/A	0.0462499610	0.0040115660	1.2019478910	N/A	N/A	N/A
CUL-24 h-2	0.0016552690	0.0001314750	N/A	N/A	N/A	0.0533206340	0.0028900750	1.1822186990	N/A	N/A	N/A
CUL-24 h-3	0.0012346590	0.0001421470	N/A	N/A	N/A	0.0588723140	0.0030995840	1.5986649580	N/A	N/A	N/A
INU-24 h-1	0.0017427640	0.0000971000	N/A	N/A	N/A	0.0462499610	0.0040115660	1.2019478910	N/A	N/A	N/A
INU-24 h-2	0.0016552690	0.0001314750	N/A	N/A	N/A	0.0533206340	0.0028900750	1.1822186990	N/A	N/A	N/A
INU-24 h-3	0.0012346590	0.0001421470	N/A	N/A	N/A	0.0588723140	0.0030995840	1.5986649580	N/A	N/A	N/A
CUL-48 h-1	0.0003646240	0.0002284550	N/A	N/A	N/A	0.0669450050	0.0047395250	0.8785808860	N/A	N/A	N/A
CUL-48 h-2	0.0004036490	0.0002350990	N/A	N/A	N/A	0.0567416960	0.0050038240	0.9722632620	N/A	N/A	N/A
CUL-48 h-3	0.0005317220	0.0006296270	N/A	N/A	N/A	0.0686666670	0.0049125960	1.1774906990	N/A	N/A	N/A
INU-48 h-1	0.0003509580	0.0002116030	N/A	N/A	N/A	0.0349606780	0.0019642540	0.4304367500	N/A	N/A	N/A
INU-48 h-2	0.0004734660	0.0001671580	N/A	N/A	N/A	0.0351144980	0.0016720540	0.3980173930	N/A	N/A	N/A
INU-48 h-3	0.0004868040	0.0004246500	N/A	N/A	N/A	0.0297032780	0.0016205940	0.5236293420	N/A	N/A	N/A
CUL-72 h-1	N/A	0.0003019330	N/A	N/A	N/A	0.1226696940	0.0057670370	0.2253901600	N/A	N/A	N/A
CUL-72 h-2	N/A	0.0002058770	N/A	N/A	N/A	0.0301080110	0.0031033120	0.6518108000	N/A	N/A	N/A
CUL-72 h-3	N/A	0.0002290710	N/A	N/A	N/A	0.0787769080	0.0049781890	0.1719821470	N/A	N/A	N/A
INU-72 h-1	N/A	0.0001804140	N/A	N/A	N/A	0.0117320840	0.0011096530	0.4004716260	N/A	N/A	N/A
INU-72 h-2	N/A	0.0003324470	N/A	N/A	N/A	0.0147906660	0.0012810960	0.2227058700	N/A	N/A	N/A
INU-72 h-3	N/A	0.0003090730	N/A	N/A	N/A	0.0235820000	0.0022021380	0.1175640790	N/A	N/A	N/A

Table S13 (continued)

Sample name	Thiamine	Threonine	Thymidine	Thymidine 5'-monophosphoric acid	Thymine	Trimethylamine	Trimethylamine N-oxide	Tryptamine	Tryptophan	Tyramine
CUL-24 h-1	0.0007017010	0.0173959020	0.0062978750	0.0018167430	0.0026843180	0.0047982090	N/A	0.4733599570	7.0320758370	5.1317075060
CUL-24 h-2	0.0005660770	0.0076198480	0.0035145410	0.0015361590	0.0039432830	0.0042381300	N/A	0.5594728170	6.8815197690	4.0493002470
CUL-24 h-3	0.0006280140	0.0099659830	0.0057299560	0.0018930090	0.0062120200	0.0060407430	N/A	0.5985640460	7.4806762700	4.3224975590
INU-24 h-1	0.0007017010	0.0173959020	0.0062978750	0.0018167430	0.0026843180	0.0047982090	N/A	0.4733599570	7.0320758370	5.1317075060
INU-24 h-2	0.0005660770	0.0076198480	0.0035145410	0.0015361590	0.0039432830	0.0042381300	N/A	0.5594728170	6.8815197690	4.0493002470
INU-24 h-3	0.0006280140	0.0099659830	0.0057299560	0.0018930090	0.0062120200	0.0060407430	N/A	0.5985640460	7.4806762700	4.3224975590
CUL-48 h-1	0.0012548400	0.0085226600	0.0154566790	0.0038821350	0.0075444610	0.0060773000	N/A	0.9478887000	0.1522690740	8.2698217090
CUL-48 h-2	0.0012073060	0.0090603560	0.0097719700	0.0059302720	0.0066004150	0.0051066790	N/A	0.7943170550	0.1220537310	6.7807325240
CUL-48 h-3	0.0012126580	0.0088687000	0.0142380080	0.0063113780	0.0100758490	0.0050614770	N/A	2.6982694690	0.1527041170	8.6094122020
INU-48 h-1	0.0010550680	0.0089341070	0.0083751390	0.0026528000	0.0063907800	0.0019247700	N/A	0.6281844980	1.8774056600	11.9282161200
INU-48 h-2	0.0012664380	0.0089402080	0.0070126870	0.0017709400	0.0056139150	0.0026625570	N/A	0.8358135180	0.1556454760	9.3667657420
INU-48 h-3	0.0012631750	0.0095525080	0.0079937310	0.0014636960	0.0060743000	0.0024526910	N/A	2.1320310460	0.1210407080	8.9204190190
CUL-72 h-1	0.0013662110	0.0013998820	0.0046282380	0.0095292260	0.0088849970	0.0074569580	N/A	0.8661556060	0.0848997430	8.2051415900
CUL-72 h-2	0.0028540540	0.0079954500	0.0036212040	0.0159825480	0.0057608850	0.0023694670	N/A	0.6933882880	0.1501745210	11.2053924600
CUL-72 h-3	0.0021886320	0.0037754770	0.0022033030	0.0093285320	0.0080535500	0.0033064150	N/A	0.7652103470	0.0812997070	7.6213489300
INU-72 h-1	0.0016627120	0.0931774440	0.0106801710	0.0068468980	0.0078270710	0.0014057110	N/A	0.6414246490	0.1112303950	9.3964414700
INU-72 h-2	0.0015068540	0.0111999910	0.0134515220	0.0057626790	0.0095589760	0.0015665310	N/A	0.7041919610	0.1044137330	8.2684095230
INU-72 h-3	0.0016730990	0.0097219470	0.0066028290	0.0042083230	0.0099983360	0.0013449550	N/A	0.9377348680	0.1704870390	13.4790197400

Sample name	Tyrosine	Uracil	Uric acid	Uridine	Urocanic acid	Ursodeoxycholic acid	Valeric acid	Valine	Vanillic acid	Vanillin	Vanillylmandelic acid	Xanthine	α-Methylbenzylamine	γ-Butyrobetaine
CUL-24 h-1	1.6343854680	0.6259983720	0.1141743180	0.0704822040	0.0835497830	N/A	0.3353884930	56.0300093100	0.0012006880	N/A	N/A	0.1090861900	0.0125568940	0.2822238680
CUL-24 h-2	1.7337792420	0.6799246290	0.0758030890	0.1004607080	0.0661411860	N/A	0.4873869030	38.4496952200	0.0013993950	N/A	N/A	0.1266762360	0.0087724880	0.2237924630
CUL-24 h-3	2.0730896000	0.6913065590	0.1556579180	0.0890348310	0.1071544190	N/A	0.3856758630	42.4780273400	0.0013411610	N/A	N/A	0.1854178470	0.0114752850	0.1987537840
INU-24 h-1	1.6343854680	0.6259983720	0.1141743180	0.0704822040	0.0835497830	N/A	0.3353884930	56.0300093100	0.0012006880	N/A	N/A	0.1090861900	0.0125568940	0.2822238680
INU-24 h-2	1.7337792420	0.6799246290	0.0758030890	0.1004607080	0.0661411860	N/A	0.4873869030	38.4496952200	0.0013993950	N/A	N/A	0.1266762360	0.0087724880	0.2237924630
INU-24 h-3	2.0730896000	0.6913065590	0.1556579180	0.0890348310	0.1071544190	N/A	0.3856758630	42.4780273400	0.0013411610	N/A	N/A	0.1854178470	0.0114752850	0.1987537840
CUL-48 h-1	0.4951206080	1.1062775300	0.2658944020	0.0633185830	0.0113498300	N/A	0.7474077080	67.9218012600	0.0017310520	N/A	N/A	0.2278913210	0.0443879260	1.2133495020
CUL-48 h-2	0.2817121960	0.5796781730	0.4710640800	0.0571843460	0.0231493930	N/A	0.6020495790	36.0714613300	0.0028778350	N/A	N/A	0.3160503220	0.0439578210	1.0303414720
CUL-48 h-3	0.3390497270	0.6237121780	0.4293922370	0.0522300660	0.0238707960	N/A	0.6694345240	61.9202194900	0.0029398250	N/A	N/A	0.2748190100	0.0334061010	0.8409480410
INU-48 h-1	1.1573531300	0.9352765870	0.0836298780	0.0846771010	0.0357934440	N/A	0.5371725990	117.3366209000	0.0015118070	N/A	N/A	0.2828942970	0.0228652180	1.0655644300
INU-48 h-2	0.5445161820	0.7240488770	0.0748932190	0.0911335310	0.0513024160	N/A	0.7450911490	81.6616028200	0.0004483850	N/A	N/A	0.3291103590	0.0183415070	0.7759142450
INU-48 h-3	0.8254917500	0.7796374290	0.0921570230	0.0997256840	0.0695021710	N/A	0.5933328270	82.6874185800	0.0017837140	N/A	N/A	0.3868412400	0.0171671730	0.7059167390
CUL-72 h-1	0.1622081660	0.2901524600	0.2273975970	0.0100336600	0.0096772670	N/A	1.1668113560	0.9953296620	0.0050478220	N/A	N/A	0.4219919910	0.0370322150	0.8081164190
CUL-72 h-2	0.2131011280	0.5679560410	0.3201063810	0.0487363410	0.0195875150	N/A	1.0419100740	0.9456489490	0.0029533070	N/A	N/A	0.5321217550	0.0528183100	1.9308181400
CUL-72 h-3	0.2144040020	0.3633525070	0.3010901650	0.0341193770	0.0178067640	N/A	1.4887251870	0.7691501510	0.0033019650	N/A	N/A	0.4460844450	0.0626078640	1.0807859630
INU-72 h-1	0.1771962200	0.8482881460	0.2575079780	0.0716501950	0.0120512940	N/A	0.5416004110	66.3656491200	0.0019547300	N/A	N/A	0.1309054650	0.0411041150	1.4574423610
INU-72 h-2	0.2840839450	0.7745598870	0.3354637350	0.1113504940	0.0154693310	N/A	0.8575925100	75.4029640000	0.0011981210	N/A	N/A	0.2142388090	0.0288396060	0.9371304900
INU-72 h-3	0.8296789470	0.8455118420	0.1712265130	0.0502358680	0.0176074080	N/A	1.2982284210	125.6688816000	0.0028101220	N/A	N/A	0.1684413160	0.0281744610	0.7091190790

Table S13 (continued)

Extracellular results (µM)

Sample name	1-Deoxy-D-xylulose 5-phosphoric acid	1-Methyl-2-pyrrolidinone	2,3-Pyridinedicarboxylic acid	2,6-Diaminopimelic acid	2-Aminobutyric acid	2-C-Methyl-D-erythritol 4-phosphoric acid	2'-Deoxyinosine	2-Hydroxyglutaric acid
CUL-72 h-1	N/A	14.54849	N/A	2.699704	253.4538	N/A	N/A	N/A
CUL-72 h-2	N/A	24.27724	N/A	5.053381	269.7154	N/A	N/A	N/A
CUL-72 h-3	N/A	16.26349	N/A	3.178681	289.409	N/A	N/A	N/A
INU-72 h-1	N/A	12.54202	N/A	7.268976	2509.289	N/A	N/A	560.8563
INU-72 h-2	N/A	15.92628	N/A	9.419192	773.4284	N/A	N/A	636.5216
INU-72 h-3	N/A	13.52946	N/A	6.891001	1588.867	N/A	N/A	N/A

Sample name	2-Hydroxyisocaproic acid	2-Hydroxypentanoic acid	2-Isopropylmalic acid	2-Oxoglutaric acid	2-Oxobutyric acid	2-Oxoisocaproic acid	2-Oxoisopentanoic acid	2-Phenylethylamine	3-(4-Hydroxyphenyl)propionic acid	3,4-Dihydroxyphenylacetaldehyde	3,4-Dihydroxyphenylacetic acid
CUL-72 h-1	1.352058	527179.4	N/A	0.1586649	2.66662	4908.625	N/A	180.2272	119.18	N/A	N/A
CUL-72 h-2	1.743327	774125.4	N/A	0.1138021	3.20254	3089.963	N/A	313.8633	94.95343	N/A	N/A
CUL-72 h-3	1.445856	4694.89	N/A	0.1677678	2.43207	1258.951	N/A	196.5451	119.5013	N/A	N/A
INU-72 h-1	9.011898	448597.3	N/A	0.05600501	2.595244	4515.645	N/A	269.5359	14.86664	N/A	N/A
INU-72 h-2	2.582412	442811.3	N/A	0.1229986	2.55349	4693.309	N/A	172.8463	34.02639	N/A	N/A
INU-72 h-3	0.9959482	78953.7	N/A	0.08016528	2.549809	2189.731	N/A	181.6586	21.81295	N/A	N/A

Sample name	3-Dehydroquinic acid	3-Dehydroshikimic acid	3-Hydroxybenzoic acid	3-Hydroxybutyric acid	3-Indoxylsulfuric acid	3-Phenylpropionic acid	3-Phosphoshikimic acid	4-Acetylbutyric acid	4-Aminobenzoic acid
CUL-72 h-1	N/A	N/A	1.300935	96.30945	N/A	5611.578	N/A	N/A	1603.033
CUL-72 h-2	N/A	N/A	1.397351	115.3187	N/A	7442.929	N/A	N/A	1351.563
CUL-72 h-3	N/A	N/A	1.444144	104.0646	N/A	6403.693	N/A	N/A	1842.261
INU-72 h-1	N/A	N/A	1.255833	99.84637	N/A	2991.402	N/A	N/A	1465.96
INU-72 h-2	N/A	N/A	1.513306	125.4726	N/A	3789.554	N/A	N/A	1123.885
INU-72 h-3	N/A	N/A	1.205449	87.83209	N/A	1747.518	N/A	N/A	1494.934

Sample name	4-Aminobutyric acid	4-Aminophenylalanine	4-Aminophenylpyruvic acid	4-Coumaric acid	4-Hydroxybenzoic acid	4-Hydroxymethylimidazole	4-Hydroxyphenylacetic acid	4-Hydroxyproline	4-Oxopentanoic acid
CUL-72 h-1	8548.406	N/A	N/A	N/A	22.57232	1.944355	6.939681	219.6654	2159.238
CUL-72 h-2	7506.982	N/A	N/A	N/A	22.64101	2.120802	4.771946	67.22042	2096.156
CUL-72 h-3	8612.671	N/A	N/A	N/A	32.82642	1.71923	6.510358	291.7546	2526.857
INU-72 h-1	7585.563	N/A	N/A	N/A	20.51147	1.963401	1.68707	370.0336	2047.832
INU-72 h-2	7027.345	N/A	N/A	N/A	27.12607	1.709129	2.558272	360.6155	2144.162
INU-72 h-3	8559.064	N/A	N/A	N/A	23.6427	2.234238	0.3772235	285.8335	2223.42

Sample name	5-Aminolevulinic acid	5-Aminovaleric acid	5'-Deoxyadenosine	5-Diphosphomevalonic acid	5-Glutamylcysteine	5'-Methylthioadenosine	5-Phosphomevalonic acid	6-Methylaminopurine	Acetylcarnitine
CUL-72 h-1	N/A	6179.687	N/A	N/A	N/A	N/A	N/A	N/A	N/A
CUL-72 h-2	N/A	5660.168	N/A	N/A	N/A	N/A	N/A	N/A	N/A
CUL-72 h-3	N/A	6042.992	N/A	N/A	N/A	N/A	N/A	N/A	N/A
INU-72 h-1	N/A	5157.745	N/A	N/A	N/A	N/A	N/A	N/A	N/A
INU-72 h-2	N/A	4954.818	N/A	N/A	N/A	N/A	N/A	N/A	N/A
INU-72 h-3	N/A	5820.745	N/A	N/A	N/A	N/A	N/A	N/A	N/A

Table S13 (continued)

Sample name	Acetylcholine	Aconitic acid	Adenine	Adenosine	Adenosine 3',5'-cyclic phosphoric acid	Adenosine 5'-monophosphoric acid	Adenylsuccinic acid	Adrenaline	AICAR	Alanine	Allantoin	Anthranilic acid
CUL-72 h-1	N/A	N/A	10.22483	1.505615	N/A	N/A	N/A	N/A	N/A	295.6021	N/A	5.952195
CUL-72 h-2	N/A	N/A	11.56916	1.769055	N/A	N/A	N/A	N/A	N/A	172.8334	N/A	4.644651
CUL-72 h-3	N/A	N/A	15.5902	1.762629	N/A	N/A	N/A	N/A	N/A	251.8843	N/A	5.510547
INU-72 h-1	N/A	N/A	12.85725	1.046473	N/A	N/A	N/A	N/A	N/A	1766.176	N/A	2.201295
INU-72 h-2	N/A	N/A	7.463034	1.177625	N/A	N/A	N/A	N/A	N/A	981.0241	N/A	1.055901
INU-72 h-3	N/A	N/A	11.49825	1.434194	N/A	N/A	N/A	N/A	N/A	3612.874	N/A	0.9693368

Sample name	Arginine	Argininosuccinic acid	Asparagine	Aspartic acid	Asymmetric dimethylarginine	Benzoic acid	Biotin	Butyric acid	Cadaverine	Caffeic acid	Carnitine
CUL-72 h-1	79.98734	N/A	34.82365	11.37849	0.4837101	15.94187	N/A	29766.17	6070.553	N/A	N/A
CUL-72 h-2	84.55428	N/A	12.72355	7.297097	1.297292	16.6167	N/A	49856.5	5376.412	N/A	N/A
CUL-72 h-3	67.06747	N/A	52.6034	7.584983	0.7635591	16.5193	N/A	39195.5	5595.146	N/A	N/A
INU-72 h-1	98.75862	N/A	46.91549	1.791212	3.723225	15.39515	N/A	30003.65	4873.881	N/A	N/A
INU-72 h-2	121.8236	N/A	50.46397	7.636384	2.864914	19.08706	N/A	33615.25	4648.497	N/A	N/A
INU-72 h-3	154.1827	N/A	48.21837	17.65031	6.515736	17.00343	N/A	34093.02	6503.04	N/A	N/A

Sample name	Carnosine	Catechol	Chenodeoxycholic acid	Cholic acid	Choline	Chorismic acid	Citicoline	Citric acid	Citrulline	Creatine	Creatinine	Crotonic acid	Cycloleucine
CUL-72 h-1	N/A	N/A	N/A	N/A	N/A	N/A	N/A	N/A	9.488812	N/A	1.060136	2.124692	33.22602
CUL-72 h-2	N/A	N/A	N/A	N/A	N/A	N/A	N/A	N/A	5.034508	N/A	0.7548009	1.497935	31.57546
CUL-72 h-3	N/A	N/A	N/A	N/A	N/A	N/A	N/A	N/A	6.501012	N/A	1.803609	1.740479	34.12451
INU-72 h-1	N/A	N/A	N/A	N/A	N/A	N/A	N/A	N/A	9.898307	N/A	3.18204	1.268161	24.00511
INU-72 h-2	N/A	N/A	N/A	N/A	N/A	N/A	N/A	N/A	6.741262	N/A	1.673363	1.464026	29.20273
INU-72 h-3	N/A	N/A	N/A	N/A	N/A	N/A	N/A	N/A	5.528568	N/A	7.77471	1.226785	26.79554

Sample name	Cystathionine	Cysteamine	Cysteine	Cysteine-glutathione disulphide	Cystine	Cytidine	Cytidine 3',5'-cyclic monophosphoric acid	Cytidine-5'-monophosphoric acid	Cytosine
CUL-72 h-1	N/A	N/A	N/A	N/A	N/A	0.1915274	N/A	N/A	N/A
CUL-72 h-2	N/A	N/A	N/A	N/A	N/A	0.1794119	N/A	N/A	N/A
CUL-72 h-3	N/A	N/A	N/A	N/A	N/A	0.2913068	N/A	N/A	N/A
INU-72 h-1	N/A	N/A	N/A	N/A	N/A	0.2579263	N/A	N/A	N/A
INU-72 h-2	N/A	N/A	N/A	N/A	N/A	0.2718323	N/A	N/A	N/A
INU-72 h-3	N/A	N/A	N/A	N/A	N/A	0.3341004	N/A	N/A	N/A

Sample name	Deoxycholic acid	Dimethylglycine	Dodecanoic acid	Dopa	Dopamine	Ergothioneine	Ferulic acid	Flavin adenine dinucleotide	Flavin mononucleotide	Folic acid	Fumaric acid	Glutamic acid	Glutamine
CUL-72 h-1	N/A	N/A	N/A	1.335497	1.0714	N/A	N/A	N/A	N/A	N/A	N/A	24.81542	2.482701
CUL-72 h-2	N/A	N/A	N/A	1.377728	1.400678	N/A	N/A	N/A	N/A	N/A	N/A	12.94854	1.045634
CUL-72 h-3	N/A	N/A	N/A	0.988886	1.905842	N/A	N/A	N/A	N/A	N/A	N/A	24.42515	1.67528
INU-72 h-1	N/A	N/A	N/A	6.334889	2.090556	N/A	N/A	N/A	N/A	N/A	N/A	25.52784	3.332579
INU-72 h-2	N/A	N/A	N/A	7.357271	1.016264	N/A	N/A	N/A	N/A	N/A	N/A	20.80715	2.394051
INU-72 h-3	N/A	N/A	N/A	7.887954	1.950595	N/A	N/A	N/A	N/A	N/A	N/A	20.39888	2.663116

Table S13 (continued)

Sample name	Glutathione	Glycine	Guanine	Guanosine	Guanosine 3',5'-cyclic monophosphoric acid	Guanosine 5'-monophosphoric acid	Histamine	Histidine	Histidinol
CUL-72 h-1	N/A	82.0443	N/A	0.4699257	N/A	N/A	N/A	9.107921	N/A
CUL-72 h-2	N/A	93.94559	N/A	0.6407618	N/A	N/A	N/A	7.585831	N/A
CUL-72 h-3	N/A	145.0713	N/A	0.9795844	N/A	N/A	N/A	7.814794	N/A
INU-72 h-1	N/A	69.85766	N/A	1.507572	N/A	N/A	N/A	9.628702	N/A
INU-72 h-2	N/A	30.19621	N/A	1.564425	N/A	N/A	N/A	15.8132	N/A
INU-72 h-3	N/A	206.6505	N/A	0.709838	N/A	N/A	N/A	24.00733	N/A

Sample name	Homocysteine	Homocystine	Hydroxytyrosol	Hyodeoxycholic acid	Hypoxanthine	Indole	Indole-3-acetic acid	Inosine	Isobutyric acid	Isocitric acid	Isoleucine	Isovaleric acid	Kynurenine	L-Cysteine S-sulfate
CUL-72 h-1	N/A	N/A	N/A	N/A	N/A	1328.535	N/A	N/A	12309.59	N/A	83.10458	2725.389	0.7406272	N/A
CUL-72 h-2	N/A	N/A	N/A	N/A	N/A	1036.26	N/A	N/A	18535.37	N/A	57.36452	8022.582	1.634312	N/A
CUL-72 h-3	N/A	N/A	N/A	N/A	N/A	1478.026	N/A	N/A	15148.3	N/A	77.3191	3813.614	1.227092	N/A
INU-72 h-1	N/A	N/A	N/A	N/A	N/A	1065.341	N/A	N/A	2089.072	N/A	3857.166	3799.128	1.42836	N/A
INU-72 h-2	N/A	N/A	N/A	N/A	N/A	1233.609	N/A	N/A	2351.808	N/A	3420.618	3112.827	1.442022	N/A
INU-72 h-3	N/A	N/A	N/A	N/A	N/A	1251.202	N/A	N/A	2461.907	N/A	5033.267	3533.386	1.471996	N/A

Sample name	Leucine	Lithocholic acid	Lysine	Maleic acid	Malic acid	Malonic acid	Methionine	Methionine sulfoxide	Methyl sulfate	Methyldopa	Mevalonic acid	N1,N12-Diacetylspermine
CUL-72 h-1	332.9505	N/A	16.802	N/A	N/A	3.52594	N/A	26.17407	N/A	N/A	N/A	0.685742
CUL-72 h-2	244.2249	N/A	18.12509	N/A	N/A	4.629817	N/A	24.42874	N/A	N/A	N/A	0.4902228
CUL-72 h-3	335.6487	N/A	15.2744	N/A	N/A	3.260579	N/A	25.52609	N/A	N/A	N/A	0.5629062
INU-72 h-1	11248.17	N/A	11.3888	N/A	N/A	3.29325	N/A	18.55026	N/A	N/A	N/A	0.5703015
INU-72 h-2	8732.448	N/A	16.69081	N/A	N/A	5.28722	N/A	24.68706	N/A	N/A	N/A	0.6965756
INU-72 h-3	12415.32	N/A	19.18698	N/A	N/A	4.87338	N/A	23.43797	N/A	N/A	N/A	0.7393308

Sample name	N1,N8-Diacetylspermidine	N1-Acetylspermidine	N1-Acetylspermine	N2-Phenylacetylglutamine	N-Acetyl-L-valine	N-Carbamoyl-L-aspartic acid	Nicotinamide	Nicotinamide adenine dinucleotide	Nicotinic acid	Noradrenaline
CUL-72 h-1	0.8729926	5.27841	N/A	N/A	232.895	N/A	1.406835	N/A	N/A	N/A
CUL-72 h-2	0.6415097	4.683516	N/A	N/A	205.8875	N/A	1.433566	N/A	N/A	N/A
CUL-72 h-3	1.03151	6.644439	N/A	N/A	228.6463	N/A	1.028196	N/A	N/A	N/A
INU-72 h-1	0.265548	3.38612	N/A	N/A	416.4463	N/A	1.393607	N/A	40.9654	N/A
INU-72 h-2	0.5087508	5.716911	N/A	N/A	390.0111	N/A	2.774888	N/A	26.22432	N/A
INU-72 h-3	0.9582755	9.747563	N/A	N/A	423.9652	N/A	1.956178	N/A	15.78185	N/A

Sample name	Norspermidine	N-α-Benzoylarginine ethylester	N-γ-Ethyl-L-glutamine	N-ε-Acetyl-L-lysine	Ophthalmic acid	Ornithine	Orotic acid	Oxidized glutathione	Pantothenic acid	Phenylalanine
CUL-72 h-1	N/A	0.08718549	N/A	2.567144	N/A	24.41048	N/A	N/A	N/A	94.78327
CUL-72 h-2	N/A	0.09346445	N/A	3.752591	N/A	6.854613	N/A	N/A	N/A	104.5619
CUL-72 h-3	N/A	0.09711846	N/A	2.197123	N/A	17.66643	N/A	N/A	N/A	120.2542
INU-72 h-1	N/A	0.0766585	N/A	15.9164	N/A	87.23355	N/A	N/A	N/A	2560.756
INU-72 h-2	N/A	0.08968639	N/A	5.490397	N/A	16.47757	N/A	N/A	N/A	2164.877
INU-72 h-3	N/A	0.1041871	N/A	6.690711	N/A	17.41198	N/A	N/A	N/A	3968.131

Table S13 (continued)

Sample name	Phenyllactic acid	Phenylpyruvic acid	Phe-Phe	Picolinic acid butyl ester	Pipecolic acid	Proline	Proline betaine	Propionic acid	Prostaglandin E2	Protocatechualdehyde	Protocatechuic acid
CUL-72 h-1	N/A	N/A	0.0787207	0.06028733	N/A	31.35064	0.1252512	38192.75	N/A	N/A	N/A
CUL-72 h-2	N/A	N/A	0.07210268	0.05812911	N/A	40.98071	0.2110236	57868.07	N/A	N/A	N/A
CUL-72 h-3	N/A	N/A	0.0764518	0.06302613	N/A	37.6562	0.2187502	47999.49	N/A	N/A	N/A
INU-72 h-1	N/A	N/A	0.0654012	0.04964816	N/A	17.79545	0.2403549	45484.77	N/A	N/A	N/A
INU-72 h-2	N/A	N/A	0.08441657	0.0614495	N/A	29.58167	0.1886041	47558.24	N/A	N/A	N/A
INU-72 h-3	N/A	N/A	0.07639587	0.05809583	N/A	34.56713	0.3608237	40937.45	N/A	N/A	N/A

Sample name	Putrescine	Pyridoxal 5'-phosphoric acid	Pyridoxine	Pyruvic acid	Riboflavin	Saccharic acid	S-Adenosylhomocysteine	S-Adenosylmethionine	Salicylic acid	Serine
CUL-72 h-1	13997.44	N/A	N/A	47.22609	N/A	N/A	N/A	N/A	N/A	43.68635
CUL-72 h-2	7576.822	N/A	N/A	36.73643	N/A	N/A	N/A	N/A	N/A	39.08611
CUL-72 h-3	14594.25	N/A	N/A	43.39414	N/A	N/A	N/A	N/A	N/A	50.6903
INU-72 h-1	11737.13	N/A	N/A	25.63359	N/A	N/A	N/A	N/A	N/A	16.87247
INU-72 h-2	11558.79	N/A	N/A	55.50747	N/A	N/A	N/A	N/A	N/A	33.40575
INU-72 h-3	12718.46	N/A	N/A	38.06107	N/A	N/A	N/A	N/A	N/A	152.2633

Sample name	Serine O-sulfate	Serotonin	Shikimic acid	Sinapic acid	Sorbitol 6-phosphate	Spermidine	Spermine	Succinic acid	Symmetric dimethylarginine	Taurine	Taurocholic acid
CUL-72 h-1	N/A	N/A	N/A	N/A	N/A	64.36331	4.081263	N/A	N/A	N/A	N/A
CUL-72 h-2	N/A	N/A	N/A	N/A	N/A	63.49862	2.218217	N/A	N/A	N/A	N/A
CUL-72 h-3	N/A	N/A	N/A	N/A	N/A	71.15579	3.052817	N/A	N/A	N/A	N/A
INU-72 h-1	N/A	N/A	N/A	N/A	N/A	39.99925	7.600389	N/A	N/A	N/A	N/A
INU-72 h-2	N/A	N/A	N/A	N/A	N/A	4.03273	0.6560286	N/A	N/A	N/A	N/A
INU-72 h-3	N/A	N/A	N/A	N/A	N/A	65.81897	4.277351	N/A	N/A	N/A	N/A

Sample name	Thiamine	Threonine	Thymidine	Thymidine 5'-monophosphoric acid	Thymine	Trimethylamine	Trimethylamine N-oxide	Tryptamine	Tryptophan	Tyramine
CUL-72 h-1	N/A	102.9494	N/A	N/A	N/A	568.8203	N/A	61.40158	7.615217	2017.394
CUL-72 h-2	N/A	95.80001	N/A	N/A	N/A	482.9735	N/A	228.8738	5.149752	1757.132
CUL-72 h-3	N/A	108.1486	N/A	N/A	N/A	567.28	N/A	59.38118	7.819109	2063.989
INU-72 h-1	N/A	110.6489	N/A	N/A	N/A	467.7804	N/A	37.64657	5.917668	1945.042
INU-72 h-2	N/A	98.64052	N/A	N/A	N/A	592.5556	N/A	58.01368	5.920179	1860.566
INU-72 h-3	N/A	112.2384	N/A	N/A	N/A	569.551	N/A	63.96225	11.07483	1934.634

Sample name	Tyrosine	Uracil	Uric acid	Uridine	Urocanic acid	Ursodeoxycholic acid	Valeric acid	Valine	Vanillic acid	Vanillin	Vanillylmandelic acid	Xanthine	α-Methylbenzylamine	γ-Butyrobetaine
CUL-72 h-1	22.41492	N/A	N/A	N/A	N/A	N/A	2786.322	391.5652	N/A	1.696698	N/A	N/A	128.9664	2.478093
CUL-72 h-2	13.76518	N/A	N/A	N/A	N/A	N/A	1469.88	343.8172	N/A	1.876151	N/A	N/A	211.1758	3.214697
CUL-72 h-3	14.46547	N/A	N/A	N/A	N/A	N/A	1869.982	388.8946	N/A	4.393737	N/A	N/A	131.6981	4.261286
INU-72 h-1	36.55685	N/A	N/A	N/A	N/A	N/A	338.4166	14647.18	N/A	4.197817	N/A	N/A	153.1842	6.241322
INU-72 h-2	64.13142	N/A	N/A	N/A	N/A	N/A	489.9766	14098.84	N/A	4.70034	N/A	N/A	134.3596	4.88168
INU-72 h-3	84.07243	N/A	N/A	N/A	N/A	N/A	444.2707	15309.62	N/A	1.441323	N/A	N/A	107.4943	7.260084
